# Synthesis of new enantiopure poly(hydroxy)aminooxepanes as building blocks for multivalent carbohydrate mimetics

**DOI:** 10.3762/bjoc.10.17

**Published:** 2014-01-20

**Authors:** Léa Bouché, Maja Kandziora, Hans-Ulrich Reissig

**Affiliations:** 1 Freie Universität Berlin, Institut für Chemie und Biochemie, Takustrasse 3, D-14195 Berlin, Germany

**Keywords:** aminooxepanes, carbohydrate mimetics, hydrogenolyses, Lewis acid-induced, lithiated allenes, nitrones, 1,2-oxazines, rearrangements, reductions

## Abstract

New compounds with carbohydrate-similar structure (carbohydrate mimetics) are presented in this article. Starting from enantiopure nitrones and lithiated TMSE-allene we prepared three 1,2-oxazine derivatives which underwent a highly stereoselective Lewis acid-induced rearrangement to give bicyclic products in good yield. Subsequent reductive transformations delivered a library of new poly(hydroxy)aminooxepane derivatives. The crucial final palladium-catalyzed hydrogenolysis of the 1,2-oxazine moiety was optimized resulting in a reasonably efficient approach to a series of new seven-membered carbohydrate mimetics.

## Introduction

Since carbohydrates play a crucial role in biochemistry, compounds mimicking their structure and/or function (carbohydrate mimetics) have attracted great attention in academic research and in drug development [1–3]. These mimetics should not have the drawbacks of carbohydrates such as low binding affinity or instability [4–5]. Many carbohydrates and their mimetics contain pyran rings, however, the corresponding ring-expanded compounds, oxepanes, have been investigated only in a limited number of studies. Several oxepane units can be found in natural products, in particular as toxins in marine organisms or plants [6–7]. A few reports in the literature deal with seven-membered polyhydroxylated ethers like septanosides (containing an anomeric center) [8] as well as compounds without an acetal moiety. Known methods for the construction of the seven-membered polyhydroxylated oxacycle, are for instance cycloaddition [9–11] or cyclodehydration of commercially available alcohols [12–14]. The disadvantages of these methods are the lack of selectivity as well as of stereocontrol and hence other routes are needed. One interesting option is the pathway via oxepines [15–18] and the subsequent dihydroxylation or direct reduction of their C=C double bond to give the corresponding oxepane derivatives [19–20]. Alternatively, oxepanes were also synthesized by the ring enlargement of their six-membered homologues [21–22]. Disappointingly, these methods often involve long reaction sequences and display sometimes restricted flexibility. A search for new stereoselective approaches is therefore highly desirable.

**Scheme 1:**
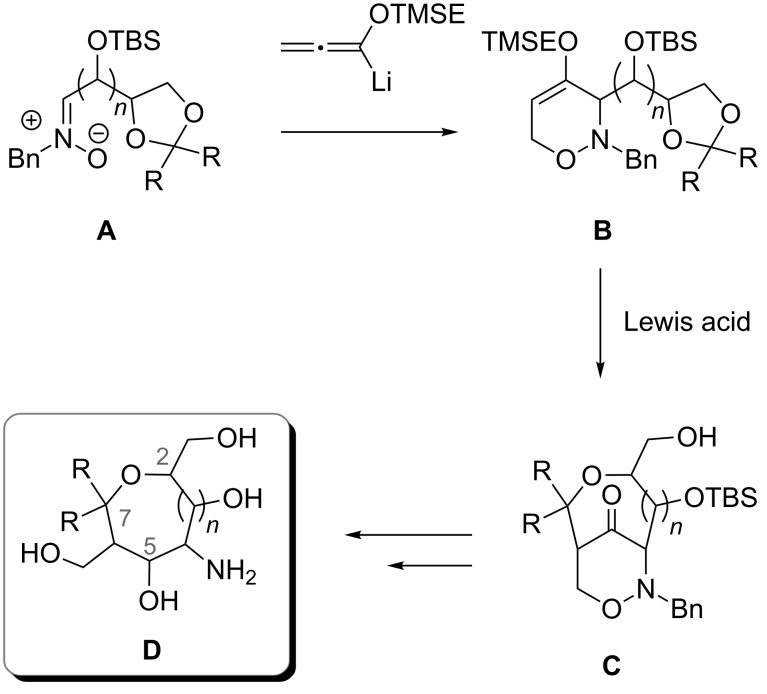
General approach to enantiopure the poly(hydroxy)aminopyrans **D** (*n* = 0) and the aminooxepanes **D** (*n* = 1) by [3 + 3]-cyclization of the (*Z*)-nitrones **A** with lithiated TMSE-allene followed by Lewis acid-induced rearrangement of 3,6-dihydro-2*H*-1,2-oxazines **B**. [TBS = *tert*-butyldimethylsilyl, TMSE = 2-(trimethylsilyl)ethyl]

During the last years our group systematically studied a new approach to carbohydrate mimetics **D** [23–28]. The general approach is shown in Scheme 1: the chiral pool-derived nitrones **A** [29–30] undergo a [3 + 3]-cyclization with lithiated [2-(trimethylsilyl)ethoxy]allene (TMSE-allene) [31] as C-3 building block [32] to form the 3,6-dihydro-2*H*-1,2-oxazines **B**; subsequent Lewis acid-promoted reactions [23] lead to the highly functionalized bicyclic 1,2-oxazinones **C** which can be regarded as protected aminopolyol precursors offering the option for plenty of selective transformations [27–28]; after reductive steps the polyhydroxylated cyclic ethers **D** are obtained. The advantages of this route are the stereocontrol and the flexibility concerning the chain length. In 2005, Al-Harrasi synthesized the first *tert*-butyldimethylsilyl (TBS)-protected aminopyrans (*n* = 0) and aminooxepanes (*n* = 1) via this reaction route [23]. Several of the poly(hydroxy)aminopyrans [24] were connected to gold nanoparticles and the resulting multivalent conjugates showed extremely high binding to P- and L-selectine [33–34]. For the planned biological testing of the corresponding aminooxepanes as components of multivalent conjugates, we required the fully deprotected compounds. Moreover, it was desirable to have additional derivatives with different configurations or functional groups. In this article, we therefore describe the full details of our route to a series of new enantiopure poly(hydroxy)aminooxepanes with variations at the 2-, 5- and 7-position of the seven-membered cyclic ether **D**.

## Results and Discussion

**Scheme 2:**
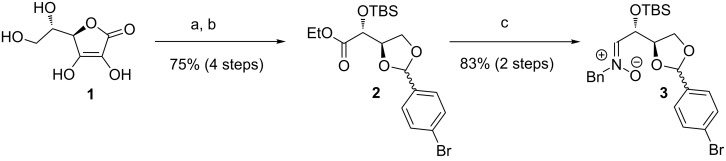
Synthesis of (*Z*)-nitrone **3**. Conditions: a) 1. *p*-Bromobenzaldehyde dimethylacetal, TFA, DMF, rt, 5 d; 2. 30% H_2_O_2_, K_2_CO_3_, H_2_O, 0 → 20 °C, 16 h; 3. EtI, MeCN, reflux, 20 h; b) TBSCl, imidazole, DMAP, CH_2_Cl_2_, 3 d, rt; c) 1. DIBAL-H, CH_2_Cl_2_, −78 °C, 3.5 h; 2. *N*-benzylhydroxylamine, MgSO_4_, CH_2_Cl_2_, rt, 16 h. [TFA = trifluoroacetic acid, DMAP = 4-(*N*,*N*-dimethylamino)pyridine, DIBAL-H = diisobutylaluminium hydride]

**Scheme 3:**
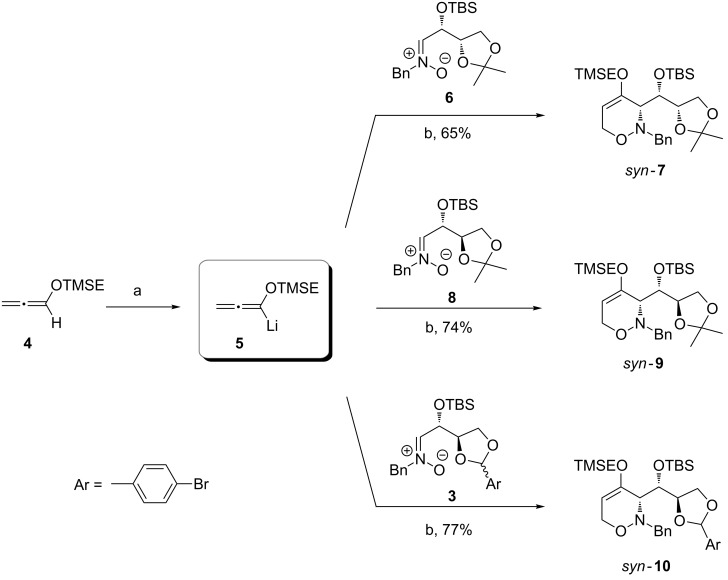
Synthesis of 1,2-oxazines *syn*-**7**, *syn*-**9** and *syn*-**10**. Conditions: a) *n*-BuLi, THF, −40 °C, 15 min; b) 1. THF, −78 °C, 1.5 to 3 h; 2. H_2_O, 1 h, −78 °C → rt. The *syn*:*anti* ratios were determined by ^1^H NMR spectroscopic analysis of the crude products and are all >95:5 (see Supporting Information File 1).

The enantiopure (*Z*)-nitrones **3**, **6** and **8** were synthesized essentially following known pathways [35] (Scheme 2 and Scheme 3). The new D-erythrose-derived nitrone **3** containing a *p*-bromophenyl moiety in the dioxolane ring was obtained in a straightforward manner (Scheme 2). Commercially available D-isoascorbic acid (**1**) was smoothly transformed in four steps into the ethyl ester **2**. After protection of the free 1,2-diol unit of **1** employing *p*-bromobenzaldehyde dimethylacetal, the ring double bond was oxidatively cleaved to give the sodium carboxylate which was directly transformed into the corresponding ethyl ester [36]. Protection with a TBS-group proceeded quantitatively to gain **2** in very good overall efficacy. Final transformation into the desired nitrone **3** was achieved in 83% yield (over two steps) by reduction of the ester using DIBAL-H at low temperature, followed by condensation with *N*-benzylhydroxylamine according to a Dondoni protocol [29]. This route allows the synthesis of enantiopure nitrone **3** in multi-gram scale.

The enantiopure (*Z*)-configured nitrones **6**, **8** and **3** were treated with in situ lithiated TMSE-allene at −78 °C furnishing the expected 1,2-oxazines *syn*-**7** [37], *syn*-**9** and *syn*-**10** in high yields and with excellent diastereoselectivities (Scheme 3). The nitrone **3** was used as a 96:4 mixture (with respect to the stereogenic center of the 1,3-dioxolane ring) and it could therefore provide four diasteromers of 1,2-oxazine *syn*-**10** (two major *syn*- and two minor *anti*-diasteromeres), but only a single *syn*-stereoisomer (with respect to C-3 of the 1,2-oxazine ring) was isolated after column chromatography. A product derived from the minor isomer of nitrone **3** was not detected and was apparently lost during the reaction or the purification. Since the stereogenic center of the 1,3-dioxolane ring is converted into an sp^2^-hybridized carbon during the Lewis acid-induced ring opening, the configuration of *syn*-**10** was not assigned at this center.

**Figure 1:**
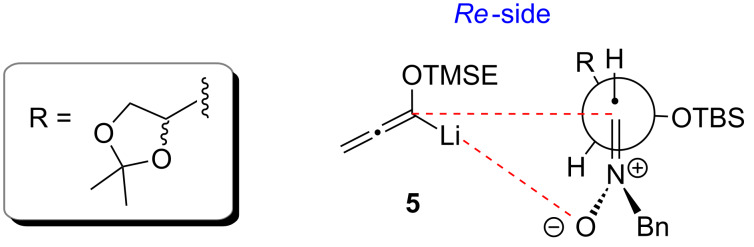
Proposed transition structure for the addition of lithiated TMSE-allene **5** to chiral nitrones **3**, **6** and **8**.

We discussed the mechanism of this [3 + 3]-cyclization in detail including possible side-reactions in an earlier report [31]. The observed high *syn*-diastereoselectivities are in accordance with our previously published results and were supported by an X-ray analysis of *syn*-**7** [37]. We conclude from our observations that the formation of 1,2-oxazines from nitrones **3**, **6** and **8** is mainly steered by the stereogenicity at the α-carbon (1,2-induction) [30–31], whereas the stereogenic center at the β-carbon (1,3-induction) has no or negligible influence. A few *syn*-selective additions of other nucleophiles to nitrones with two stereogenic centers similar to **6** or **8** were reported in the literature [38–39]. A preferred transition structure as depicted in Figure 1 plausibly explains our results. The silyloxy group at the α-carbon occupies an orthogonal position, the substituent R is close to the nitrone hydrogen substituent and the lithiated allene attacks from the Re-side of the nitrone (Felkin–Anh model for nitrones as proposed by Dondoni et al. [30]). An additional coordination of the lithium cation to the nitrone oxygen is possible.

**Scheme 4:**
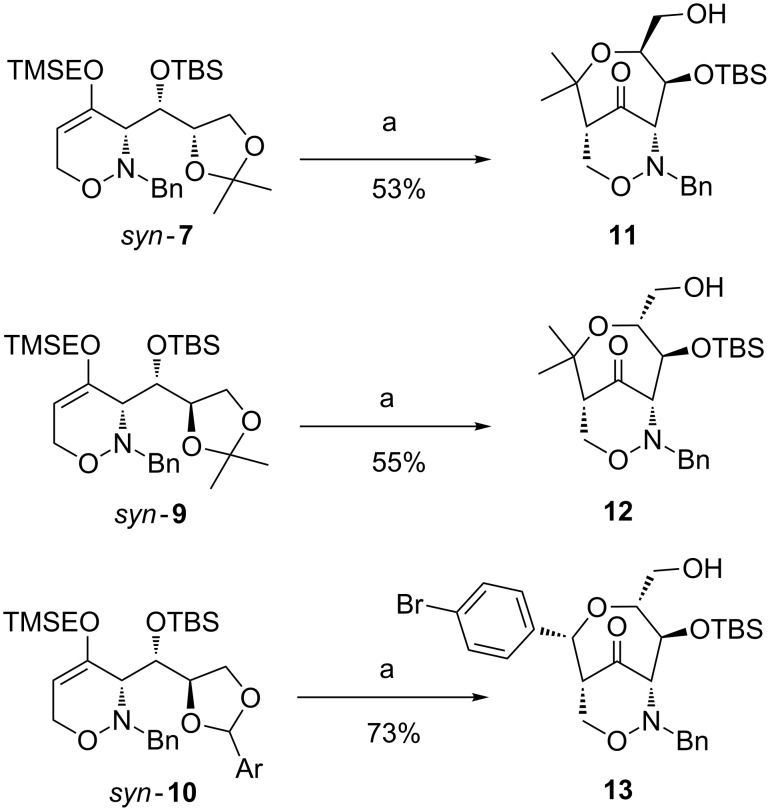
Synthesis of ketones **11**, **12** and **13** with a bicyclic 1,2-oxazine skeleton by Lewis acid-induced rearrangement. Conditions: a) 1. TMSOTf, CH_2_Cl_2_, −5 °C, 5 to 6 h; 2. aq NH_3_, rt, 10 min. [TMSOTf = trimethylsilyl trifluoromethanesulfonate]

The Lewis acid-induced rearrangements of 1,2-oxazines *syn*-**7**, *syn*-**9** and *syn*-**10** were achieved in moderate to good yields using TMSOTf as promoter (Scheme 4). A higher yield of 73% was achieved for the *p*-bromophenyl derivative **13** (isolated as a single diastereomer) which may be due to the stabilized carbenium ion formed at the benzylic position.

**Scheme 5:**
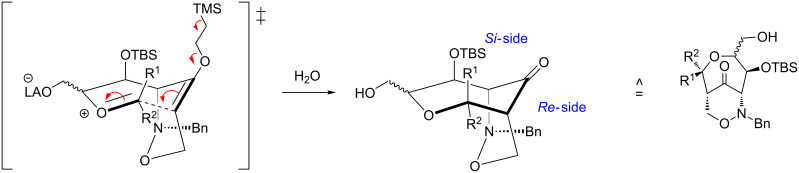
Proposed extended chair-like conformation with Zimmerman–Traxler-type transition state.

This rearrangement presents a relatively rare example of an intramolecular aldol-like reaction of an enol ether with an activated acetal (which may also be regarded as a special case of a Prins reaction) forming a seven-membered ring. We propose a Zimmerman–Traxler-type transition state (Scheme 5), placing the aryl group R^2^ (R^1^ = H) in the sterically more favorable pseudo-equatorial position in the reaction leading to compound **13** (for other Lewis acid-mediated formations of seven membered oxacycles, see references [40–41]).

**Figure 2:**
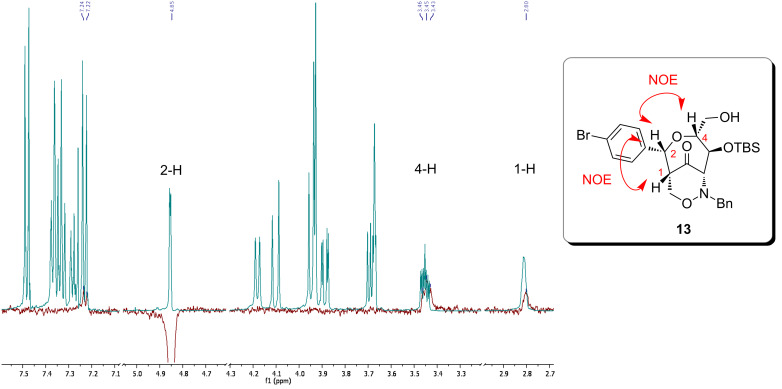
GOESY–NMR spectrum (CDCl_3_, 500 MHz) of bicyclic 1,2-oxazine **13**: irradiation of the 2-H proton. [GOESY = gradient enhanced nuclear Overhauser effect spectroscopy]

The relative configuration of the newly formed stereogenic center at the C-2 position of **13** was successfully determined by NOE experiments. By irradiation of the 2-H proton (Figure 2) several dipolar couplings with 1-H, 4-H and with one proton of the *p*-bromophenyl group were observed. For this reason, it is assumed that the 2-H proton of **13** is *cis*-orientated to the 1-H and 4-H protons and consequently shows an (*R)*-configuration.

**Scheme 6:**
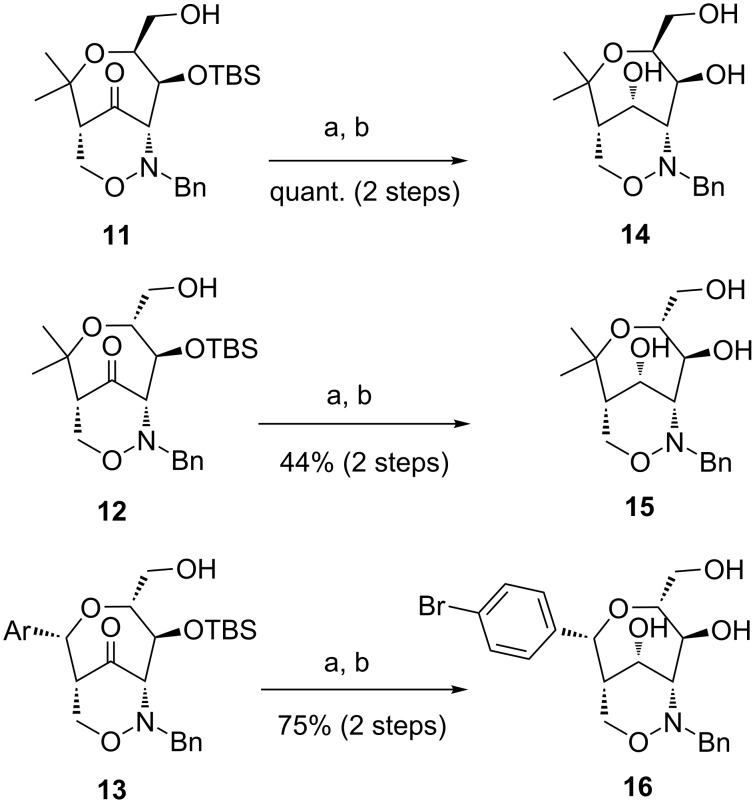
Synthesis of triols **14**, **15** and **16** by reduction of the carbonyl group and deprotection. Conditions: a) NaBH_4_, EtOH, 0 °C, 40 min to 16 h; b) TBAF (1 M THF), THF, 0 °C, 10 min to 3.5 h. [TBAF = tetra-*n*-butylammonium fluoride]

Ketones **11**, **12** and **13** were subsequently smoothly reduced with sodium borohydride in ethanol. In all cases, only one diastereomer (hydroxy group at C-10 with an *S*-configuration) was isolated (Scheme 6). These results are in accordance with previous observations obtained for the corresponding TBS-protected derivatives [42] suggesting that a preferred conformation of the seven-membered ring favors the hydride attack only from the *Re*-side. The *Si-*side (back-side) attack is probably hindered by the bulky OTBS group (also see Scheme 5). The configurational assignments are in accordance with NOE experiments performed with the alcohol derived from ketone **13** (precursor of **16**), where dipolar couplings have been observed between the 10-H and 9-H protons (CH_2_ next to the N–O bond). Finally, the TBS-groups were removed under standard conditions furnishing triols **14**, **15** and **16** in high yields.

**Scheme 7:**
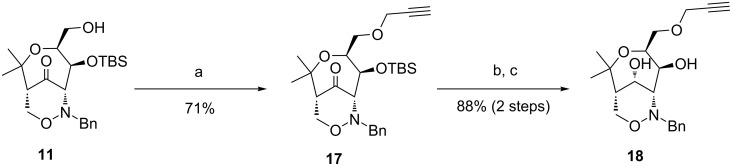
Synthesis of propargylic ether **18**. Conditions: a) propargyl bromide, NaOH, TBAI, H_2_O/CH_2_Cl_2_, −20 °C → rt, 7 d; b) NaBH_4_, EtOH, 0 °C, 4 h; c) TBAF (1 M THF), THF, 0 °C → rt, 3 d. [TBAI = tetra-*n*-butylammonium iodide]

The free primary hydroxy group of bicyclic ketone **11** was propargylated in 71% yield employing propargylic bromide under standard conditions (Scheme 7). After reduction of the carbonyl group [43] and TBS cleavage, propargylic ether **18** was obtained (88% yield over two steps) [44]. The alkyne moiety provides options for further transformations, e.g. Sonogashira reactions, Glaser couplings or 1,3-dipolar cycloadditions (click reactions) [45].

**Scheme 8:**
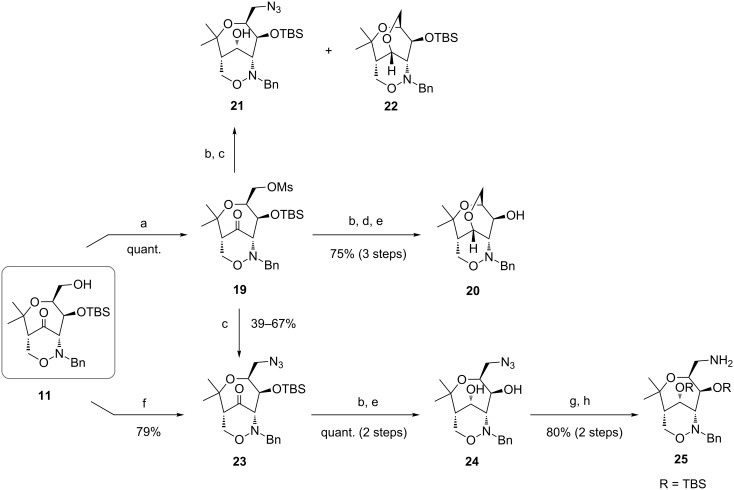
Synthesis of tricyclic compound **20**, bicyclic azide **24** and bicyclic amine **25**. Conditions: a) MsCl, Et_3_N, CH_2_Cl_2_, 0 °C → rt, 5 h; b) NaBH_4_, EtOH, 0 °C, 6 to 16 h; c) NaN_3_, DMF, reflux, 16 to 46 h; d) Et_3_N, DMF, reflux, 16 h; e) TBAF (1 M THF), THF, 0 °C → rt, 1 h; f) TPP, DIAD, DPPA, THF, −20 °C → rt, 2 d; g) TBSOTf, Et_3_N, CH_2_Cl_2_, 0 °C → rt, 3.5 h; h) TPP, THF/H_2_O, rt, 16 h. [Ms = methanesulfonate, TPP = triphenylphosphane, DIAD = diisopropyl azodicarboxylate, DPPA = diphenylphosphoryl azide]

We were also interested in the preparation of the bicyclic azide **24** and hence the primary hydroxy group of **11** was converted into mesylate **19** followed by a reduction of the carbonyl group (Scheme 8). The attempted nucleophilic substitution by sodium azide gave a mixture of two products: the desired TBS-protected azide **21** and an unexpected side product **22**. To overcome the formation of this side product, the introduction of the azido group was directly performed with mesylate **19**. Since the nucleophilic substitution of **19** into **23** gave only moderate yields with low reproducibility (from 39–67%), this step was optimized using Mitsunobu conditions with diphenylphosphoryl azide according to a protocol by Bose [46]. The desired bicyclic azide **23** was now isolated in 79% yield and its subsequent reduction and deprotection with TBAF gave compound **24** in essentially quantitative yield (over two steps). After protection of the two hydroxy substituents of **24** with TBS-groups, a Staudinger reaction was performed, which furnished bicyclic 1,2-oxazine derivative **25** with a primary amino group in 80% yield (over two steps).

Next we wanted to synthesize the unexpectedly formed unique tricyclic 1,2-oxazine **20** with higher efficacy. After reduction of **19** with sodium borohydride the intermediate alcohol was heated with triethylamine (to avoid the deprotection of the TBS-group) and compound **20** was obtained in 75% yield (over three steps).

**Scheme 9:**
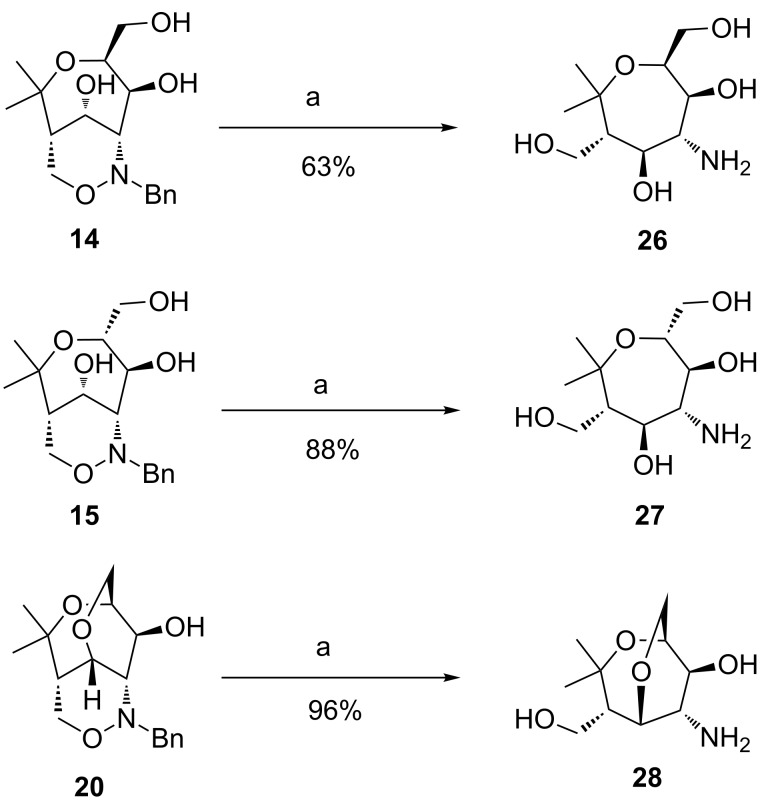
Hydrogenolyses of bicyclic and tricyclic 1,2-oxazines **14**, **15** and **20** to aminooxepanes **26**, **27** and **28**. Conditions: a) H_2_, Pd/C, MeOH, rt, 18 h.

The previously isolated unprotected triols **14**, **15** and **20** were subjected to standard hydrogenolysis conditions using palladium on charcoal in order to remove the *N*-benzyl group and to cleave the N–O bond [47–48] in one step. This process is often challenging due to the difficult control of the various reaction parameters and also because of the high polarity of the newly formed compounds. According to the experience of our group this step often leads to irreproducible results in particular in the case of the oxepane derivatives. This was confirmed by reductions performed with **14** and **15**. In Scheme 9, we present the best results obtained under these “standard conditions”. The aminooxepanes **26** and **27** [49] were isolated in 63% and 88% yield, respectively. Gratifyingly, the unique tricyclic 1,2-oxazine **20** was very efficiently converted into bicyclic aminooxepane derivative **28** (96% yield) without formation of side products.

**Figure 3:**
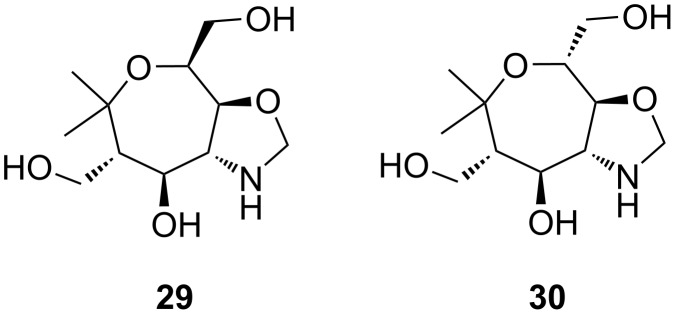
Proposed structures of the observed side products **29** and **30** during the hydrogenolyses of **14** and **15**.

The irreproducible results with **14** and **15** are probably due to the formation of side products such as **29** and **30** isolated in low quantities (proposed structures are presented in Figure 3). The ^1^H NMR spectra of all observed side products show signals at ca. 4.5 ppm, typically appearing as two symmetric doublets (AX system) with a coupling constant of 5.7 Hz. These observations suggest the presence of a methylene bridge at the aminooxepane skeleton. The position of the methylene bridge was only determined for side product **27** using HMBC NMR spectroscopy which shows a small coupling between one proton of the methylene bridge and C-3 (position of the alcohol). We assume that they are formed by in situ generated formaldehyde (dehydrogenation of methanol) [50–53] and subsequent aminal formation with aminooxepanes **26** and **27**. The aminoalcohol **28** seems not to form the corresponding compounds. We suppose that bicyclic compound **28** is more strained and hence the formation of a third ring may be unfavorable.

**Scheme 10:**
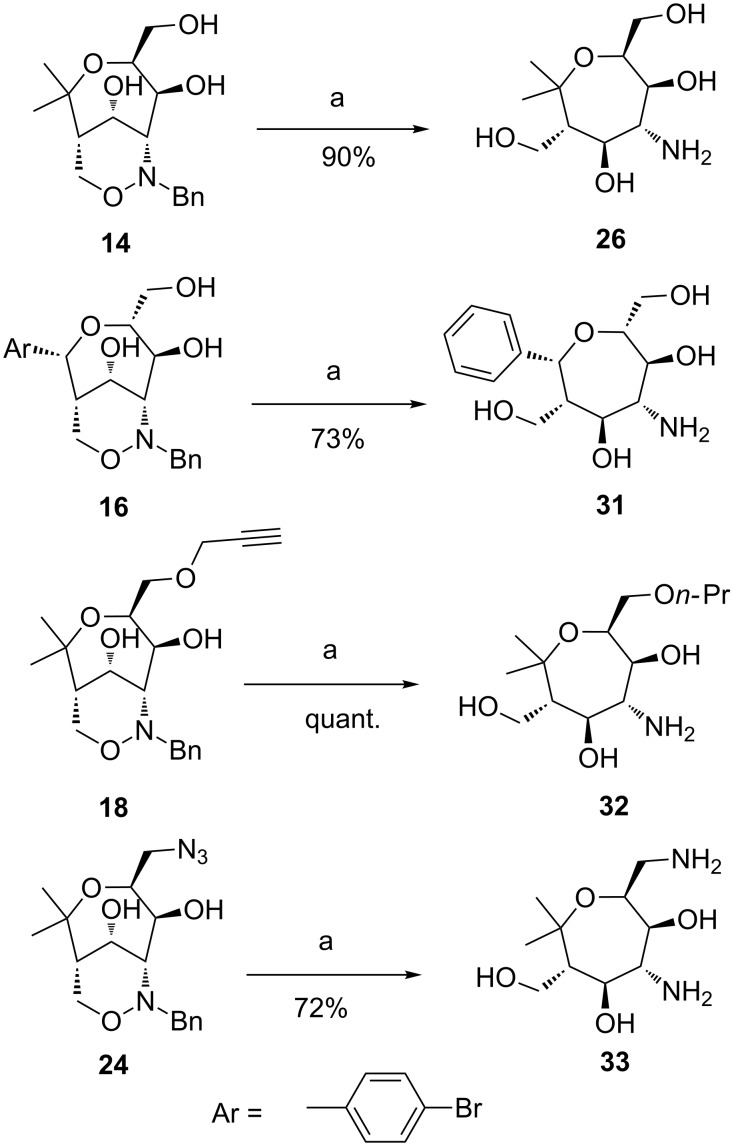
Hydrogenolyses of bicyclic 1,2-oxazines to aminooxepanes **26**, **31** and **32** and to diaminooxepane **33** under optimized conditions. Conditions: a) 1. H_2_, Pd/C, MeOH/AcOH = 5:1, rt, 17 h; 2. DOWEX^®^ H^+^; 3. aq NH_3_.

In order to avoid this side-reaction we performed hydrogenolysis experiments in alternative solvents, e.g. with ethanol or isopropanol, but small amounts of the corresponding side products were also observed in these experiments. Unpolar solvents were not suitable for the hydrogenolysis, probably due to the low solubility and conversion of the fairly polar starting materials or intermediates. After these problems we tried to diminish the nucleophilicity of the amino group by addition of acid since this protocol has proved to be advantageous in other reduction processes of amines [54]. Acetic acid in methanol (1:5) was a good medium for our reductions and substrate **14** was now smoothly reduced under hydrogen atmosphere with palladium on charcoal. After filtration through a pad of acidic DOWEX^®^ resin followed by elution with aqueous ammonia the clean poly(hydroxy)aminooxepane **26** was obtained in 90% yield (Scheme 10). Under these optimized reaction conditions other hydrogenolyses were studied. *p*-Bromophenyl-substituted triol **16** was transformed into the corresponding poly(hydroxy)aminooxepane derivative **31** in 73% yield. As expected, the bromo substituent of the aryl group was also reductively removed delivering a phenyl group in product **31**. Propargylic ether **18** was converted in quantitative yield into *n*-propyl ether **32**. Remarkably, the hydrogenolysis of bicyclic azide **24** smoothly furnished the diaminooxepane **33** in 72% yield. The results obtained using these new conditions are promising for other N–O cleavages of polar 1,2-oxazine derivatives. Furthermore, due to the newly formed amino group the prepared poly(hydroxy)aminooxepanes offer the option to selectively perform reductive aminations or Schotten–Baumann reactions. All these processes can lead to new carbohydrate mimetics, also in a multivalent fashion.

## Conclusion

In summary, the presented route employs the following key steps: Dondoni protocol for nitrone synthesis, [3 + 3]-cyclization with lithiated TMSE-protected allene, Lewis acid-induced rearrangement and reductive processes including a final hydrogenolysis. The new highly functionalized poly(hydroxy)aminooxepanes were thereby successfully synthesized in a highly stereocontrolled manner. The obtained compounds are potential carbohydrate mimetics and supplement the already studied six-membered homologues [33–34]. The introduction of new functional groups such as alkynyl or azido substituents at the bicyclic skeleton will also allow further transformations such as click reactions or palladium-catalyzed couplings. We expect that the newly prepared aminooxepanes or their multivalent conjugates will have interesting properties and possibly biological activities, e.g. as selectine inhibitors, which is currently investigated.

## Experimental

**General methods:** See Supporting Information File 2

### Typical procedure for the preparation of *syn*-1,2-oxazines by addition of lithiated TMSE-allene to chiral nitrones (procedure 1)

**(3*S*,1’*S*,4’’*S*)-2-Benzyl-1’-[(*tert*-butyldimethylsiloxy)-(2’’,2’’-dimethyl-1’’,3’’-dioxolan-4’’-yl)methyl]-4-[2’’’-(trimethylsilyl)ethoxy]-3,6-dihydro-2*H*-1,2-oxazine** (*syn*-**7**)**:** Analogous to literature [31], under argon atmosphere TMSE-allene **4** (5.00 g, 32.0 mmol) was dissolved in dry THF (60 mL). The slightly yellow solution was cooled to −40 °C and *n*-BuLi (2.5 M in hexanes; 10.2 mL, 25.6 mmol) was slowly added. After 15 min, the resulting strong-yellow mixture was cooled to −78 °C, and a solution of nitrone **6** (4.00 g, 10.5 mmol) in dry THF (60 mL) was added dropwise. The reaction mixture turned pink, then orange and later dark red. After stirring at this temperature for 2.5 h, the reaction mixture was quenched with water (40 mL) and left to warm to rt and then extracted with Et_2_O (3 × 65 mL). The combined organic layers were washed with brine, dried with Na_2_SO_4_, filtered through cotton and the solvent was removed in vacuo. Crude material [orange solid, 4.00 g; *syn:anti*-**7** >95:5 and corresponding diene (see Supporting Information File 2) <2%] was purified by column chromatography (silica gel, hexanes/EtOAc 17:0.5) to yield *syn*-**7** (3.69 g, 65%, lit. [23,37] 71%, *syn:anti*-**7** 94:6) as a pale-yellow solid; mp 102–105 °C; TLC (silica gel, hexanes/EtOAc 8:1) *R*_f_ 0.34; ^1^H NMR (700 MHz, CDCl_3_) δ 0.02 (s, 3H, SiMe), 0.06 (s, 9H, SiMe_3_), 0.08 (s, 3H, SiMe), 0.87 (s, 9H, Si*t-*Bu), 1.00, 1.09 (AB part of ABXY system, *J*_AX_ = 5.2 Hz, *J*_BY_ = 6.7 Hz, *J*_AY_ = 10.2 Hz, *J*_BX_ = 10.8 Hz, *J*_AB_ = 14.0 Hz, 1H each, 2’’’-H), 1.28, 1.34 (2 s, 3H each, Me), 2.78 (m_c_, 1H, 3-H), 3.12, 3.19 (AB part of ABX-system, *J*_AX_ = 6.6 Hz, *J*_BX_ ≈ *J*_AB_ = 7.8 Hz, 1H each, 5’’-H), 3.78, 3.83 (XY part of ABXY system, *J*_AX_ = 5.2 Hz, *J*_BY_ = 6.7 Hz, *J*_XY_ = 8.5 Hz, *J*_AY_ = 10.2 Hz, *J*_BX_ = 10.8 Hz, 1H each, 1’’’-H), 3.86 (d, *J* = 12.5 Hz, 1H, NCH_2_), 3.87 (dd, *J* = 3.5, 8.0 Hz, 1H, 1’-H), 4.14* (d, *J* = 12.5 Hz, 1H, NCH_2_), 4.14* (dd, *J* = 3.5, 15.1 Hz, 1H, 6-H), 4.25 (dt, *J* ≈ 6.6, 8.0 Hz, 1H, 4’’-H), 4.46 (td, *J* ≈ 2.0, 15.1 Hz, 1H, 6-H), 4.81 (dd, *J* = 2.0, 3.5 Hz, 1H, 5-H), 7.28, 7.33, 7.38 (3 m_c_, 1H, 2H, 2H, Ph) ppm, * overlapping signals; ESI–TOF (*m*/*z*): [M + H]^+^ calcd for C_28_H_50_NO_5_Si_2_, 536.3222; found, 536.3289. The analytical data are in accordance with literature [23,37]. Characteristic signals of *anti*-**7**: ^1^H NMR (700 MHz, CDCl_3_): δ 1.33, 1.36 (2 s, 3H each), 4.08 (t, *J* ≈ 6.3 Hz, 1H, 6-H), 4.29 (m_c_, 1H, 4’’-H), 4.76 (m_c_, 1H, 5-H) ppm.

### Typical procedure for the Lewis acid-induced rearrangement (procedure 2)

**(1*R*,4*S*,5*S*,6*S*)-7-Benzyl-5-(*tert*-butyldimethylsiloxy)-4-(hydroxymethyl)-2,2-dimethyl-3,8-dioxa-7-azabicyclo[4.3.1]decan-10-one** (**11**)**:** Analogous to literature [23,37], under argon atmosphere 1,2-oxazine *syn*-**7** (2.00 g, 3.73 mmol, *syn*:*anti* 94:6) was dissolved in dry CH_2_Cl_2_ (32 mL). The solution was cooled to −5 °C and TMSOTf (1.35 mL, 7.46 mmol) was slowly added. After 5.5 h stirring at 0 °C, the dark red mixture was quenched with a solution of aq ammonia (5%) turning yellow. Work-up was performed with CH_2_Cl_2_ (3 × 30 mL). The combined organic layers were washed with brine, dried with Na_2_SO_4_, filtered through cotton and the solvent was removed in vacuo. The crude material (orange oil, 2.05 g) was purified by column chromatography (silica gel, hexanes/EtOAc 5:1 to 4:1) to yield **11** (851 mg, 53%, lit. [23] 55%) as a pale-yellow oil; TLC (silica gel, hexanes/EtOAc 2:1) *R*_f_ 0.48; ^1^H NMR (500 MHz, CDCl_3_) δ −0.09, −0.02 (2 s, 3H each, SiMe), 0.86 (s, 9H, Si*t*-Bu), 1.37, 1.38 (2 s, 3H each, Me), 1.72 (bs, 1H, OH), 2.57 (m_c_, 1H, 1-H), 3.37 (m_c_, 1H, 6-H), 3.56, 3.67 (AB part of ABX system, *J*_AX_ = 4.5 Hz, *J*_BX_ = 8.1 Hz, *J*_AB_ = 10.9 Hz, 1H each, 4-CH_2_), 3.94 (A part of AB system, *J*_AB_ = 13.4 Hz, 1H, NCH_2_), 4.11–4.13 (m, 4H, 5-H, 9-H, NCH_2_), 4.58 (dd, *J* = 4.5, 8.1 Hz, 1H, 4-H), 7.26–7.29 (m, 5H, Ph) ppm; ESI-TOF (*m*/*z*): [M + H]^+^ calcd for C_23_H_38_NO_5_Si, 436.2514; found, 436.2553. The analytical data are in accordance with literature [23,37].

### Typical procedure for ketone reduction with NaBH_4_ (procedure 3)

**(1*S*,4*S*,5*S*,6*R*,10*S*)-7-Benzyl-5-(*tert*-butyldimethylsiloxy)-4-(hydroxymethyl)-2,2-dimethyl-3,8-dioxa-7-azabicyclo[4.3.1]decan-10-ol:** Under argon atmosphere ketone **11** (703 mg, 1.61 mmol) was dissolved in dry EtOH (26 mL). The solution was cooled to 0 °C and NaBH_4_ (119 mg, 3.15 mmol) was added in portions. After stirring at 0 °C for 4 h, the solvent was removed in vacuo. The crude material was dissolved in CH_2_Cl_2_ (20 mL) and then extracted with CH_2_Cl_2_ (3 × 25 mL). The combined organic layers were washed with brine, dried with Na_2_SO_4_, filtered through cotton and the solvent was removed in vacuo to yield the corresponding alcohol (704 mg, quant.) as a colorless solid; melting range 132–137 °C; [α]_D_^22^ −61.1 (*c* 0.63, CHCl_3_); TLC (silica gel, hexanes/EtOAc 2:1) *R*_f_ 0.58; ^1^H NMR (700 MHz, CDCl_3_) δ −0.09, −0.04 (2 s, 3H each, SiMe), 0.88 (s, 9H, Si*t*-Bu), 1.32, 1.53 (2 s, 3H each, Me), 1.75 (bs, 1H, 4-OH), 2.17 (m_c_, 1H, 1-H), 3.29 (dd, *J* = 2.5, 4.8 Hz, 1H, 6-H), 3.56 (m_c_, 1H, 4-CH_2_), 3.70 (B part of ABX system, *J*_AX_ = 8.5 Hz, *J*_AB_ = 10.7 Hz, 1H, 4-CH_2_), 3.81 (A part of ABX-system, *J*_AX_ = 3.0 Hz, *J*_AB_ = 12.0 Hz, 1H, 9-H), 3.87 (d, *J* = 13.9 Hz, 1H, NCH_2_), 3.93 (B part of ABX system, *J*_AB_ = 12.0 Hz, 1H, 9-H)*, 3.94 (d, *J* = 11.4 Hz, 1H, 10-OH), 4.17 (d, *J* = 13.9 Hz, 1H, NCH_2_), 4.33 (dt, *J* ≈ 1.0, 2.5 Hz, 1H, 5-H), 4.40-4.44 (m, 2H, 4-H, 10-H), 7.27–7.28, 7.32–7.36 (2 m, 5H, Ph) ppm, *no BX coupling present; ^13^C NMR (175 MHz, CDCl_3_) δ −4.9, −4.4 (2 q, SiMe), 18.1 (s, Si*C*Me_3_), 24.9 (q, Me), 25.9 (q, SiC*Me_3_*), 34.6 (q, Me), 47.4 (d, C-1), 58.4 (t, NCH_2_), 63.6 (t, 4-CH_2_), 64.5 (d, C-5), 68.6 (t, C-9), 71.1 (d, C-6), 72.8 (d, C-10), 75.7 (d, C-4), 78.3 (s, C-2), 127.6, 128.3, 128.6, 137.3 (3 d, s, Ph) ppm; IR (ATR) 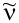: 3570, 3435 (OH), 3085–3030 (=C-H), 2990–2855 (C-H), 1250, 1215 (C-O), 1060, 1040 (C-O-C) cm^−1^; ESI–TOF (m/z): [M + H]^+^ calcd for C_23_H_40_NO_5_Si, 438.2670; found, 438.2702; anal. calcd for C_23_H_39_NO_5_Si (437.6): C, 63.12; H, 8.98; N, 3.20; found: C, 62.68; H, 8.96; N, 3.09.

### Typical procedure for deprotection using TBAF (procedure 4)

**(1*S*,4*S*,5*S*,6*R*,10*S*)-7-Benzyl-4-(hydroxymethyl)-2,2-dimethyl-3,8-dioxa-7-azabicyclo[4.3.1]decan-5,10-diol** (**14**)**:** The TBS-protected alcohol (200 mg, 0.457 mmol) was dissolved in THF (11 mL) and the solution was cooled to 0 °C. After addition of TBAF (1 M in THF; 0.9 mL, 0.90 mmol), the reaction mixture was stirred at this temperature for 10 min. Then the mixture was quenched with water (5 mL) and extracted with EtOAc (3 × 20 mL). The combined organic layers were washed with brine, dried with Na_2_SO_4_, filtered through cotton and the solvent was removed in vacuo. The crude material (yellow oil, 224 mg) was purified by column chromatography (silica gel, hexanes/EtOAc 1:2 to pure EtOAc) to yield **14** (135 mg, 91%) as a colorless solid; mp 50–53 °C; [α]_D_^22^ −52.5 (*c* 0.6, MeOH); TLC (silica gel, hexanes/EtOAc 1:3): *R*_f_ 0.27; ^1^H NMR (700 MHz, CD_3_OD) δ 1.31, 1.49 (2 s, 3H each, Me), 2.13 (m_c_, 1H, 1-H), 3.34 (dd, *J* = 3.0, 5.0 Hz, 1H, 6-H), 3.63, 3.71 (AB part of ABX system, *J*_AX_ = 3.5 Hz, *J*_BX_ = 6.5 Hz, *J*_AB_ = 11.0 Hz, 1H each, 4-CH_2_), 3.74 (dd, *J* = 3.0, 12.8 Hz, 1H, 9-H), 3.91 (d, *J* = 14.1 Hz, 1H, NCH_2_), 3.96 (d, *J* = 12.8 Hz, 1H, 9-H), 4.12 (d, *J* = 14.1 Hz, 1H, NCH_2_), 4.44–4.47 (m, 3H, 4-H, 5-H, 10-H), 7.22, 7.29, 7.35 (3 m_c_, 1H, 2H, 2H, Ph) ppm; ^13^C NMR (175 MHz, CD_3_OD) δ 24.6, 34.5 (2 q, Me), 48.7 (d, C-1), 49.9 (d, C-5), 59.4 (t, NCH_2_), 63.6 (t, 4-CH_2_), 65.9 (t, C-9), 70.0 (d, C-4), 74.7 (d, C-6), 76.3 (d, C-10), 79.1 (s, C-2), 128.1, 129.2, 129.4, 139.2 (3 d, s, Ph) ppm; IR (ATR) 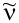: 3360 (OH), 3085–3030 (=C-H), 2970–2860 (C-H), 1215 (C-O), 1060 (C-O-C) cm^−1^; ESI–TOF (*m*/*z*): [M + Na]^+^ calcd for C_17_H_25_NO_5_Na, 346.1630; found, 346.1622; anal. calcd for C_17_H_25_NO_5_ (323.4): C, 63.14; H, 7.79; N, 4.33; found: C, 63.20; H, 7.83; N, 4.31.

**Synthesis of (1*R*,4*S*,5*S*,6*S*)-4-(azidomethyl)-7-benzyl-5-(*tert*-butyldimethylsiloxy)-2,2-dimethyl-3,8-dioxa-7-azabicyclo[4.3.1]decan-10-one** (**23**)**:** Under argon atmosphere TPP (99 mg, 0.38 mmol) was dissolved in dry THF (1.5 mL) and the solution was cooled to −20 °C. DIAD (76.5 mg, 0.378 mmol) was added and the reaction mixture became milky white. After 10 min stirring at that temperature, alcohol **11** (150 mg, 0.344 mmol in 0.6 mL of dry THF) was slowly added and the mixture was stirred for further 30 min at −20 °C. The now milky-yellow solution was progressively warmed to 0 °C and DPPA (89.0 μL, 0.413 mmol) was added. After stirring for 6 h at 0 °C and 1 d at rt, the reaction mixture was quenched with water (2 mL) and extracted with EtOAc (3 × 5 mL). The combined organic layers were washed with brine, dried with Na_2_SO_4_, filtered through cotton and the solvent was removed in vacuo. Crude material (yellow oil, 479 mg) was purified by column chromatography (silica gel, hexanes to hexanes/EtOAc 8:1) to yield azide **23** (125 mg, 79%) as a colorless solid; mp 117–121 °C; [α]_D_^22^ +22.7 (*c* 1.01, CHCl_3_); TLC (silica gel, hexanes/EtOAc 3:1) *R*_f_ 0.73; ^1^H NMR (500 MHz, CDCl_3_) δ −0.11, −0.03 (2 s, 3H each, SiMe), 0.86 (s, 9H, Si*t*-Bu), 1.37, 1.42 (2 s, 3H each, Me), 2.55 (t, *J* ≈ 2.8 Hz, 1H, 1-H), 3.05 (dd, *J* = 4.3, 12.3 Hz, 1H, 4-CH_2_), 3.36 (dd, *J* = 1.1, 2.8 Hz, 1H, 6-H), 3.45 (dd, *J* = 8.7, 12.3 Hz, 1H, 4-CH_2_), 3.95 (A part of AB system, *J*_AB_ = 13.5 Hz, 1H, NCH_2_), 4.05 (m_c_, 1H, 5-H), 4.14 (B part of AB system, *J*_AB_ = 13.5 Hz, 1H, NCH_2_), 4.18 (m_c_, 2H, 9-H), 4.61 (ddd, *J* = 0.8, 4.3, 8.7 Hz, 1H, 4-H), 7.27-7.35 (m, 5H, Ph) ppm; ^13^C NMR (125 MHz, CDCl_3_) δ −4.8, −4.6 (2 q, SiMe), 18.2 (s, Si*C*Me_3_), 22.4 (q, Me), 25.8 (q, SiC*Me_3_*), 31.6 (q, Me), 53.2 (t, 4-CH_2_), 58.1 (d, C-1), 58.7 (t, NCH_2_), 67.9 (t, C-9), 69.4 (d, C-5), 74.2 (d, C-4), 75.0 (s, C-2), 75.1 (d, C-6), 127.9, 128.66, 128.71, 136.2 (3 d, s, Ph), 199.8 (s, C-10) ppm; IR (ATR) 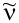: 3090–3030 (=C-H), 2855–2995 (C-H), 2095 (N_3_), 1710 (C=O), 1495 (C=C), 1295, 1255 (C-O), 1190–1075 (C-O-C) cm^−1^; ESI–TOF (*m*/*z*): [M + H]^+^ calcd for C_23_H_37_N_4_O_4_Si, 461.2579; found, 461.2607.

### Typical procedure for hydrogenolysis with Pd/C in AcOH/MeOH (procedure 5)

**(2*S*,3*S*,4*R*,5*S*,6*S*)-4-Amino-2,6-bis(hydroxymethyl)-7,7-dimethyloxepan-3,5-diol** (**26**)**:** Bicyclic compound **14** (100 mg, 0.309 mmol) was dissolved in dry MeOH (10 mL) and AcOH (2 mL). After addition of Pd/C (100 mg, 0.094 mmol Pd) the suspension was saturated with hydrogen at rt for 1 h followed by stirring under hydrogen pressure (balloon). After reaction completion 18 h, the mixture was filtered through Celite^®^, washed with EtOH and the solvents were removed in vacuo. The obtained salt was dissolved in water and filtered through a DOWEX^®^ column (H_2_O) and washed with water until the complete acid was removed (control with pH paper). Then the column was washed with aq NH_3_ (2 to 5%) to yield the free amine **26** (65 mg, 90%) as a colorless solid; mp 121–125 °C; [α]_D_^22^ +10.2 (*c* 1.35, MeOH); TLC [silica gel, MeCN/aq NH_3_ (25%) 5:1] *R*_f_ 0.10; ^1^H NMR (700 MHz, CD_3_OD) δ 1.19, 1.34 (2 s, 3H each, Me), 1.77 (ddd, *J* = 3.0, 6.5, 9.5 Hz, 1H, 6-H), 2.89 (dd, *J* = 6.9, 8.8 Hz, 1H, 4-H), 3.52–3.55 (m, 2H, 2-CH_2_, 3-H), 3.59 (B part of ABX system, *J*_BX_ = 5.0 Hz, *J*_AB_ = 11.5 Hz, 1H, 2-CH_2_), 3.65, 3.68 (AB part of ABX system, *J*_AX_ = 3.0 Hz, *J*_BX_ = 6.5 Hz, *J*_AB_ = 11.3 Hz, 1H each, 6-CH_2_), 3.71 (m_c_, 2H, 2-H, 5-H) ppm; ^13^C NMR (175 MHz, CD_3_OD) δ 21.1, 31.5 (2 q, Me), 60.0 (d, C-6), 63.6 (t, 2-CH_2_), 64.1 (d, C-4), 64.2 (t, 2-CH_2_), 71.3, 73.8 (2 d, C-2, C-5), 76.5 (d, C-3), 77.1 (s, C-7) ppm; IR (ATR) 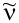: 3530–3310 (OH, NH_2_), 2960–2860 (C-H), 1455, 1470, 1140 (C-O) cm^−1^; ESI–TOF (*m*/*z*): [M + Na]^+^ calcd for C_10_H_21_NO_5_Na, 258.1320; found, 258.1317; anal. calcd for C_10_H_21_NO_5_ (235.3): C, 51.05; H, 9.00; N, 5.95; found, C, 50.78; H, 8.97; N: 5.59.

## Supporting Information

File 1 Experimental procedures.

File 2 Characterization data ^1^H NMR and ^13^C NMR spectra of synthesized compounds.
