# Supplementary material for: Synthesis of new enantiopure poly(hydroxy)aminooxepanes as building blocks for multivalent carbohydrate mimetics
Source: Beilstein J Org Chem. 2014 Jan 20;10:213–23. doi: 10.3762/bjoc.10.17 (PMC3943604; doi:10.3762/bjoc.10.17)
Supplement: File 1 — Experimental procedures. [file Beilstein_J_Org_Chem-10-213-s001.pdf]

Supporting Information File 1

for

Synthesis of new enantiopure poly(hydroxy)aminooxepanes as building blocks for multivalent carbohydrate mimetics

Léa Bouché, Maja Kandziora and Hans-Ulrich Reissig*

Address: Freie Universität Berlin, Institut für Chemie und Biochemie, Takustrasse 3, D-14195 Berlin, Germany

Email: Hans-Ulrich Reissig - hans.reissig@chemie.fu-berlin.de

* Corresponding author

Experimental procedures

Table of contents:

- General information s2
- Additional experimental procedures and analytical data s4
- References s37

General information

Reactions were generally performed under inert atmosphere (argon) in flame-dried flasks. Solvents and reagents were added by syringe. Solvents were dried using standard procedures and were purified with a MB SPS-800-dry solvent system. Triethylamine was distilled from CaH_2 and stored over KOH under argon atmosphere. Commercial available reagents were used as received without further purification unless otherwise stated. Products were purified by flash chromatography on silica gel (230–400 mesh, Merck or Fluka). For ion exchange columns DOWEX® (50WX8-200, Sigma-Aldrich) was used. Before use, the column was washed with appropriate solvents (e.g. H_2O, EtOH or MeOH) until the filtrate became clear. Unless otherwise stated, yields refer to analytical pure samples. Hydrogenolyses were performed with hydrogen from Air Liquide (Alphagaz 2). The reaction outcome sometimes depended on the Pd/C charges, the reaction times applied, the used hydrogen bottle or the stirring speed leading occasionally to irreproducible hydrogenation results.

TLC-analyses were performed on silica gel coated aluminium plates purchased from Merck. Products were detected by UV-activity and by using staining reagents (Cer/molybdenum reagent, $KMnO_4$ and ninhydrine). NMR spectra were recorded on BRUKER (AV 500, AV 700) and JEOL (ECP 500, ECX 400) instruments. Chemical shifts (δ) are listed in parts per million (ppm) and are reported relative to solvent residual signals: $CDCl_3$ (^1H: δ = 7.26 ppm, ^{13}C: δ = 77.16 ppm), CD_3OD (^1H: δ = 3.31 ppm, ^{13}C: δ = 49.00 ppm) or benzene-d_6 (^1H: δ = 7.16 ppm, ^{13}C: δ = 128.06 ppm). Integrals are in accordance with assignments; coupling constants (*J*) are given in Hz. All ^{13}C NMR spectra are proton–decoupled. Multiplicity is indicated as follows: s

(singlet), bs (broad singlet), d (doublet), t (triplet), q (quartet), dd (doublet of doublet), dt (doublet of triplet), td (triplet of doublet), m (multiplet), m_c (centered multiplet). For detailed peak assignments 2D spectra were measured (COSY, HMQC, HMBC and NOESY). The given ratios of diastereomers were calculated by comparison of the 2'-H peaks (for bromophenyl derivatives) and 5-H peaks (for *syn*-/*anti* 1,2-oxazines). IR spectra were measured with a Jasco spectrometer (FT/IR-4100 with DLATGS-Detector). HRMS analyses were performed with Agilent 6210 (ESI–TOF, 10 µL/min, 1.0 bar, 4 kV) and Varian/Agilent Ionspec QFT-7 (ESI–FTICR, 4 µL/min, 1.0 bar, 4 kV) instruments. Elemental analyses were carried out with instruments from PerkinElmer (CHN-Analyzer 2400) and from Elementar (Vario, Vario EL, Vario EL III). Melting points were measured with a Reichert apparatus (Thermovar) and are uncorrected.

Additional experimental procedures and analytical data

The following compounds were prepared analogously to literature procedures: TMSE-allene **4** [1], *N*-benzylhydroxylamine [2], nitrone **6** [3], and nitrone **8** [3].

Synthesis of (2*R*,2'*S*,4'*R*)-ethyl 2-[2'-(*p*-Bromophenyl)-1',3'-dioxolan-4'-yl]-2-hydroxy ethanoate and (2*R*,2'*R*,4'*R*)-diastereomer:

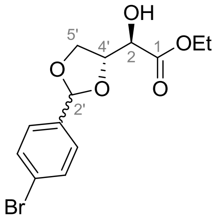

Analogous to the literature [4], D-isoascorbic acid (**1**, 18.3 g, 104 mmol) was dissolved in dry DMF (40 mL) under argon atmosphere and *p*-bromobenzaldehyde dimethylacetal (20 mL, 120 mmol) and TFA (0.62 mL, 9.40 mmol) were added. After five days stirring at rt, a 50% aq solution of NaCl (100 mL) and EtOAc (100 mL) were added. The phases were separated and the organic layer was washed with a 50% aq solution of NaCl (2 × 100 mL). The combined organic layers were dried with Na_2SO_4, filtered through cotton and the solvent was removed in vacuo. The resulting yellowish oil was then suspended in an aq K_2CO_3 solution (25.7 g, 186 mmol in 110 mL H_2O), cooled to 0 °C and a 30% aq solution of H_2O_2 (23 mL) was carefully added. The reaction mixture was stirred at 20 °C overnight and the solvent was removed in vacuo. The colorless wet solid was poured in hot EtOH, filtered to remove insoluble salts and the solvent was removed in vacuo. The obtained potassium carboxylate was suspended in MeCN (92 mL) and ethyl iodide (12.6 mL, 156 mmol) was added. The reaction mixture was heated to reflux and stirred at that temperature for 20 h.

After cooling to rt, CH_2Cl_2 (250 mL) and a 10% aq $NaCO_3$ solution (250 mL) were added. The phases were separated and the aq layer was extracted with CH_2Cl_2 (3 × 250 mL). The combined organic layers were dried with Na_2SO_4, filtered through cotton and the solvent was removed in vacuo. The obtained crude material was purified by column chromatography (silica gel, hexanes/EtOAc 5:1) to yield the *p*-bromophenyl protected ethyl ester (26.0 g, 75% over 3 steps, d.r. 52:48) as colorless crystals; mp 69–72 °C; $[\alpha]_D^{22}$ +2.1 (*c* 1.2, $CHCl_3$); TLC [silica gel, hexanes/EtOAc = 4:1] R_{f1} 0.04; R_{f2} 0.14; Signals for the major Diastereomere are assigned with *: ^1H NMR (500 MHz, $CDCl_3$): δ 1.26 (t, J = 7.2 Hz, 3 H, CH_3CH_2O), 1.31 (t, J = 7.1 Hz, 3 H, CH_3CH_2O*), 3.06 (d, J = 6.6 Hz, 1 H, OH), 3.13 (d, J = 5.5 Hz, 1 H, OH*), 4.03-4.07 (m, 2 H, 5'-H, 5'-H*), 4.12 (dd, J = 6.5, 8.4 Hz, 1 H, 5'-H*), 4.20-4.27 (m, 3 H, CH_3CH_2O, 5'-H), 4.28-4.31 (m, 3 H, CH_3CH_2O*, 2-H), 4.41-4.50 (m, 3 H, 2-H*, 4'-H, 4'-H*), 5.74 (s, 1 H, 2'-H), 5.96 (s, 1 H, 2'-H*), 7.33 (AB part of AA'BB' system, J_{AB} = 8.4 Hz, 2 H, Ar*), 7.39 (AB part of AABB system, J_{AB} = 8.5 Hz, 2 H, Ar), 7.497§ (A'B' part of AA'BB' system, $J_{A'B'}$ = 8.4 Hz, 2 H, Ar*), 7.50§ (A'B' part of AA'BB' system, $J_{A'B'}$ = 8.5 Hz, 2 H, Ar) ppm, §signals overlapping; ^{13}C NMR (125 MHz, $CDCl_3$): δ 14.2, 14.3 (2 q, CH_3CH_2O, CH_3CH_2O*)§, 62.3, 62.4 (2 t, CH_3CH_2O, CH_3CH_2O*)§, 65.8 (t, C-5'*), 66.7 (t, C-5'), 71.4 (d, C-2*), 71.3 (d, C-2), 77.0, 77.6 (2 d, C-4', C-4'*)§, 104.0 (d, C-2'*), 104.1 (d, C-2'), 123.5, 123.8 (2 s, Ar, Ar*)§, 128.3 (d, Ar*), 128.6 (d, Ar), 131.6, 131.62 (2 d, Ar, Ar*)§, 135.9, 136.8 (2 s, Ar, Ar*)§, 172.0, 172.1 (2 s, C-1, C-1*)§; §the signals of the diastereomer could not be assigned; IR (ATR) $\tilde{\nu}$: 3360 (O-H), 3090-3045 (=C-H), 2985-2880 (C-H), 1710 (C=O), 1595 (C=C), 1215 (C-O), 1100-1070 (C-O-C) cm^{-1}; ESI-TOF (m/z): $[M + Na]^+$ calcd for $C_{13}H_{15}BrO_5Na$, 352.9972; found, 353.0001; Anal. calcd for $C_{13}H_{15}BrO_5$ (331.2): C, 47.15; H, 4.57; found: C, 47.47; H, 4.35.

Synthesis of (2*R*,2'*S*,4'*R*)-Ethyl 2-[2'-(*p*-bromophenyl)-1',3'-dioxolan-4'-yl)-2-(*tert*-butyldimethylsiloxy) ethanoate and (2*R*,2'*R*,4'*R*)-diastereomer (2):

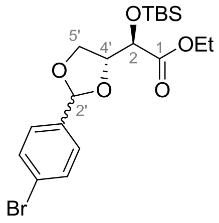

The above described *p*-bromophenyl protected ethyl ester (10.0 g, 30.2 mmol, d.r. 77:23) was dissolved in CH_2Cl_2 (61 mL) and the solution was cooled to 0 °C under argon atmosphere. Imidazole (3.69 g, 54.3 mmol) and DMAP (184 mg, 1.50 mmol) were added. Afterwards a solution of TBSCl (5.27 g, 35.0 mmol in 4.6 mL CH_2Cl_2) was added dropwise. The reaction mixture was stirred for 1 d at rt. TLC analysis indicated incomplete conversion of the starting material. Additional amounts of imidazole (1.85 g, 27.2 mmol), DMAP (92 mg, 7.5 mmol) and TBSCl (2.6 g, 17.3 mmol in 2 mL CH_2Cl_2) were added and stirring at rt was continued for 2 d. After addition of brine (30 mL) the reaction mixture was extracted with CH_2Cl_2 (3 × 50 mL). The combined organic layers were dried with Na_2SO_4, filtered through cotton and the solvent was removed in vacuo. The obtained crude material (orange oil, 16.0 g) was purified by column chromatography (silica gel, pure hexanes to hexanes/EtOAc 50:1 to 25:1) to yield TBS-protected ethyl ester **2** (13.5 g, quant., d.r. 83:17) as a colorless oil; $[\alpha]_D^{22}$ +23.7 (*c* 0.42, $CHCl_3$); TLC [silica gel, hexanes/EtOAc = 4:1] R_{f1} 0.49; R_{f2} 0.58; Signals of the major diastereomer are assigned with *: ^1H NMR (500 MHz, $CDCl_3$): δ 0.04, 0.06 (2 s, 3 H each, SiMe), 0.12, 0.13 (2 s, 3 H each, SiMe*), 0.88 (s, 9 H, Si*t*-Bu), 0.94 (s, 9 H, Si*t*-Bu*), 1.27 (t, *J* = 7.1 Hz, 3 H, CH_3CH_2O), 1.29 (t, *J* = 7.1 Hz, 3 H, CH_3CH_2O*), 4.04 (dd, *J* = 7.0, 8.4 Hz, 1 H, 5'-H), 4.12-4.25 (m, 8 H, CH_3CH_2O, CH_3CH_2O*, 2-H, 5'-H, 5'-H*), 4.41 (ddd, *J* = 4.7, 6.1, 7.0 Hz, 1 H, 4'-H),

4.48-4.52 (m, 2 H, 2-H*, 4'-H*), 5.73 (s, 1 H, 2'-H), 5.89 (s, 1 H, 2'-H*), 7.33 (AB part of AA'BB' system, J_{AB} = 8.4 Hz, 2 H, Ar*), 7.37 (AB part of AA'BB' system, J_{AB} = 8.4 Hz, 2 H, Ar), 7.498§ (A'B' part of AA'BB' system, $J_{A'B'}$ = 8.4 Hz, 2 H, Ar*), 7.50§ (A'B' part of AA'BB' system, $J_{A'B'}$ = 8.4 Hz, 2 H, Ar) ppm, §signals overlapping; ^{13}C NMR (125 MHz, $CDCl_3$): δ -5.0¥, -4.9¥ (2 q, SiMe, SiMe*)§, 14.2 (q, CH_3CH_2O), 14.3 (q, CH_3CH_2O*), 18.3 (s, Si*C*Me₃), 18.4 (s, Si*C*Me₃*), 25.7 (q, SiC*Me₃*), 25.8 (q, SiC*Me₃**), 61.27 (t, CH₃*C*H₂O), 61.3 (t, CH₃*C*H₂O*), 66.0 (t, C-5'*), 67.0 (t, C-5'), 72.8 (d, C-4'*), 73.0 (d, C-4'), 77.0 (d, C-2*), 77.7 (d, C-2), 103.85 (d, C-2'), 103.9 (d, C-2'*), 123.5 (s, Ar*), 123.6 (s, Ar), 128.3 (d, Ar*), 128.6 (d, Ar), 131.5 (d, Ar), 131.7 (d, Ar*), 135.6 (s, Ar), 136.9 (s, Ar*), 171.0, (s, C-1*), 171.4 (s, C-1); §the signals of the diastereomer could not be assigned; ¥overlapping of 2 signals; IR (ATR) $\tilde{\nu}$: 3055 (=C-H), 2955-2860 (C-H), 1730, 1750 (C=O), 1595, 1600 (C=C), 1255 (C-O), 1150-1010 (C-O-C) cm^{-1}; ESI-TOF (m/z): $[M + Na]^+$ calcd for $C_{19}H_{29}BrO_5SiNa$, 469.0840; found, 469.0840; Anal. calcd for $C_{19}H_{29}BrO_5Si$ (445.4): C, 51.23; H, 6.58; found: C, 51.18; H, 6.60.

Synthesis of (*Z*)-*N*-benzyl-*N*-[(2*S*,4'*R*,2'*S*)-{2-[2'-(*p*-bromophenyl)-1',3'-dioxolan-4'-yl]-2-(*tert*-butyldimethylsiloxy)ethyliden}aminoxide and (2*S*,4'*R*,2'*R*)-diastereomer (3):

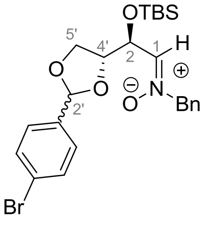

Analogous to the literature [3], ester **2** (5.00 g, 11.2 mmol, d.r. 83:17) was dissolved in dry CH_2Cl_2 (22 mL) and the solution was cooled to −78 °C under argon

atmosphere. DIBAL-H (80% in toluene, 9.8 mL) was added dropwise. After stirring the reaction mixture for 3.5 h at that temperature, an aq solution of potassium sodium tartrate (40 mL) was added and the emulsion was stirred for 2.5 h. The resulting two phases were separated and the aq layer was extracted with CH_2Cl_2 (3 × 35 mL). The combined organic layers were washed with brine, dried with Na_2SO_4, filtered through cotton and the solvent was removed in vacuo to give the aldehyde (4.51 g, 11.2 mol, d.r. 86:14) as a colorless liquid that was used without purification for the next step. The aldehyde was dissolved in CH_2Cl_2 (71 mL). Afterwards, $MgSO_4$ (3.64 g, 30.2 mmol) and *N*-benzylhydroxylamine (3.18 g, 25.9 mmol) were added and the resulting slightly yellow mixture was stirred at rt overnight. The reaction mixture was then filtered through cotton and the solvent was removed in vacuo. The obtained crude material (yellow oil, 12.3 g) was purified by column chromatography (silica gel, hexanes/EtOAc 3:1) to yield nitrone **3** (4.70 g, 83% over 2 steps, d.r. 86:14) as a yellow oil; $[\alpha]_D^{22}$ +13.1 (*c* 0.85, $CHCl_3$); TLC [silica gel, hexanes/EtOAc = 1:2] R_f 0.68; Signals of the major diastereomer are assigned with *: ^{1}H NMR (500 MHz, $CDCl_3$): δ -0.04 (s, 3 H, SiMe), -0.01 (s, 3 H, SiMe*), 0.04 (s, 3 H, SiMe), 0.09 (s, 3 H, SiMe*), 0.82 (s, 9 H, Si*t*-Bu), 0.85 (s, 9 H, Si*t*-Bu*), 3.96-4.00 (m, 2 H, 5'-H, 5'-H*), 4.05-4.10 (m, 2 H, 5'-H, 5'-H*), 4.35 (dt, *J* ≈ 4.9, 7.2 Hz, 1 H, 4'-H), 4.43* (dt, *J* ≈ 4.8, 6.6 Hz, 1 H, 4'-H*), 4.84, 4.85 (AB system, J_{AB} = 13.6 Hz, 1 H each, NCH_2), 4.89 (s, 2 H, NCH_2*), 5.13 (dd, *J* = 4.9, 6.8 Hz, 1 H, 2-H), 5.20 (dd, *J* = 4.8, 6.1 Hz, 1 H, 2-H*), 5.59 (s, 1 H, 2'-H), 5.85 (s, 1 H, 2'-H*), 6.62 (d, *J* = 6.8 Hz, 1 H, 1-H), 6.63 (d, *J* = 6.1 Hz, 1 H, 1-H*), 7.29 (AB part of AA'BB' system, J_{AB} = 8.4 Hz, 2 H, Ar*), 7.32 (AB part of AA'BB' system, J_{AB} = 8.4 Hz, 2 H, Ar), 7.33-7.39 (m, 5 H, Ph), 7.40 (s, 5 H, Ph*), 4.46 (A'B' part of AA'BB' system, $J_{A'B'}$ = 8.4 Hz, 2 H, Ar), 4.48 (A'B' part of AA'BB' system, $J_{A'B'}$ = 8.4 Hz, 2 H, Ar*) ppm; ^{13}C NMR (125 MHz, $CDCl_3$): δ -5.0, -4.9, -4.8,

-4.7 (4 q, SiMe, SiMe*)§, 18.1¥, 25.8¥ (s, q, SiCMe$_3$, SiCMe$_3$*), 66.7, 66.8 (2 t, C-5', C-5'*)§, 67.3 (d, C-2), 68.1 (d, C-2*), 70.15 (t, NCH$_2$*), 70.20 (t, NCH$_2$), 76.8 (d, C-4'*), 78.1 (d, C-4'), 103.8 (d, C-2'), 103.9 (d, C-2'*), 123.4, 123.5 (2 s, Ar, Ar*)§, 128.4, 128.7, 129.1, 129.2, 129.3, 129.4, 129.46, 129.49 (8 d, Ar, Ar*, Ph, Ph*)§, 131.5, 131.6 (2 d, Ph, Ph*)§, 132.4, 132.5, 136.1, 137.0 (4 s, Ar, Ar*, Ph, Ph*)§, 137.7, 137.8 (2 d, C-1, C-1*)§ ppm; ¥overlapping of 2 signals; §the signals of the diastereomer could not be assigned; IR (ATR) $\tilde{\nu}$: 3085-3030 (=C-H), 2950-2855 (C-H), 1690-1560 (C=C, C=N), 1280 (N-O), 1255 (C-O) cm^{-1}; ESI-TOF (m/z): [M + Na]$^+$ calcd for $C_{24}H_{32}BrNO_4SiNa$, 528.1176; found 528.1158.

Synthesis of (3*S*,1'*S*,4''*R*)-2-benzyl-1'-[(*tert*-butyldimethylsiloxy)-(2'',2''-dimethyl-1'',3''-dioxolan-4''-yl)-methyl]-4-[2'''-(trimethylsilyl)ethoxy]-3,6-dihydro-2*H*-1,2-oxazine (*syn*-**9**):

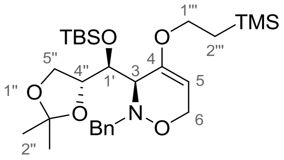

Following typical procedure 1, TMSE-allene (3.00 g, 19.2 mmol), *n*-BuLi (2.5 M in hexanes; 5.1 mL, 12.8 mmol) and nitrone **8** (2.00 g, 5.27 mmol, dissolved in 6.3 mL THF) were reacted in THF (30 mL) for 2.5 h. The obtained crude material (orange oil, 3.00 g, *syn*/*anti* > 95:5) was purified by column chromatography (silica gel, hexanes/EtOAc 18:1) to yield 1,2-oxazine *syn*-**9** (2.08 g, 74%, lit. [5]: 49%, *syn*/*anti* = 97:3) as a yellow oil; TLC [silica gel, hexanes/EtOAc = 1:1] R_f 0.97; ^{1}H NMR (700 MHz, $CDCl_3$): δ 0.03 (s, 3 H, SiMe), 0.07 (s, 9 H, SiMe$_3$), 0.09 (s, 3 H, SiMe), 0.88 (s, 9 H, Si*t*-Bu), 1.01, 108 (AB part of ABXY system, J_{AX} = 5.0 Hz, J_{BY} = 6.6 Hz, J_{AY} = 10.6 Hz, J_{BX} = 11.0 Hz, J_{AB} = 14.0 Hz, 1 H each, 2'''-H), 1.25, 1.39 (2 s, 3 H each,

Me), 2.92 (dd, J = 1.7, 4.4 Hz, 1 H, 3-H), 3.76, 3.82 (XY part of ABXY system, J_{BY} = 6.6 Hz, J_{XY} = 9.4 Hz, J_{AX} = 5.0 Hz, J_{AY} = 10.6 Hz, J_{BX} = 11.0 Hz, 1 H each, 1'''-H), 3.88 (d, J = 13.2 Hz, 1 H, NCH_2), 3.98, 4.00 (AB part of ABX system, J_{AX} = 6.4 Hz, J_{BX} = J_{AB} = 7.4 Hz, 1 H each, 5''-H), 4.09 (ddd, J = 3.0, 6.4, 7.4 Hz, 1 H, 4''-H), 4.15 (dd, J = 3.5, 15.1 Hz, 1 H, 6-H), 4.14 (d, J = 13.2 Hz, 1 H, NCH_2), 4.28 (dd, J = 3.0, 4.4 Hz, 1 H, 1'-H), 4.42 (td, $J \approx$ 2.0, 15.1 Hz, 1 H, 6-H), 4.77 (dd, J = 2.0, 3.5 Hz, 1 H, 5-H), 7.25, 7.31, 7.39 (3 m_c, 1 H, 2 H, 2 H, Ph) ppm; ESI-TOF (m/z): $[M + H]^+$ calcd for $C_{28}H_{50}NO_5Si_2$, 536.3222; found, 536.3231. The analytical data are in accordance with the literature [5].

Synthesis of (3*S*,1'*S*,4''*R*)-2-benzyl-1'-[2''-(*p*-bromphenyl)-1'',3''-dioxolan-4''-yl]-[(*tert*-butyldimethylsiloxy)methyl]-4-[2'''-(trimethylsilyl)ethoxy]-3,6-dihydro-2*H*-1,2-oxazine (*syn*-**10**)**:**

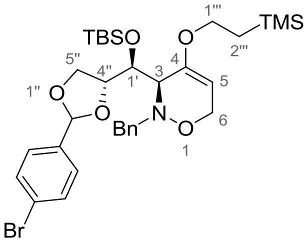

Following typical procedure 1, TMSE-allene (4.92 g, 31.4 mmol), *n*-BuLi (2.5 M in hexanes; 11.3 mL, 28.2 mmol) and nitrone **3** (5.29 g, 10.4 mmol, d.r. 96:4, dissolved in 14 mL THF) were reacted in THF (70 mL) for 1.5 h. The obtained crude material (yellow oil, 10.1 g, *syn*/*anti* > 95:5) was purified twice by column chromatography (silica gel, hexanes/EtOAc 100:1 to 45:1) to yield 1,2-oxazine *syn*-**10** (5.29 g, 77%, *syn*/*anti* > 95:5) as a yellow oil and the corresponding diene [6] (175 mg, 3%, ca. 90% pure) as a colorless oil; $[\alpha]_D^{22}$ +13.1 (*c* 0.85, $CHCl_3$); TLC [silica gel, hexanes/EtOAc = 45:1] R_f 0.39; ^1H NMR (700 MHz, $CDCl_3$): δ 0.06 (s, 9 H, $SiMe_3$),

0.08, 012 (2 s, 3 H each, SiMe), 0.92 (s, 9 H, Si*t*-Bu), 0.98, 1.06 (AB part of ABXY system, J_{AX} = 5.0 Hz, J_{BY} = 6.4 Hz, J_{AY} = 10.8 Hz, J_{BX} = 11.3 Hz, J_{AB} = 13.0 Hz, 1 H each, 2'''-H), 2.84 (dd, *J* = 1.7, 5.3 Hz, 1 H, 3-H), 3.73, 3.81 (XY part of ABXY system, J_{YA} = 6.4 Hz, J_{XY} = 9.5 Hz, J_{XA} = 5.0 Hz, J_{YA} = 10.8 Hz, J_{XB} = 11.3 Hz, 1 H each, 1'''-H), 3.85 (d, *J* = 13.1 Hz, 1 H, NCH_2), 4.08-4.22 (m, 4 H, 6-H, 4''-H, 5''-H), 4.15 (d, *J* = 13.1 Hz, 1 H, NCH_2), 4.41 (td, *J* ≈ 1.9, 15.4 Hz, 1 H, 6-H), 4.44 (dd, *J* = 2.3, 5.3 Hz, 1 H, 1'-H), 4.76 (dd, *J* = 1.9, 3.2 Hz, 1 H, 5-H), 5.87 (s, 1 H, 2''-H), 7.30 (AB part of AA'BB' system, J_{AB} = 8.4 Hz, 2 H, Ar), 7.25, 7.30, 7.37 (3 m_c, 1 H, 2 H, 2 H, Ph), 7.48 (A'B' part of AA'BB' system, $J_{A'B'}$ = 8.4 Hz, 2 H, Ar) ppm; ^{13}C NMR (175 MHz, $CDCl_3$): δ -4.5, -4.2 (2 q, SiMe), -1.2 (q, $SiMe_3$), 17.5 (t, C-2'''), 18.3 (s, Si*C*Me_3), 26.1 (q, SiC*Me_3*), 57.3 (t, NCH_2), 60.0 (t, C-6), 63.7 (d, C-3), 64.0 (t, C-1'''), 66.0 (t, C-5''), 73.1 (d, C-1'), 78.2 (d, C-4''), 92.2 (d, C-5), 102.6 (d, C-2''), 123.0 (s, Ar), 127.5, 128.2, 128.4, 129.2 (4 d, Ar, Ph), 131.5 (d, Ar), 136.9, 138.1 (2 s, Ar, Ph), 148.1 (s, C-4) ppm; IR (ATR) $\tilde{\nu}$: 3085-3015 (=C-H), 2955-2855 (C-H), 1675 (C=C), 1250 (C-O) cm^{-1}; ESI-TOF (m/z): $[M + Na]^+$ calcd for $C_{32}H_{48}BrNO_5Si_2Na$, 684.2127; found 684.2139; Anal. calcd for $C_{32}H_{48}BrNO_5Si_2$ (662.8): C, 57.99; H, 7.30; N, 2.11; found: C, 58.41; H, 7.34; N, 1.75.

(5*S*,4'*R*)-5-(*tert*-Butyldimethylsiloxy)-5-[2'-(*p*-bromophenyl)-1',3'-dioxolan-4'-yl)]-3-[2''-(trimethylsilyl)ethoxy]-penta-1,3-diene:

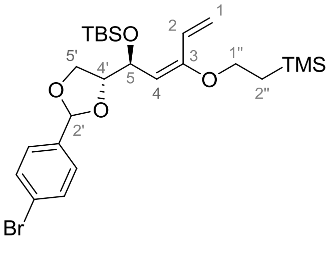

colorless oil; $[\alpha]_D^{22}$ +20.7 (*c* 1.1, $CHCl_3$); TLC [silica gel, hexanes/EtOAc = 45:1] R_f 0.73; in analogy to previous results [7,8] an (*E*)-configuration of the C-3/C-4 double bond was assumed: ^{1}H NMR (500 MHz, $CDCl_3$): δ 0.02 (s, 3 H, SiMe), 0.05 (s, 9 H, $SiMe_3$), 0.09 (s, 3 H, SiMe), 0.90 (s, 9 H, Si*t*-Bu), 1.07 (m_c, 2 H, 2''-H), 3.78-3.86 (m, 2 H, 1''-H), 4.09-4.13 (m, 1 H, 5'-H), 4.14-4.39 (m, 2 H, 4'-H, 5'-H), 4.45 (d, *J* = 9.1 Hz, 1 H, 4-H), 4.79 (dd, *J* = 4.4, 9.1 Hz, 1 H, 5-H), 5.22 (dt, *J* = 1.7, 11.0 Hz, 1 H, 1-H), 5.71 (dd, *J* = 1.7, 16.9 Hz, 1 H, 1-H), 5.88 (s, 1 H, 2'-H), 6.47 (dd, *J* = 11.0, 16.9 Hz, 1 H, 2-H), 7.38, 7.49 (AA'BB' system, *J* = 8.3 Hz, 2 H each, Ar) ppm; ^{13}C NMR (125 MHz, $CDCl_3$): δ -4.5, -3.8 (2 q, SiMe), -1.2 (q, $SiMe_3$), 17.4 (t, C-2''), 18.2, 25.9 (s, q, $SiCMe_3$), 64.7 (t, C-1''), 66.7 (t, C-5'), 69.9 (d, C-5), 80.2 (d, C-4'), 102.5 (d, C-4), 103.7 (d, C-2'), 116.7 (t, C-1), 128.1 (s, Ar), 128.3 (d, C-2), 128.4, 131.6, 137.2 (2 d, s, Ar), 152.7 (s, C-3) ppm; IR (ATR) $\tilde{\nu}$: 2950-2855 (C-H), 1725, 1640-1590, 1485-1470 (C=C), 1260, 1245, 1080, 1010 (C-O) cm^{-1}; ESI-TOF (m/z): $[M + Na]^+$ calcd for $C_{25}H_{41}BrNO_4Si_2Na$, 563.1619; found 563.1649;

Synthesis of (1*R*,4*R*,5*S*,6*S*)-7-benzyl-5-(*tert*-butyldimethylsiloxy)-4-(hydroxy-methyl)-2,2-dimethyl-3,8-dioxa-7-azabicyclo[4.3.1]decan-10-one (12):

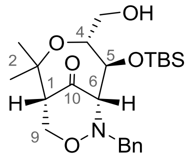

Following typical procedure 2, 1,2-oxazine *syn*-**9** (100 mg, 0.187 mmol, *syn*/*anti* = 97:3) and TMSOTf (69 μL, 0.37 mmol) were reacted in CH_2Cl_2 (1.6 mL) for 5 h. After 4 h, additional TMSOTf (34 μL) was added. The obtained crude material (yellow oil, 84 mg) was purified by column chromatography (silica gel, hexanes/EtOAc 5:1 to 4:1) to yield ketone **12** (45 mg, 55%) as a pale yellow oil; TLC [silica gel, hexanes/EtOAc

6:1] R_f 0.13; ^{1}H NMR (500 MHz, $CDCl_3$): δ 0.07, 0.08 (2 s, 3 H each, SiMe), 0.88 (s, 9 H, Si*t*-Bu), 1.32, 1.34 (2 s, 3 H each, Me), 2.13 (t, *J* ≈ 5.7 Hz, 1 H, OH), 2.38 (ddd, *J* = 1.5, 2.6, 6.5 Hz, 1 H, 1-H), 3.52-3.59 (m, 3 H, 4-CH_2, 5-H, 6-H), 3.73 (t, *J* ≈ 8.2 Hz, 1 H, 4-CH_2), 3.90 (A part of AB system, J_{AB} = 14.0 Hz, 1 H, NCH_2), 4.02 (dd, *J* = 1.3, 8.2 Hz, 1 H, 4-H), 4.05 (B part of AB system, J_{AB} = 14.0 Hz, 1 H, NCH_2), 4.16 (dd, *J* = 6.5, 12.0 Hz, 1 H, 9-H), 4.46 (dd, *J* = 2.6, 12.0 Hz, 1 H, 9-H), 7.26-7.27, 7.34, 7.39 (m, 2 m_c, 1 H, 2 H, 2 H, Ph) ppm; ESI-TOF (m/z): $[M + Na]^+$ calcd for $C_{23}H_{37}NO_5SiNa$, 458.2333; found, 458.2342. The analytical data are in accordance with the literature [5].

Synthesis of (1*R*,2*R*,4*R*,5*S*,6*S*)-7-benzyl-2-(*p*-bromophenyl)-5-(*tert*-butyldimethylsiloxy)-4-(hydroxymethyl)-3,8-dioxa-7-azabicyclo[4.3.1]decan-10-one (13):

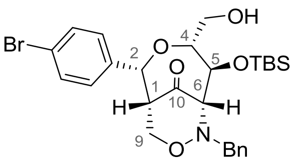

Following typical procedure 2, 1,2-oxazine *syn*-**10** (1.20 g, 1.81 mmol, *syn*/*anti* = 97:3) and TMSOTf (0.7 mL, 3.8 mmol) were reacted in CH_2Cl_2 (29 mL) for 6 h. The obtained crude material (yellow oil, 1.02 g) was purified by column chromatography (silica gel, hexanes/EtOAc 50:1 to 8:1) to yield ketone **13** (742 mg, 73%) as a pale yellow solid; mp 55–59 °C; $[\alpha]_D^{22}$ +163 (*c* 0.78, $CHCl_3$); TLC [silica gel, hexanes/EtOAc 7:1] R_f 0.33; ^{1}H NMR (500 MHz, $CDCl_3$): δ 0.10, 0.12 (2 s, 3 H each, SiMe), 0.90 (s, 9 H, Si*t*-Bu), 2.00 (bs, 1 H, OH), 2.81 (m_c, 1 H, 1-H), 3.45 (ddd, *J* = 2.9, 6.4, 9.1 Hz, 1 H, 4-H), 3.68 (m_c, 1 H, 6-H), 3.68, 3.89 (AB part of ABX system, J_{AX} = 2.9 Hz, J_{BX} = 6.4 Hz, J_{AB} = 11.6 Hz, 1 H each, 4-CH_2), 3.93-3.96* (m, 2 H, 9-H), 3.94*, 4.10 (AB system, J_{AB} = 14.0 Hz, 1 H each, NCH_2), 4.18 (d, *J* = 9.1 Hz, 1 H, 5-

H), 4.85 (d, J = 2.3 Hz, 1 H, 2-H), 7.23 (AB part of AA'BB' system, J_{AB} = 8.5 Hz, 2 H, Ar), 7.25-7.39 (m, 5 H, Ph), 7.48 (A'B' part of AA'BB' system, $J_{A'B'}$ = 8.5 Hz, 2 H, Ar) ppm, *signals overlapping; ^{13}C NMR (125 MHz, $CDCl_3$): δ -4.5, -3.8 (2 q, SiMe), 18.0, 25.8 (s, q, $SiCMe_3$), 55.1 (d, C-1), 58.9 (t, NCH_2), 64.1 (t, 4-CH_2), 66.2 (t, C-9), 67.7 (d, C-5), 81.3 (d, C-6), 83.7 (d, C-2), 85.7 (d, C-4), 121.9, 127.5 (s, d, Ar), 127.7, 128.56, 128.58 (3 d, Ph), 131.8 (d, Ar), 136.5, 137.9 (2 s, Ar, Ph), 204.2 (s, C-10) ppm; IR (ATR) $\tilde{\nu}$: 3490 (O-H), 3090-3020 (=C-H), 2950-2855 (C-H), 1720 (C=O), 1590, 1490 (C=C), 1250 (C-O), 1140-1010 (C-O-C) cm^{-1}; ESI-TOF (m/z): $[M + Na]^+$ calcd for $C_{27}H_{38}BrNO_5SiNa$, 584.1438; found, 584.1412; Anal. calcd for $C_{27}H_{38}BrNO_5Si$ (562.6): C, 57.64; H, 6.46; N, 2.49; found: C, 57.63; H, 6.49; N, 2.44.

Reduction of 12: (1*S*,4*R*,5*S*,6*R*,10*S*)-7-benzyl-5-(*tert*-butyldimethylsiloxy)-4-(hydroxymethyl)-2,2-dimethyl-3,8-dioxa-7-azabicyclo[4.3.1]decan-10-ol

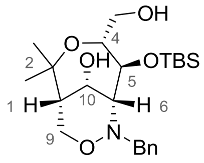

Following typical procedure 3, ketone **12** (1.70 g, 3.90 mmol) and $NaBH_4$ (329 mg, 8.71 mmol) were reacted in dry EtOH (67 mL) overnight at 0 °C to rt. The obtained crude material (yellow oil, 1.90 g) was purified by column chromatography (silica gel, hexanes/EtOAc 4:1) to yield the corresponding secondary alcohol (962 mg, 57%) as a colorless solid; mp 103–105 °C; $[\alpha]_D^{22}$ +17.5 (*c* 1.53, $CHCl_3$); TLC [silica gel, hexanes/EtOAc 2:1] R_f 0.58; ^{1}H NMR (500 MHz, $CDCl_3$): δ 0.03, 0.11 (2 s, 3 H each, SiMe), 0.88 (s, 9 H, Si*t*-Bu), 1.31, 1.63 (2 s, 3 H each, Me), 1.77 (m_c, 1 H, 1-H), 2.28 (m_c, 1 H, 4-OH), 2.49 (d, J = 2.5 Hz, 1 H, 10-OH), 3.35 (d, J ≈ 5.5 Hz, 1 H, 6-H), 3.50

(t, $J \approx 8.5$ Hz, 1 H, 4-CH_2), 3.77 (m_c, 1 H, 4-CH_2), 3.79 (dd, $J = 3.0$, 12.5 Hz, 1 H, 9-H), 3.90, 4.14 (AB system, $J_{AB} = 14.2$ Hz, 1 H each, NCH_2), 4.21 (d, $J = 8.5$ Hz, 1 H, 5-H), 4.24 (d, $J = 12.5$ Hz, 1 H, 9-H), 4.33 (ddd, $J = 3.2$, 7.2, 8.5 Hz, 1 H, 4-H), 4.68 (td, $J \approx 2.5$, 5.5 Hz, 1 H, 10-H), 7.26, 7.32, 7.37 (3 m_c, 1 H, 2 H, 2 H, Ph) ppm; ^{13}C NMR (125 MHz, $CDCl_3$): δ -4.4, -3.7 (2 q, SiMe), 18.1, 26.0 (s, q, $SiCMe_3$), 27.2, 31.2 (2 q, Me), 45.7 (d, C-1), 58.5 (t, NCH_2), 64.8 (t, 4-CH_2), 69.4 (t, C-9), 71.6 (d, C-6), 72.0 (d, C-5), 73.5, 74.1 (2 d, C-10, C-4), 77.7 (s, C-2), 127.3, 128.37, 128.43, 138.0 (3 d, s, Ph) ppm; IR (ATR) $\tilde{\nu}$: 3570, 3435 (OH), 3085-3030 (=C-H), 2950-2855 (C-H), 1250 (C-O), 1150, 1085-1035 (C-O-C) cm^{-1}; ESI-TOF (m/z): $[M + H]^+$ calcd for $C_{23}H_{40}NO_5Si$, 438.2670; found, 438.2677; Anal. calcd for $C_{23}H_{39}NO_5Si$ (437.6): C, 63.12; H, 8.98; N, 3.20; found: C, 62.80; H, 8.97; N, 3.14.

Reduction of 13: (1*S*,2*R*,4*R*,5*S*,6*R*,10*S*)-7-Benzyl-2-(*p*-bromophenyl)-5-(*tert*-butyldimethylsiloxy)-4-(hydroxymethyl)-3,8-dioxa-7-azabicyclo[4.3.1]decan-10-ol

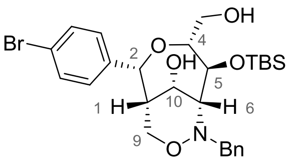

Following typical procedure 3, ketone **13** (165 mg, 0.293 mmol) and $NaBH_4$ (22 mg, 0.58 mmol) were reacted in dry EtOH (5 mL) for 40 min at 0 °C to rt to provide the corresponding secondary alcohol (165 mg, quant.) as a colorless solid; mp 174 °C; $[\alpha]_D^{22}$ +35.5 (*c* 0.60, $CHCl_3$); TLC [silica gel, hexanes/EtOAc 4:1] R_f 0.20; ^1H NMR (500 MHz, $CDCl_3$): δ 0.07, 0.15 (2 s, 3 H each, SiMe), 0.91 (s, 9 H, Si*t*-Bu), 2.02 (m_c, 1 H, 1-H), 2.30 (bs, 1 H, 4-OH), 2.91 (bs, 1 H, 10-OH), 3.45 (d, $J = 5.8$ Hz, 1 H, 6-H), 3.65 (dd, $J = 6.3$, 11.3 Hz, 1 H, 4-CH_2), 3.69, 3.76 (AB part of ABXY system, J_{AY} =

0.9 Hz, J_{AX} = 3.7 Hz, J_{AB} = 12.2 Hz, 1 H each, 9-H), 3.88 (dd, J = 2.8, 11.3 Hz, 1 H, 4-CH_2), 3.91 (A part of AB system, J_{AB} = 14.4 Hz, 1 H, NCH_2), 4.06 (ddd, J = 2.8, 6.3, 8.2 Hz, 1 H, 4-H), 4.19 (B part of AB system, J_{AB} = 14.4 Hz, 1 H, NCH_2), 4.43 (d, J = 8.2 Hz, 1 H, 5-H), 4.66 (t, J ≈ 5.8 Hz, 1 H, 10-H), 5.32 (m_c, 1 H, 2-H), 7.23 (AB part of AA'BB' system, J_{AB} = 8.5 Hz, 2 H, Ar), 7.26, 7.32, 7.37 (3 m_c, 1 H, 2 H, 2 H, Ph), 7.44 (A'B' part of AA'BB' system, $J_{A'B'}$ = 8.5 Hz, 2 H, Ar) ppm, *no BX coupling present; ^{13}C NMR (125 MHz, $CDCl_3$): δ -4.5, -3.7 (2 q, SiMe), 18.0, 25.9 (s, q, $SiCMe_3$), 44.1 (d, C-1), 58.9 (t, NCH_2), 64.9 (t, 4-CH_2), 67.0 (t, C-9), 70.0 (d, C-6), 70.8 (d, C-10), 71.5 (d, C-5), 80.0 (d, C-2), 84.2 (d, C-4), 120.8, 127.4 (s, d, Ar), 127.7, 128.1, 128.5 (3 d, Ph), 131.4 (d, Ar), 137.6, 140.5 (2 s, Ar, Ph) ppm; IR (ATR) $\tilde{\nu}$: 3435 (OH), 3085-3010 (=C-H), 2930-2860 (C-H), 1250 (C-O), 1070 (C-O-C) cm^{-1}; ESI-TOF (m/z): $[M + Na]^+$ calcd for $C_{27}H_{38}BrNO_5SiNa$, 586.1595; found, 586.1551; Anal. calcd for $C_{27}H_{38}BrNO_5Si$ (564.6): C, 57.44; H, 6.78; N, 2.48; found: C, 57.58; H, 6.81; N, 2.49.

Synthesis of (1*S*,4*R*,5*S*,6*R*,10*S*)-7-benzyl-4-(hydroxymethyl)-2,2-dimethyl-3,8-dioxa-7-azabicyclo[4.3.1]decan-5,10-diol (**15**):

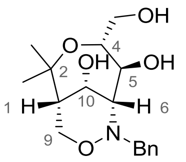

Following typical procedure 4, TBS-protected secondary alcohol of **12** (151 mg, 0.345 mmol) and TBAF (0.7 mL, 0.7 mmol) were reacted in THF (8.4 mL) for 3.5 h at 0 °C. The obtained crude material (yellow oil, 117 mg) was purified by column chromatography (silica gel, hexanes/EtOAc 1:3) to yield **15** (87 mg, 78%) as a colorless solid; mp 211–215 °C; $[\alpha]_D^{22}$ -12.7 (*c* 0.52, MeOH); TLC [silica gel, hexanes/EtOAc 1:3] R_f 0.15; ^1H NMR (700 MHz, CD_3OD): δ 1.28, 1.60 (2 s, 3 H

each, Me), 1.62 (dd, J = 3.0, 5.3 Hz, 1 H, 1-H), 3.29 (d, J ≈ 5.3 Hz, 1 H, 6-H), 3.52 (dd, J = 7.1, 11.3 Hz, 1 H, 4-CH_2), 3.75 (dd, J = 3.0, 12.6 Hz, 1 H, 9-H), 3.80 (dd, J = 3.3, 11.3 Hz, 1 H, 4-CH_2), 3.84 (d, J = 14.3 Hz, 1 H, NCH_2), 4.09-4.12 (m, 2 H, NCH_2, 5-H), 4.26 (d, J = 12.6 Hz, 1 H, 9-H), 4.47 (t, J ≈ 5.3 Hz, 1 H, 10-H), 4.55 (ddd, J = 3.3, 7.1, 10.0 Hz, 1 H, 4-H), 7.21, 7.28, 7.37 (3 m_c, 1 H, 2 H, 2 H, Ph) ppm; ^{13}C NMR (175 MHz, CD_3OD): δ 27.5, 31.2 (2 q, Me), 47.8 (d, C-1), 59.0 (t, C-9), 65.9 (t, 4-CH_2), 70.5 (d, C-5), 71.3 (t, NCH_2), 73.94, 73.92 (2 d, C-4, C-6)*, 74.8 (d, C-10), 77.9 (s, C-2), 127.9, 129.1, 129.7, 139.4 (3 d, s, Ph) ppm, *an unambiguous assignment of the signals was not possible; IR (ATR) $\tilde{\nu}$: 3445 (OH), 3085-3025 (=C-H), 2985-2850 (C-H), 1230 (C-O), 1170-1090 (C-O-C) cm^{-1}; ESI-TOF (m/z): $[M + Na]^+$ calcd for $C_{17}H_{25}NO_5Na$, 346.1625; found: 346.1615.

Synthesis of (1*S*,2*R*,4*R*,5*S*,6*R*,10*S*)-7-benzyl-2-(*p*-bromophenyl)-4-(hydroxymethyl)-3,8-dioxa-7-azabicyclo[4.3.1]decan-5,10-diol (**16**)**:**

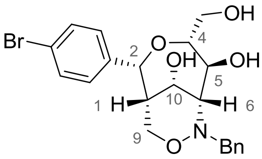

Following typical procedure 4, TBS-protected secondary alcohol of **13** (584 mg, 1.03 mmol) and TBAF (2.1 mL, 2.1 mmol) were reacted in THF (32 mL) for 40 min at 0 °C. The obtained crude material (colorless solid, 914 mg) was purified by column chromatography (silica gel, hexanes/EtOAc 1:2 to pure EtOAc) to yield **16** (351 mg, 75%) as a colorless solid; mp 259 °C; $[\alpha]_D^{22}$ +31.6 (*c* 0.60, MeOH); TLC [silica gel, hexanes/EtOAc 1:1] R_f 0.034; ^1H NMR (500 MHz, CD_3OD): δ 1.97 (m_c, 1 H, 1-H), 3.37 (d, J = 5.5 Hz, 1 H, 6-H), 3.64-3.66 (m, 2 H, 9-H), 3.71 (A part of ABX system, J_{AX} = 6.3 Hz, J_{AB} = 11.4 Hz, 1 H, 4-CH_2), 3.87 (A part of AB system, J_{AB} = 14.2 Hz, 1

H, NCH_2), 3.90 (B part of ABX system, J_{BX} = 3.0 Hz, J_{AB} = 11.4 Hz, 1 H, 4-CH_2), 4.09 (B part of AB system, J_{AB} = 14.2 Hz, 1 H, NCH_2), 4.22 (d, J = 9.5 Hz, 1 H, 5-H), 4.27 (ddd, J = 3.0, 6.3, 9.5 Hz, 1 H, 4-H), 4.49 (t, J ≈ 5.5 Hz, 1 H, 10-H), 5.26 (m_c, 1 H, 2-H), 7.19, 7.26 (2 m_c, 1 H, 2 H, Ph), 7.32 (AB part of AA'BB' systems, J_{AB} = 8.4 Hz, 2 H, Ar), 7.35 (m_c, 2 H, Ph), 7.43 (A'B' part of AA'BB' system, $J_{A'B'}$ = 8.4 Hz, 2 H, Ar) ppm; ^{13}C NMR (125 MHz, CD_3OD): δ 45.6 (d, C-1), 59.1 (t, NCH_2), 65.7 (t, 4-CH_2), 68.2 (t, C-9), 70.0 (d, C-5), 71.9 (d, C-10), 72.6 (d, C-6), 79.7 (d, C-2), 82.7 (d, C-4), 121.2 (s, Ar), 127.9 (d, Ph), 128.9 (d, Ar), 129.0, 129.6 (2 d, Ph), 131.9 (d, Ar), 139.0, 142.5 (2 s, Ph, Ar) ppm; IR (ATR) $\tilde{\nu}$: 3450 (OH), 3085-3025 (=C-H), 2955-2870 (C-H), 1225 (C-O), 1060 (C-O-C) cm^{-1}; ESI-TOF (m/z): $[M + H]^+$ calcd for $C_{12}H_{25}BrNO_5$, 450.0916; found: 450.0901; Anal. calcd for $C_{12}H_{24}BrNO_5$ (450.3): C, 56.01; H, 5.37; N, 3.11; found, C, 56.11; H, 5.44; N, 3.15.

Synthesis of (1*R*,4*S*,5*S*,6*S*)-7-benzyl-5-(*tert*-butyldimethylsiloxy)-2,2-dimethyl-4-[(prop-2'-inyloxy)methyl]-3,8-dioxa-7-azabicyclo[4.3.1]decan-10-one (**17**):

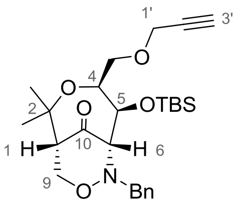

Alcohol **11** (300 mg, 0.689 mmol) was dissolved in CH_2Cl_2 (5 mL) and TBAI (93 mg, 0.25 mmol) was added. After cooling the reaction mixture to −20 °C, NaOH solution (9 N; 5.0 mL, 45 mmol) and propargyl bromide (0.5 mL, 5.6 mmol) were added and the solution was stirred for 7 d at rt. Water (5 mL) was added and the phases were separated. The aq layer was extracted with CH_2Cl_2 (3 × 10 mL). The combined organic layers were washed with brine, dried with Na_2SO_4, filtered through cotton and

the solvent was removed in vacuo. The obtained crude material (yellow oil, 300 mg) was purified by column chromatography (aluminum oxide, hexanes/EtOAc 10:1) to yield the alkine **17** (223 mg, 71%) as a colorless oil; $[\alpha]_D^{22}$ +15.9 (*c* 0.72, $CHCl_3$); TLC [silica gel, hexanes/EtOAc 3:1] R_f 0.60; ^{1}H NMR (500 MHz, $CDCl_3$): δ -0.07, -0.01 (2 s, 3 H each, SiMe), 0.87 (s, 9 H, Si*t*-Bu), 1.34, 1.35 (2 s, 3 H each, Me), 2.44 (t, *J* = 2.4 Hz, 1 H, 3'-H), 2.52 (m_c, 1 H, 1-H), 3.40 (dd, *J* = 1.2, 3.1 Hz, 1 H, 6-H), 3.43, 3.57 (AB part of ABX system, J_{AX} = 6.5 Hz, J_{BX} = 7.1 Hz, J_{AB} = 9.2 Hz, 1 H each, 4-CH_2), 3.94 (A part of AB system, J_{AB} = 13.7 Hz, 1 H, NCH_2), 4.10-4.21 (m, 5 H, NCH_2, 9-H, 1'-H), 4.24 (m_c, 1 H, 5-H), 4.66 (ddd, *J* = 1.2, 6.5, 7.1 Hz, 1 H, 4-H), 7.27-7.36 (m, 5 H, Ph) ppm; ^{13}C NMR (125 MHz, $CDCl_3$): δ -4.7, -4.6 (2 q, SiMe), 18.2 (s, Si*C*Me_3), 22.7 (q, Me), 25.9 (q, SiC*Me_3*), 31.7 (q, Me), 58.0 (d, C-1), 58.7* (t, NCH_2, C-1'), 68.2 (t, C-9), 68.3 (d, C-5), 70.1 (t, 4-CH_2), 73.2 (d, C-4), 74.5, 75.6 (2 s, C-2, C-2'), 75.7 (d, C-6), 79.9 (d, C-3'), 127.7, 128.6, 128.6, 136.6 (3 d, s, Ph), 200.2 (s, C-10) ppm, *overlapping signals; IR (ATR) $\tilde{\nu}$: 3305-3280 (≡C-H), 3085-3035 (=C-H), 2980-2860 (C-H), 2115 (C≡C), 1725 (C=O), 1605, 1585, 1495 (C=C), 1250 (C-O), 1130-1040 (C-O-C) cm^{-1}; ESI-TOF (m/z): $[M + H]^+$ calcd for $C_{26}H_{40}NO_5Si$, 474.2676; found, 474.2688; Anal. calcd for $C_{26}H_{39}NO_5Si$ (473.7): C: 65.93; H, 8.30; N, 2.96; found: C, 65.96; H, 8.34; N, 2.98.

Reduction of 17: (1*S*,4*S*,5*S*,6*R*,10*S*)-7-benzyl-5-(*tert*-butyldimethylsiloxy)-2,2-dimethyl-4-[(prop-2'-inyloxy)methyl]-3,8-dioxa-7-azabicyclo[4.3.1]decan-10-ol

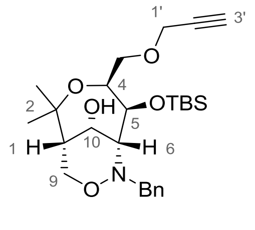

Following typical procedure 3, ketone **17** (56 mg, 0.12 mmol) and $NaBH_4$ (13 mg, 0.34 mmol) were reacted in dry EtOH (3 mL) for 4 h at 0 °C to rt to provide the corresponding secondary alcohol (56 mg, quant.) as colorless oil; $[\alpha]_D^{22}$ +37.6 (*c* 1.15, $CHCl_3$); TLC [silica gel, hexanes/EtOAc 3:1] R_f 0.46; ^1H NMR (500 MHz, $CDCl_3$): δ -0.05, 0.09 (2 s, 3 H each, SiMe), 0.91 (s, 9 H, Si*t*-Bu), 1.31, 1.50 (2 s, 3 H each, Me), 2.14 (m_c, 1 H, 1-H), 2.46 (t, J ≈ 2.4 Hz, 1 H, 3'-H), 3.33 (dd, J = 2.5, 5.6 Hz, 1 H, 6-H), 3.46, 3.61 (AB part of ABX system, J_{AX} = 6.4 Hz, J_{BX} = 7.7 Hz, J_{AB} = 9.4 Hz, 1 H each, 4-CH_2), 3.80 (A part of ABX system, J_{AX} = 3.0 Hz, J_{AB} = 12.7 Hz, 1 H, 9-H), 3.87 (d, J = 14.0 Hz, 1 H, NCH_2), 3.93 (B part of ABX system, J_{AB} = 12.7 Hz, 1 H, 9-H)[§], 3.99 (d, J = 11.1 Hz, 1 H, OH), 4.21* (d, J = 14.0 Hz, 1 H, NCH_2), 4.21* (m_c, 2 H, 1'-H), 4.41 (td, J ≈ 5.3, 11.1 Hz, 1 H, 10-H), 4.47 (m_c, 1 H, 5-H), 4.56 (ddd, J = 1.0, 6.4, 7.7 Hz, 1 H, 4-H), 7.27, 7.34, 7.38 (3 m_c, 1 H, 2 H, 2 H, Ph) ppm, *overlapping signals, [§]no BX coupling present. ^{13}C NMR (125 MHz, $CDCl_3$): δ -5.0, -4.5 (2 q, SiMe), 18.1 (s, Si*C*Me_3), 24.1 (q, Me), 25.9 (s, Si*C*Me_3), 34.4 (q, Me), 47.4 (d, C-1), 58.3 (t, NCH_2), 58.6 (t, C-1'), 64.4 (d, C-6), 68.8 (t, C-9), 70.0 (t, 4-CH_2), 70.1 (d, C-5), 73.1 (d, C-4), 73.3 (d, C-10), 74.6, 78.1 (2 s, C-2', C-2)*, 79.9 (d, C-3'), 127.4, 128.2, 128.5, 137.5 (3 d, s, Ph) ppm, *an unambiguous assignment of the signals was not possible; IR (ATR) $\tilde{\nu}$: 3520 (OH), 3285 (≡C-H), 3085-3030 (=C-H), 2950-2860 (C-H), 2115 (C≡C), 1605, 1495 (C=C), 1255, 1215 (C-O), 1100, 1070 (C-O-C) cm^{-1}; ESI-TOF (m/z): $[M + Na]^+$ calcd for $C_{26}H_{41}NO_5SiNa$, 498.2652; found, 498.2657; Anal. calcd for $C_{26}H_{41}NO_5Si$ (475.7): C, 65.63; H, 8.65; N, 2.92; found, C, 65.65; H, 8.69; N, 2.94.

Synthesis of (1*S*,4*S*,5*S*,6*R*,10*S*)-7-benzyl-2,2-dimethyl-4-[(prop-2'-inyloxy)-methyl]-3,8-dioxa-7-azabicyclo[4.3.1]decan-5,10-diol (**18**):

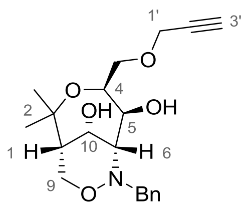

Following typical procedure 4, the above described TBS-protected alcohol (765 mg, 1.61 mmol) and TBAF (3.2 mL, 3.2 mmol) were reacted in THF (27 mL) for 3 h at 0 °C to rt. The obtained crude material (yellow oil, 957 mg) was purified by column chromatography (silica gel, hexanes/EtOAc 3:1 to 2:1) to yield **18** (512 mg, 88%) as a colorless solid; mp 75–78 °C; $[\alpha]_D^{22}$ -32.1 (*c* 0.56, $CHCl_3$); TLC [silica gel, hexanes/EtOAc 3:1] R_f 0.10; ^{1}H NMR (500 MHz, $CDCl_3$): δ 1.30, 1.52 (2 s, 3 H each, Me), 2.16 (dd, 2.5, 5.8 Hz, 1 H, 1-H), 2.48 (t, *J* = 2.4 Hz, 1 H, 3'-H), 3.28 (d, *J* = 4.4 Hz, 1 H, 4-OH), 3.39 (dd, *J* = 2.5, 4.9 Hz, 1 H, 6-H), 3.76-3.79 (m, 3 H, 4-CH_2, 9-H), 3.89 (A part of AB system, J_{AB} = 14.2 Hz, 1 H, NCH_2), 3.92 (d, *J* = 12.7 Hz, 1 H, 9-H), 4.06 (d, *J* = 10.1 Hz, 1 H, 10-OH), 4.10 (B part of AB system, J_{AB} = 14.2 Hz, 1 H, NCH_2), 4.21, 4.27 (AB part of ABX system, J_{AX} = J_{BX} = 2.4 Hz, J_{AB} = 15.9 Hz, 1 H each, 1'-H), 4.46 (td, *J* ≈ 5.3, 10.1 Hz, 1 H, 10-H), 4.55-4.56 (m, 2 H, 4-H, 5-H), 7.24-7.36 (m, 5 H, Ph) ppm; ^{13}C NMR (125 MHz, $CDCl_3$): δ 24.2, 34.5 (2 q, Me), 47.2 (d, C-1), 58.6 (t, NCH_2), 59.1 (t, C-1'), 64.7 (d, C-6), 69.0 (t, C-9), 70.7 (d, C-4 oder C-5), 72.2 (t, 4-CH_2), 72.8 (d, C-5 oder C-4), 73.8 (d, C-10), 75.0 (s, C-2'), 78.4 (s, C-2), 79.6 (d, C-3'), 127.4, 128.2, 128.2, 128.5, 137.6 (3 d, s, Ph) ppm; IR (ATR) $\tilde{\nu}$: 3485 (OH), 3305 (≡C-H), 3015 (=C-H), 2980-2875 (C-H), 2115 (C≡C), 1215 (C-O), 1135-1070 (C-O-C) cm^{-1}; ESI-TOF (m/z): $[M + Na]^+$ calcd for $C_{20}H_{27}NO_5Na$, 348.1781;

found, 348.1760; Anal. calcd for $C_{20}H_{27}NO_5$ (461.4): C, 66.46; H, 7.53; N, 3.88; found, C, 66.49; H, 7.57; N, 3.88.

Synthesis of (1*R*,4*S*,5*S*,6*S*)-7-{benzyl-5-(*tert*-butyldimethylsiloxy)-2,2-dimethyl-10-oxo-3,8-dioxa-7-azabicyclo[4.3.1]decan-4-yl}methylmethanesulfonate (19):

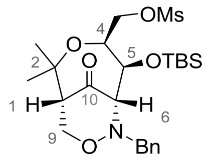

Alcohol **11** (100 mg, 0.23 mmol) was dissolved in dry CH_2Cl_2 (3.4 mL) under argon atmosphere. The yellow solution was cooled to 0 °C and NEt_3 (0.16 mL, 1.14 mmol) as well as MsCl (37 µL, 0.48 mmol) were added. After stirring for 5 h at rt, the reaction mixture was quenched with a solution of aq $NaHCO_3$ (10 mL), diluted with water (5 mL). The aq layer was extracted with CH_2Cl_2 (3 × 10 mL). The combined organic layers were washed with brine, dried with Na_2SO_4, filtered through cotton and the solvent was removed in vacuo. The obtained crude material (yellow oil, 189 mg) was purified by column chromatography (silica gel, hexanes/EtOAc 4:1) to yield mesylate **19** (117 mg, quant.) as a colorless oil; $[\alpha]_D^{22}$ +22.7 (*c* 0.75, $CHCl_3$); TLC [silica gel, hexanes/EtOAc 4:1] R_f 0.20; ^1H NMR (700 MHz, $CDCl_3$): δ -0.10, -0.02 (2 s, 3 H each, SiMe), 0.85 (s, 9 H, Si*t*-Bu), 1.34, 1.37 (2 s, 3 H each, Me), 2.55 (m_c, 1 H, 1-H), 3.01 (s, 3 H, $MeSO_2$), 3.37 (m_c, 1 H, 6-H), 3.96 (d, *J* = 13.4 Hz, 1 H, NCH_2), 4.15-4.19 (m, 6 H, 9-H, NCH_2, 4-CH_2, 5-H), 4.78 (ddt, *J* = 0.9, 5.7, 6.4 Hz, 1 H, 4-H), 7.27-7.28 (m, 5 H, Ph) ppm; ^{13}C NMR (175 MHz, $CDCl_3$): δ -4.9, -4.5 (2 q, SiMe), 18.1 (s, Si*C*Me_3), 22.8 (q, Me), 25.7 (q, SiC*Me₃*), 31.5 (q, Me), 37.5 (q, $MeSO_2$), 57.8 (d, C-1), 58.5 (t, NCH_2), 67.9* (t, C-9 or 4-CH_2), 68.5 (d, C-5), 69.8* (t, C-9 or 4-CH_2), 72.7 (d, C-4), 74.9 (s, C-2), 75.0 (d, C-6), 127.9, 128.66, 128.68, 136.1 (3 d, s, Ph),

199.6 (s, C-10) ppm, *an unambiguous assignment of the signals was not possible; IR (ATR) $\tilde{\nu}$: 3090-3035 (=C-H), 2975-2860 (C-H), 1725 (C=O), 1600, 1580, 1495 (C=C), 1255 (C-O), 1350 (SO_2), 1175, 1075 (C-O-C) cm^{-1}; ESI-TOF (m/z): $[M + Na]^+$ calcd for $C_{24}H_{39}NO_7SSiNa$, 536.2114; found, 536.2139.

Reduction of 19: (1*S*,4*S*,5*S*,6*R*,10*S*)-7-{benzyl-5-(*tert*-butyldimethylsiloxy)-10-hydroxy-2,2-dimethyl-3,8-dioxa-7-azabicyclo[4.3.1]decan-4-yl}methylmethane-sulfonate

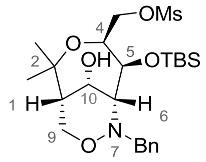

Following typical procedure 3, ketone **19** (92 mg, 0.179 mmol) and $NaBH_4$ (12 mg, 0.32 mmol) were reacted in dry EtOH (3 mL) for 6 h at 0 °C to provide the corresponding secondary alcohol (89 mg, 96%) as a colorless oil; $[\alpha]_D^{22}$ -36.3 (*c* 1.09, $CHCl_3$); TLC [silica gel, hexanes/EtOAc 3:1] R_f 0.22; ^1H NMR (500 MHz, $CDCl_3$): δ -0.10, 0.05 (2 s, 3 H each, SiMe), 0.89 (s, 9 H, Si*t*-Bu), 1.31, 1.49 (2 s, 3 H each, Me), 2.16 (m_c, 1 H, 1-H), 3.04 (s, 3 H, $MeSO_2$), 3.30 (dd, *J* = 2.6, 5.0 Hz, 1 H, 6-H), 3.81 (A part of ABX system, J_{AX} = 3.2 Hz, J_{AB} = 12.7 Hz, 1 H, 9-H), 3.81 (d, *J* = 11.4 Hz, 1 H, OH), 3.86 (d, *J* = 13.8 Hz, 1 H, NCH_2), 3.92 (B part of ABX system, J_{AB} = 12.7 Hz, 1 H, 9-H)*, 4.17 (A part of ABX system, J_{AX} = 5.5 Hz, J_{AB} = 10.3 Hz, 1 H, 4-CH_2), 4.19 (d, *J* = 13.8 Hz, 1 H, NCH_2), 4.21 (B part of ABX system, J_{BX} = 7.5 Hz, J_{AB} = 10.3 Hz, 1 H, 4-CH_2), 4.38 (dd, *J* = 1.2, 2.6 Hz, 1 H, 5-H), 4.41 (dd, *J* = 5.0, 11.4 Hz, 1 H, 10-H), 4.66 (ddd, *J* = 1.2, 5.5, 7.5 Hz, 1 H, 4-H), 7.27-7.34 (m, 5 H, Ph) ppm, *No BX coupling present; ^{13}C NMR (125 MHz, $CDCl_3$): δ -5.0, -4.3 (2 q, SiMe), 18.1 (s, Si*C*Me_3), 24.2 (q, Me), 25.9 (q, SiC*Me*$_3$), 31.0 (d, C-1), 34.2 (q, Me), 37.6 (q,

$MeSO_2$), 58.3 (t, NCH_2), 63.9 (d, C-6), 68.5 (t, C-9), 69.9 (t, 4-CH_2), 70.6 (d, C-5), 72.7 (d, C-4), 72.8 (d, C-10), 78.7 (s, C-2), 127.7, 128.3, 128.6, 137.0 (3 d, s, Ph) ppm; IR (ATR) $\tilde{\nu}$: 3530 (OH), 3085-3030 (=C-H), 2970-2860 (C-H), 1495 (C=C), 1255 (C-O), 1355 (SO_2), 1175, 1090-1070 (C-O-C) cm^{-1}; ESI-TOF (m/z): $[M + Na]^+$ calcd for $C_{24}H_{41}NO_7SSiNa$, 538.2265; found, 538.2271; Anal. calcd for $C_{24}H_{41}NO_7SSi$ (515.7): C, 55.83; H, 8.28; N, 2.61; S, 6.33; found, C, 55.89; H, 8.01; N, 2.72; S, 6.22.

Synthesis of TBS-protected tricycles: (1*S*,4*S*,5*S*,6*R*,10*S*)-7-benzyl-5-(*tert*-butyldimethylsiloxy)-2,2-dimethyl-3,8,11-trioxa-7-azatricyclo[4.3.1.2^{4,10}]-dodecane

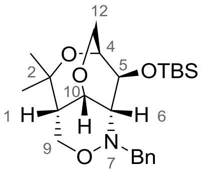

The aforementioned mesylate (596 mg, 1.16 mmol) was dissolved in dry DMF (48 mL) under argon atmosphere and NEt_3 (0.64 mL, 4.74 mmol) was added. The slightly yellow mixture was heated to reflux overnight. After removing the solvent in vacuo, EtOAc (30 mL) and water (5 mL) were added and the phases were separated. The aq layer was extracted with EtOAc (3 × 20 mL). The combined organic layers were washed with brine, dried with Na_2SO_4, filtered through cotton and the solvent was removed in vacuo. The obtained material (brown oil, 558 mg) was purified by column chromatography (silica gel, hexanes/EtOAc 7:1) to yield tricyclic compound (445 mg, 91%) as a slightly yellow solid; mp 90–93 °C; $[\alpha]_D^{22}$ -19.1 (*c* 0.80, $CHCl_3$); TLC [silica gel, hexanes/EtOAc 6:1] R_f 0.47; ^1H NMR (500 MHz, $CDCl_3$): δ 0.14, 0.22 (2 s, 3 H each, SiMe), 0.89 (s, 9 H, Si*t*-Bu), 1.28, 1.57 (2 s, 3 H each, Me), 1.94 (dd, *J* = 3.3, 7.1 Hz, 1 H, 1-H), 3.35 (dd, *J* = 3.7, 5.5 Hz, 1 H, 6-H), 3.73 (dd, *J* = 3.3, 12.9 Hz, 1 H,

9-H), 3.78 (m_c,1 H, 5-H), 3.83, 4.06 (AB system, J_{AB} = 14.8 Hz, 1 H each, NCH_2), 4.12 (A part of ABX system, J_{AX} = 1.8 Hz, J_{AB} = 11.4 Hz, 1 H, 12-H), 4.17* (d, $J \approx$ 12.9 Hz, 1 H, 9-H), 4.18* (B part of ABX system, J_{BX} = 1.9 Hz, J_{AB} = 11.4 Hz, 1 H, 12-H), 4.44 (dd, J = 5.5, 7.1 Hz, 1 H, 10-H), 4.46 (m_c, 1 H, 4-H), 7.26, 7.33, 7.37 (3 m_c, 1 H, 2 H, 2 H, Ph) ppm, *overlapping signals. ^{13}C NMR (125 MHz, $CDCl_3$): δ -4.1, -3.6 (2 q, SiMe), 17.9, 25.9 (s, q, $SiCMe_3$), 30.9, 33.8 (2 q, Me), 46.3 (d, C-1), 58.3 (t, NCH_2), 61.4 (t, C-12), 68.1 (d, C-4), 68.9 (d, C-6), 69.2 (t, C-9), 71.3 (d, C-10), 75.7 (d, C-5), 75.6 (s, C-2), 127.2, 128.1, 128.4, 138.2 (3 d, s, Ph) ppm; IR (ATR) $\tilde{\nu}$: 3100-3030 (=C-H), 2990-2855 (C-H), 1600, 1495 (C=C), 1255, 1230 (C-O), 1060, 1073 (C-O-C) cm^{-1}; ESI-TOF (m/z): $[M + H]^+$ calcd for $C_{23}H_{38}NO_4Si$, 420.2565; found, 420.2603; Anal. calcd for $C_{23}H_{37}NO_4Si$ (419.6): C, 65.83; H, 8.89; N, 3.34; found, C, 66.42; H, 8.98; N, 3.41.

Synthesis of (1*S*,4*S*,5*S*,6*R*,10*S*)-7-benzyl-2,2-dimethyl-3,8,11-trioxa-7-aza-tricyclo[4.3.1.2^{4,10}]dodecan-5-ol (**20**):

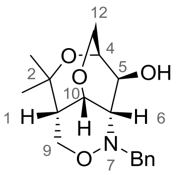

Following typical procedure 4, the above described tricyclic compound (425 mg, 1.01 mmol) and TBAF (2 mL, 2.0 mmol) were reacted in THF (21 mL) for 3.5 h at 0 °C to rt. The obtained crude material (yellow oil, 500 mg) was purified by column chromatography (silica gel, hexanes/EtOAc 3:1 to 1:1) to yield **20** (267 mg, 86%) as a colorless solid; mp 178–180 °C; $[\alpha]_D^{22}$ -5.99 (*c* 0.63, MeOH); TLC [silica gel, hexanes/EtOAc 2:1] R_f 0.14; ^1H NMR (500 MHz, benzene-d_6): δ 1.16 (s, 3 H, Me), 1.46 (dd, J = 3.6, 7.0 Hz, 1 H, 1-H), 1.47 (s, 3 H, Me), 3.16 (m_c, 1 H, 6-H), 3.44 (q, $J \approx$

2.0 Hz, 1 H, 4-H), 3.52 (dd, J = 3.6, 12.9 Hz, 1 H, 9-H), 3.81 (d, J = 13.0 Hz, 1 H, NCH_2), 3.87 (d, J = 12.9 Hz, 1 H, 9-H), 4.02 (m_c, 2 H, 12-H), 4.12 (d, J = 14.0 Hz, 1 H, NCH_2), 4.33* (dd, J = 5.6, 7.0 Hz, 1 H, 10-H), 4.33* (m_c, 1 H, 5-H), 7.13, 7.24, 7.48 (3 m_c, 1 H, 2 H, 2 H, Ph) ppm, *overlapping signals, the OH-Signal could not be detected; ^{13}C NMR (500 MHz, benzene-d_6): δ 30.7, 33.9 (2 q, Me), 46.4 (d, C-1), 58.3 (t, NCH_2), 61.5 (t, C-12), 66.5 (d, C-6), 67.2 (d, C-5), 69.2 (t, C-9), 71.3 (d, C-10), 76.5 (s, C-2), 76.4 (d, C-4), 127.4, 128.3, 128.5, 129.4 (3 d, s, Ph) ppm; IR (ATR) $\tilde{\nu}$: 3415 (OH), 3090 (=C-H), 2990-2855 (C-H), 1495 (C=C), 1255, 1235, 1215 (C-O), 1150-1120, 1090-1050 (C-O-C) cm^{-1}; ESI-TOF (m/z): $[M + H]^+$ calcd for $C_{17}H_{24}NO_4$, 306.1700; found, 306.1710; Anal. calcd for $C_{17}H_{23}NO_4$ (305.4): C, 66.86; H, 7.59; N, 4.59; found, C, 66.83; H, 7.49; N, 4.52.

Synthesis of (1*S*,4*S*,5*S*,6*R*,10*S*)-4-(azidomethyl)-7-benzyl-5-(*tert*-butyldimethylsiloxy)-2,2-dimethyl-3,8-dioxa-7-azabicyclo[4.3.1]decan-10-ol (**21**):

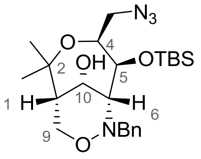

Following typical procedure 3, ketone **23** (60 mg, 0.13 mmol) and $NaBH_4$ (10 mg, 0.26 mmol) were reacted in dry EtOH (2 mL) overnight at 0 °C to rt to provide the corresponding secondary alcohol **21** (60 mg, quant.) as a colorless solid; mp 98–101 °C; $[\alpha]_D^{22}$ +29.2 (*c* 0.87, $CHCl_3$); TLC [silica gel, hexanes/EtOAc 6:1] R_f 0.41; ^{1}H NMR (700 MHz, $CDCl_3$): δ -0.09, 0.04 (2 s, 3 H each, SiMe), 0.90 (s, 9 H, Si*t*-Bu), 1.37, 1.53 (2 s, 3 H each, Me), 2.16 (dd, J = 3.0, 5.4 Hz, 1 H, 1-H), 3.07 (dd, J = 4.5, 12.2 Hz, 1 H, 4-CH_2), 3.28 (dd, J = 2.5, 5.4 Hz, 1 H, 6-H), 3.49 (dd, J = 8.6, 12.2 Hz, 1 H, 4-CH_2), 3.82 (A part of ABX system, J_{AX} = 3.0 Hz, J_{AB} = 12.8 Hz, 1 H, 9-H), 3.83 (d, J

= 11.0 Hz, 1 H, OH), 3.86 (d, *J* = 13.8 Hz, 1 H, NCH_2), 3.94 (B part of ABX system, J_{AB} = 12.8 Hz, 1 H, 9-H)*, 4.16 (d, *J* = 13.8 Hz, 1 H, NCH_2), 4.27 (m_c, 1 H, 5-H), 4.41 (td, *J* ≈ 5.4, 11.0 Hz, 1 H, 10-H), 4.48 (ddd, *J* = 1.0, 4.5, 8.6 Hz, 1 H, 4-H), 7.26-7.36 (m, 5 H, Ph) ppm, *no BX coupling present; ^{13}C NMR (175 MHz, $CDCl_3$): δ -4.9, -4.4 (2 q, SiMe), 18.2 (s, Si*C*Me$_3$), 23.8 (q, Me), 25.9 (q, SiC*Me$_3$*), 34.4 (q, Me), 47.6 (d, C-1), 53.4 (t, 4-CH_2), 58.4 (t, NCH_2), 64.3 (d, C-6), 68.6 (t, C-9), 71.8 (d, C-5), 72.7 (d, C-10) 74.3 (d, C-4), 78.7 (s, C-2), 127.7, 128.3, 128.7, 137.2 (3 d, s, Ph) ppm; IR (ATR) $\tilde{\nu}$: 3525 (OH), 3085-3030 (=C-H), 2990-2855 (C-H), 2100 (N_3), 1495 (C=C), 1250 (C-O), 1070 (C-O-C) cm^{-1}; ESI-TOF (m/z): $[M + H]^+$ calcd for $C_{23}H_{39}N_4O_4Si$, 463.2735; found, 463.2779; Anal. calcd for $C_{23}H_{38}N_4O_4Si$ (462.7): C, 59.71; H, 8.28; N, 12.11; found, C, 59.50; H, 8.70; N, 11.31.

Synthesis of (1*S*,4*S*,5*S*,6*R*,10*S*)-4-(azidomethyl)-7-benzyl-2,2-dimethyl-3,8-dioxa-7-azabicyclo[4.3.1]decan-5,10-diol (**24**)**:**

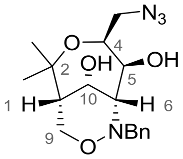

Following typical procedure 4, the above described TBS-protected alcohol (61 mg, 0.13 mmol) and TBAF (0.3 mL, 0.3 mmol) were reacted in THF (3.4 mL) for 1 h at 0 °C to rt. The obtained crude material (yellow oil, 40 mg) was purified by preparative TLC (silica gel, hexanes/EtOAc 2:1) to yield **24** (30 mg, 65%) as colorless solid; mp 75–79 °C; $[\alpha]_D^{22}$ +35.1 (*c* 0.63, $CHCl_3$); TLC [silica gel, hexanes/EtOAc 2:1] R_f 0.36; ^{1}H NMR (500 MHz, $CDCl_3$): δ 1.39, 1.54 (2 s, 3 H each, Me), 2.18 (dd, *J* = 2.5, 5.9 Hz, 1 H, 1-H), 2.97 (d, *J* = 7.1 Hz, 1 H, 5-OH), 3.24 (dd, *J* = 4.2, 12.6 Hz, 1 H, 4-CH_2), 3.36 (dd, *J* = 2.5, 5.0 Hz, 1 H, 6-H), 3.61 (d, *J* = 9.4 Hz, 1 H, 10-OH), 3.64 (dd, *J* =

8.4, 12.6 Hz, 1 H, 4-CH_2), 3.77 (A part of ABX system, J_{AX} = 3.0 Hz, J_{AB} = 12.8 Hz, 1 H, 9-H), 3.86 (A part of AB system, J_{AB} = 14.0 Hz, 1 H, NCH_2), 3.97 (B part of ABX system, J_{AB} = 12.8 Hz, 1 H, 9-H)*, 4.09 (B part of AB system, J_{AB} = 14.0 Hz, 1 H, NCH_2), 4.28 (d, J ≈ 4.4 Hz, 1 H, 5-H), 4.48-4.52 (m, 2 H, -H, 10-H), 7.25-7.34 (m, 5 H, Ph) ppm, *no BX coupling present; ^{13}C NMR (125 MHz, $CDCl_3$): δ 23.9, 34.6 (2 q, Me), 47.2 (d, C-1), 53.2 (t, 4-CH_2), 58.6 (t, NCH_2), 65.3 (d, C-6), 69.2 (t, C-9), 70.3 (d, C-5), 73.8, 74.5 (2 d, C-10, C-4)*, 78.7 (s, C-2), 127.6, 128.3, 128.6, 137.2 (3 d, s, Ph) ppm, *an unambiguous assignment of the signals was not possible; IR (ATR) $\tilde{\nu}$: 3380 (OH), 3085-3030 (=C-H), 2975-2870 (C-H), 2105 (N_3), 1495 (C=C), 1290, 1275, 1215 (C-O), 1150-1170 (C-O-C) cm^{-1}; ESI-TOF (m/z): $[M + H]^+$ calcd for $C_{17}H_{25}N_4O_4$, 349.1876; found, 349.1886; Anal. calcd for $C_{17}H_{24}N_4O_4$ (348.4): C, 58.61; H, 6.94; N, 16.08; found, C, 58.61; H, 6.93; N, 16.15.

TBS protection of 24: (1*S*,4*S*,5*S*,6*R*,10*S*)-4-(azidomethyl)-7-benzyl-5,10-bis(*tert*-butyldimethylsiloxy)-2,2-dimethyl-3,8-dioxa-7-azabicyclo[4.3.1]decane

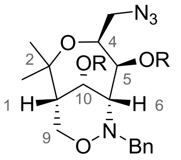

R = TBS

Diol **24** (311 mg, 0.893 mmol) was dissolved in dry CH_2Cl_2 (10 mL) and the solution was cooled to 0 °C under argon atmosphere. NEt_3 (0.5 mL, 3.6 mmol) and TBSOTf (0.62 mL, 2.7 mmol) were added and the reaction mixture was stirred at rt for 3.5 h. Water was added (5 mL) and the phases were separated. The aq layer was extracted with CH_2Cl_2 (3 x 15 mL). The combined organic layers were washed with brine, dried with Na_2SO_4, filtered through cotton and the solvent was removed in vacuo. The obtained crude material (orange oil, 535 mg) was purified by column chromatography

(silica gel, hexanes/EtOAc 50:1) to yield the TBS-protected azide (473 mg, 92%) as a colorless solid; mp 85–87 °C; $[\alpha]_D^{22}$ +20.2 (*c* 0.43, $CHCl_3$); TLC [silica gel, hexanes/EtOAc 10:1] R_f 0.42; ^{1}H NMR (500 MHz, $CDCl_3$): δ -0.11, 0.06 (2 s, 3 H each, SiMe), 0.12 (s, 6 H, 2 SiMe), 0.91, 0.96 (2 s, 9 H each, Si*t*-Bu), 1.32, 1.52 (2 s, 3 H each, Me), 1.98 (m_c, 1 H, 1-H), 3.01 (dd, *J* = 3.5, 12.2 Hz, 1 H, 4-CH_2), 3.07 (dd, *J* = 1.3, 4.2 Hz, 1 H, 6-H), 3.56 (dd, *J* = 9.0, 12.2 Hz, 1 H, 4-CH_2), 3.81 (A part of ABX system, J_{AX} = 3.6 Hz, J_{AB} = 12.6 Hz, 1 H, 9-H), 3.82 (d, *J* = 14.2 Hz, 1 H, NCH_2), 3.97 (B part of ABX system, J_{AB} = 12.6 Hz, 1 H, 9-H)*, 4.16 (d, *J* = 14.2 Hz, 1 H, NCH_2), 4.27 (m_c, 1 H, 5-H), 4.31 (ddd, *J* = 1.8, 3.5, 9.0 Hz, 1 H, 4-H), 4.58 (t, *J* ≈ 4.2 Hz, 1 H, 10-H), 7.25-7.37 (m, 5 H, Ph) ppm, *no BX coupling present; ^{13}C NMR (125 MHz, $CDCl_3$): δ -4.8, -4.5, -4.4, -4.3 (4 q, SiMe), 18.6, 18.9 (2 s, Si*C*Me_3), 24.2 (q, Me), 26.5, 26.9 (2 q, SiC*Me₃*), 34.4 (q, Me), 48.6 (d, C-1), 53.9 (t, 4-CH_2), 58.6 (t, NCH_2), 67.9 (d, C-6), 69.6 (t, C-9), 71.9 (d, C-5), 74.7 (d, C-10), 75.3 (d, C-4), 127.5, 128.1, 128.6, 137.7 (3 d, s, Ph) ppm, the signal for C-2 could not be detected; IR (ATR) $\tilde{\nu}$: 3085-3035 (=C-H), 2950-2860 (C-H), 2095 (N_3), 1605, 1495 (C=C), 1255 (C-O), 1150, 1075 (C-O-C) cm^{-1}; ESI-TOF (m/z): $[M + Na]^+$ calcd for $C_{29}H_{52}N_4O_4Si_2Na$, 599.3425; found, 599.3482; Anal. calcd for $C_{29}H_{52}N_4O_4Si_2$ (576.9): C, 60.37; H, 9.08; N, 9.71; found, C, 60.57; H, 8.97; N, 9.85.

Synthesis of (1*S*,4*S*,5*S*,6*R*,10*S*)-{7-benzyl-5,10-bis(*tert*-butyldimethylsiloxy)-2,2-dimethyl-3,8-dioxa-7-azabicyclo[4.3.1]decan-4-yl)}methanamine (25):

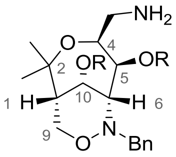

TPP (1.08 g, 4.12 mmol) was added to a solution of the above described TBS-protected azide (473 mg, 0.820 mmol) in THF (36 mL) and water (12 mL). After stirring for 1 h at rt additional TPP (930 mg, 3.55 mmol, 4.37) was added. The reaction mixture was stirred overnight and the solvent was removed in vacuo. The obtained crude material (colorless solid, 450 mg) was purified by column chromatography (silica gel, CH_2Cl_2 to CH_2Cl_2/7 N NH_3 in MeOH 30:1) to yield amine **25** (393 mg, 87%) as a colorless oil; $[\alpha]_D^{22}$ -7.71 (*c* 12.3, $CHCl_3$); TLC [silica gel, CH_2Cl_2/7 N NH_3 in MeOH 10:1] R_f 0.30; ^{1}H NMR (500 MHz, $CDCl_3$): δ -0.11, 0.05 (2 s, 3 H each, SiMe), 0.11 (s, 6 H, SiMe), 0.88, 0.95 (2 s, 9 H each, Si*t*-Bu), 1.24, 1.53 (2 s, 3 H each, Me), 1.97 (m_c, 1 H, 1-H), 2.63 (dd, J = 1.9, 12.5 Hz, 1 H, 4-CH_2), 2.93 (dd, J = 9.9, 12.5 Hz, 1 H, 4-CH_2), 3.07 (m_c, 1 H, 6-H), 3.78* (A part of ABX system, J_{AX} = 3.1 Hz, J_{AB} = 12.6 Hz, 1 H, 9-H), 3.86* (d, J = 14.1 Hz, 1 H, NCH_2), 3.92 (B part of ABX system, J_{AB} = 12.6 Hz, 1 H, 9-H)[§], 4.04 (d, J ≈ 9.9 Hz, 1 H, 4-H), 4.15 (d, J = 14.1 Hz, 1 H, NCH_2), 4.28 (m_c, 1 H, 5-H), 4.75 (t, J ≈ 4.8 H, 1 H, 10-H), 7.23, 7.31, 7.34 (3 m_c, 1 H, 2 H, 2 H, Ph) ppm, the NH_2 signal could not be detected, *overlapping signals, [§]no BX coupling present; ^{13}C NMR (125 MHz, $CDCl_3$): δ -4.7, -4.6, -4.4, -3.2 (4 q, SiMe), 18.4, 18.8 (2 s, Si*C*Me_3), 25.5 (q, Me), 26.5, 26.8 (2 q, SiC*Me_3*), 34.5 (q, Me), 44.9 (t, 4-CH_2), 48.4 (d, C-1), 58.5 (t, NCH_2), 68.1 (d, C-6), 69.4 (t, C-9), 72.3 (d, C-5), 74.6 (d, C-10), 76.6 (s, C-2), 78.2 (d, C-4), 127.3, 128.0, 128.4, 137.7 (3 d, s, Ph) ppm; IR (ATR) $\tilde{\nu}$: 3380 (NH_2), 3100-3030 (=C-H), 2955-

2855 (C-H), 1605, 1585, 1495 (C=C), 1255 (C-O), 1070 (C-O-C) cm^{-1}; ESI-TOF (m/z): $[M + H]^+$ calcd for $C_{29}H_{55}N_2O_4Si_2$, 551.3695; found, 551.3715.

Typical procedure for hydrogenolysis with Pd/C in MeOH (procedure 6)

(2*S*,2a*S*,5a*S*,6*S*,7*S*)-2,7-Bis(hydroxymethyl)-8,8-dimethyl-octahydrooxepino-[4,5,*d*][1,3]oxazol-6-ol (29):

A suspension of Pd/C (180 mg, 0.169 mmol Pd) in dry MeOH (5.5 mL) was saturated with hydrogen at rt for 1 h. Then the bicyclic compound **14** (147 mg, 0.455 mmol, dissolved in 2 mL dry MeOH) was added. The reaction was stirred under hydrogen atmosphere (balloon) for 17 h. Afterwards, the mixture was filtered through Celite®, washed with MeOH and the solvent was removed in vacuo. The obtained crude material (colorless oil, 102 mg; product **26**/side product **29** = 67:33) was purified by column chromatography (silica gel, MeCN/aq NH_3 (25%) 8:1 to 5:1) to yield the amine **26** (67 mg, 63%) as a colorless solid and small amounts (10 mg, ca. 90% pure) of the side product **29** as a colorless oil were isolated.

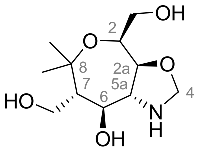

colorless oil; $[\alpha]_D^{22}$ -4.26 (*c* 0.11, MeOH); TLC [silica gel, MeCN/aq NH_3 (25%) 5:1] R_f 0.24; ^1H NMR (700 MHz, CD_3OD): δ 1.22, 1.35 (2 s, 3 H each, Me), 1.81 (dt, *J* = 5.2, 10.5 Hz, 1 H, 7-H), 3.09 (dd, *J* = 7.5, 9.0 Hz, 1 H, 5a-H), 3.54 (dd, *J* = 7.2, 11.6 Hz, 1 H, 2-CH_2), 3.58-3.65 (m, 3 H, 2-CH_2, 2a-H), 3.73-3.76 (m, 3 H, 2-H, 6-H, 7-CH_2), 4.43, 4.54 (2 d, *J* = 5.7 Hz, 1 H each, 5-H) ppm; ^{13}C NMR (175 MHz, CD_3OD): δ

21.0, 31.8 (2 q, Me), 58.2 (d, C-7), 63.4 (t, 7-CH_2), 63.6 (t, 2-CH_2), 71.4 (d, C-5a), 73.6, 75.9 (2 d, C-2, C-6)*, 78.1, 78.3 (s, d, C-2a, C-8)*, 81.9 (t, C-4) ppm, *an unambiguous assignment of the signals was not possible; IR (ATR) $\tilde{\nu}$: 3375 (OH, NH), 2980-2830 (C-H), 1210 (C-O), 1185, 1020 (C-O-C) cm^{-1}; ESI-TOF (m/z): $[M + Na]^+$ calcd for $C_{11}H_{21}NO_5Na$, 270.1312; found, 270.1317.

Synthesis of (2*R*,3*S*,4*R*,5*S*,6*S*)-4-amino-2,6-bis(hydroxymethyl)-7,7-dimethyloxepan-3,5-diol (**27**)**:**

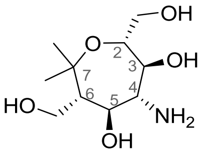

Following typical procedure 6, the bicyclic compound **15** (100 mg, 0.309 mmol), Pd/C (100 mg, 0.094 mmol Pd) were reacted in dry MeOH (27 mL) for 3 d at rt under hydrogen atmosphere. The obtained crude material (yellow oil, 85 mg; product **27**/side product **30** = 83:17) was purified by column chromatography (silica gel, MeCN/aq NH_3 (25%) 8:1 to 5:1) to yield a mixture of the amine **27** and side product **30** (64 mg, 88%, **27**/**30** = 94:6) as a colorless solid; mp 159–161 °C; $[\alpha]_D^{22}$ +29.5 (*c* 0.54, MeOH); TLC [silica gel, CH_2Cl_2/aq NH_3 5:1] R_f 0.07; ^1H NMR (700 MHz, CD_3OD): δ 1.06, 1.30 (2 s, 3 H each, Me), 2.00 (td, $J \approx 3.0, 8.7$ Hz, 1 H, 6-H), 2.70 (t, $J \approx 9.4$ Hz, 1 H, 4-H), 3.20 (dd, $J = 8.9, 9.7$ Hz, 1 H, 3-H), 3.50-3.53 (m, 2 H, 2-CH_2, 6-CH_2), 3.57 (ddd, $J = 2.7, 6.4, 9.7$ Hz, 1 H, 2-H), 3.72 (t, $J \approx 9.4$ Hz, 1 H, 5-H), 3.77 (dd, $J = 2.7, 11.2$ Hz, 1 H, 2-CH_2), 3.79 (dd, $J = 8.7, 11.1$ Hz, 1 H, 6-CH_2) ppm; ^{13}C NMR (175 MHz, CD_3OD): δ 24.0, 25.9 (2 q, Me), 54.2 (d, C-6), 63.0 (t, 6-CH_2), 64.5 (t, 2-CH_2), 66.7 (d, C-3), 70.8 (d, C-2), 72.9 (d, C-5), 74.2 (d, C-4), 76.2 (s, C-7) ppm; IR (ATR) $\tilde{\nu}$: 3345-3290 (OH, NH_2), 2975-2925 (C-H) cm^{-1}; ESI-TOF (m/z): $[M + H]^+$

calcd for $C_{10}H_{22}NO_5$, 236.1420; found, 236.1497; Anal. calcd for $C_{10}H_{21}NO_5$ (235.3): C, 47.42; H, 9.15; N, 5.53; found, C, 47.78; H, 8.63; N, 5.05.

(2*R*,2a*S*,5a*S*,6*S*,7*S*)-2,7-Bis(hydroxymethyl)-8,8-dimethyl-octahydrooxepino-[4,5,*d*][1,3]oxazol-6-ol (**30**):

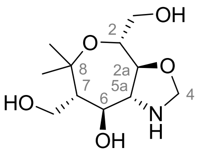

Characteristic signals of **30**:

^{1}H NMR (700 MHz, CD_3OD): δ 1.12, 1.33 (2 s, 3 H each, Me), 2.62 (t, *J* ≈ 9.2 Hz, 1 H, 5a-H), 4.47, 4.66 (2 d, *J* = 4.9 Hz, 1 H each, 5-H) ppm.

Synthesis of (1*S*,4*S*,5*S*,8*S*,9*S*)-9-amino-4-(hydroxymethyl)-3,3-dimethyl-2,6-dioxabicyclo[3.2.2]nonan-8-ol (**28**):

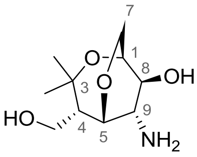

Following typical procedure 6, the bicyclic compound **20** (34 mg, 0.11 mmol), Pd/C (34 mg, 0.03 mmol Pd) were reacted in dry MeOH (12 mL) for 2 d at rt under hydrogen atmosphere. Filtration yielded the amine **28** (23 mg, 96%, ca. >95% pure) as a yellow oil; $[\alpha]_D^{22}$ +21.7 (*c* 1.0, MeOH); TLC [silica gel, CH_2Cl_2/MeOH 3:1] R_f 0.56; ^{1}H NMR (500 MHz, CD_3OD): δ 1.35, 1.47 (2 s, 3 H each, Me), 2.32 (ddd, *J* = 3.9, 5.4, 6.9 Hz, 1 H, 4-H), 3.25 (dd, *J* = 4.2, 7.5 Hz, 1 H, 9-H), 3.62 (dd, *J* = 3.9, 12.3 Hz, 1 H, 4-CH_2), 3.71 (m_c, 1 H, 1-H), 3.72 (dd, *J* = 6.9, 12.3 Hz, 1 H, 4-CH_2), 3.97-3.99 (m, 3 H, 8-H, 7-CH_2), 4.13 (dd, *J* = 4.2, 5.4 Hz, 1 H, 5-H) ppm; ^{13}C NMR (125 MHz,

CD_3OD): δ 9.3, 35.2 (2 q, Me), 55.4 (d, C-4), 58.8 (d, C-9), 61.1 (t, 4-CH_2), 63.5 (t, 7-CH_2), 75.5 (d, C-8), 76.6 (d, C-5), 77.0 (s, C-3), 78.2 (d, C-1) ppm; IR (ATR) $\tilde{\nu}$: 3350, 3290 (OH, NH_2), 2980-2875 (C-H), 1150-1035 (C-O-C) cm^{-1}; ESI-TOF (m/z): [M + Na]$^+$ calcd for $C_{10}H_{19}NO_4Na$, 240.1206; found, 240.1234.

Synthesis of (2*R*,3*S*,4*R*,5*S*,6*S*,7*R*)-4-amino-2,6-bis(hydroxymethyl)-7-phenyloxepan-3,5-diol (31):

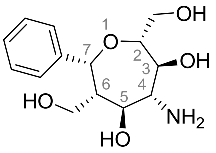

Following typical procedure 5, the bicyclic compound **16** (106 mg, 0.235 mmol), Pd/C (112 mg, 0.105 mmol Pd) were reacted in dry MeOH (13 mL) and AcOH (8.5 mL) for 18 h at rt under hydrogen atmosphere (bicyclic compound **16** was added after 35 min saturation). Filtration yielded the amine **31** (49 mg, 73%) as a colorless solid; mp 200–202 °C; $[\alpha]_D^{22}$ +67.6 (*c* 0.63, MeOH); TLC [silica gel, CH_2Cl_2/7 N NH_3 in MeOH 5:1] R_f 0.07; ^{1}H NMR (500 MHz, Py-d_5): δ 2.90 (m_c, 1 H, 6-H), 3.62 (m_c, 1 H, 4-H), 3.91, 3.97 (AB part of ABX system, J_{AX} = 4.5 Hz, J_{BX} = 8.0 Hz, J_{AB} = 10.5 Hz, 1 H each, 6-CH_2), 4.02-4.09 (m, 2 H, 2-H, 3-H), 4.31 (A part of ABX system, J_{AX} = 4.8 Hz, J_{AB} = 11.5 Hz, 1 H, 2-CH_2), 4.48 (dd, *J* = 7.7, 8.6 Hz, 1 H, 5-H), 4.52 (B part of ABX system, J_{BX} = 1.8 Hz, J_{AB} = 11.5 Hz, 1 H, 2-CH_2), 5.39 (d, *J* = 4.7 Hz, 1 H, 7-H), 7.23-7.33, 7.61-7.63 (2 m, 3 H, 2 H, Ar) ppm, the OH- and NH_2- signals could not be detected; ^{13}C NMR (125 MHz, Py-d_5): δ 50.1 (d, C-4), 52.4 (d, C-6), 62.9 (t, 6-CH_2), 63.0 (d, C-5), 64.9 (t, 2-CH_2), 75.2 (d, C-2 oder C-3), 80.9 (d, C-7), 85.2 (d, C-3 oder C-2), 127.3, 127.6, 128.7, 142.5 (3 d, s, Ar) ppm; IR (ATR) $\tilde{\nu}$: 3370 (OH, NH_2), 3070-

300 (=C-H), 2980-2830 (C-H), 1060, 1020 (C-O-C) cm^{-1}; ESI-TOF (m/z): [M + Na]$^+$ calcd for $C_{14}H_{21}NO_5Na$, 306.1312; found, 306.1326.

Synthesis of (2*S*,3*S*,4*R*,5*S*,6*S*)-4-amino-6-(hydroxymethyl)-7,7-dimethyl-2-(propoxymethyl)oxepan-3,5-diol (32):

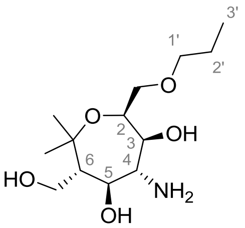

Following procedure 5, the bicyclic compound **18** (110 mg, 0.304 mmol), Pd/C (115 mg, 0.108 mmol Pd) were reacted in dry MeOH (10 mL) and AcOH (3.3 mL) for 16 h at rt under hydrogen atmosphere. Filtration yielded the amine **32** (84 mg, quant., ca. 80% pure) as a yellow oil; $[\alpha]_D^{22}$ +25.0 (*c* 0.80, MeOH); TLC [silica gel, CH_2Cl_2/7 N NH_3 in MeOH 20:1] R_f 0.05; ^{1}H NMR (700 MHz, CD_3OD): δ 0.92-0.94 (m, 3 H, 3'-H), 1.19, 1.32 (2 s, 3 H each, Me), 1.55-1.60 (m, 2 H, 2'-H), 1.75 (m_c, 1 H, 6-H), 2.86 (t, *J* ≈ 6.9 Hz, 1 H, 4-H), 3.39-3.45 (m, 3 H, 1'-H, 2-CH_2), 3.48 (m_c, 1 H, 3-H), 3.55 (dd, *J* = 4.9, 10.9 Hz, 1 H, 2-CH_2), 3.63 (dd, *J* = 2.3, 11.2 Hz, 1 H, 6-CH_2), 3.67-3.69 (m, 2 H, 5-H, 6-CH_2), 3.83 (m_c, 1 H, 2-H) ppm; ^{13}C NMR (175 MHz, CD_3OD): δ 11.0 (q, C-3'), 21.1 (q, Me), 24.0 (t, C-2'), 31.6 (q, Me), 60.1 (d, C-6), 64.1 (d, C-4), 64.3 (t, 6-CH_2), 71.5 (d, C-5), 72.2 (d, C-2), 72.4 (t, 2-CH_2), 74.1 (t, C-1'), 77.15 (s, C-1), 77.22 (d, C-3) ppm; IR (ATR) $\tilde{\nu}$: 3380 (OH, NH_2), 2980-2875 (C-H), 1105, 1060, 1030 (C-O-C) cm^{-1}; ESI-TOF (m/z): [M + H]$^+$ calcd for $C_{13}H_{28}NO_5$, 278.1962; found, 278.2002.

Synthesis of (2*S*,3*S*,4*R*,5*S*,6*S*)-4-amino-2-(aminomethyl)-6-(hydroxymethyl)-7,7-dimethyloxepan-3,5-diol (33):

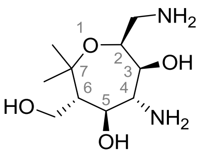

Following typical procedure 5, the bicyclic compound **24** (62 mg, 0.18 mmol), Pd/C (66 mg, 0.06 mmol Pd) were reacted in dry MeOH (8 mL) and AcOH (2 mL) for 18 h at rt under hydrogen atmosphere. Filtration yielded amine **33** (30 mg, 72%, ca. >95% pure) as a yellow oil; TLC [silica gel, CH_2Cl_2/7 N NH_3 in MeOH 5:1] R_f 0.02; ^{1}H NMR (700 MHz, CD_3OD): δ 1.18, 1.35 (2 s, 3 H each, Me), 1.77 (ddd, J = 3.1, 6.4, 9.6 Hz, 1 H, 6-H), 2.67-2.74 (m, 2 H, 2-CH_2), 2.85 (dd, J = 6.5, 8.7 Hz, 1 H, 4-H), 3.44 (dd, J = 2.5, 6.5 Hz, 1 H, 3-H), 3.64-3.69 (m, 4 H, 2-H, 5-H, 6-CH_2) ppm; ^{13}C NMR (175 MHz, CD_3OD): δ 21.3, 31.6 (2 q, Me), 44.4 (t, 2-CH_2), 59.6 (d, C-6), 64.1 (d, C-4), 64.3 (t, 6-CH_2), 71.1, 73.7 (2 d, C-2, C-5)*, 77.1 (d, C-3), 77.5 (s, C-7) ppm, *an unambiguous assignment of the signals was not possible; ESI-TOF (m/z): $[M + H]^+$ calcd for $C_{10}H_{22}N_2O_4$, 235.1652; found, 235.1661.

References

[1] Helms, M.; Schade, W.; Pulz, R.; Watanabe, T.; Al-Harrasi, A.; Fišera, L.; Hlobilová, I.; Zahn, G.; Reissig, H.-U. *Eur. J. Org. Chem.* **2005**, 1003–1019. doi:10.1002/ejoc.200400627

[2] Borch, R. F.; Bernstein, M. D.; Durst, H. D. *J. Am. Chem. Soc.* **1971**, *93*, 2897–2904. doi: 10.1021/ja00741a013

[3] Dondoni, A.; Franco, S.; Junquera, F.; Merchán, F. L.; Merino, P.; Tejero, T. *Synth. Commun.* **1994**, *24*, 2537–2550. doi:10.1080/00397919408010565

[4] Abushanab, E.; Vemishetti, P.; Leiby, H R. W.;. Singh, K.; Mikkilineni, A. B.; Wu, D. C.-J.; Saibaba, R.; Panzica, R. P. *J. Org. Chem*. **1988**, *53*, 2598–2602. doi: 10.1021/jo00246a037

[5] Al-Harrasi, A.; Reissig, H.-U. *Angew. Chem.* **2005**, *117*, 6383–6387; *Angew. Chem. Int. Ed*. **2005**, *44*, 6227–6231. doi: 10.1002/ange.200501127; doi: 10.1002/anie.200501127

[6] 1,3-Dienes are usual side products of the 1,2-oxazine formation and they are always detected in small amounts. For an explanation of their formation, see ref 1.

[7] Schade, W. Ph.D. Thesis, Freie Universität Berlin, Germany, 1998.

[8] Bressel, B. Ph.D. Thesis, Freie Universität Berlin, Germany, 2008.
