# Supplementary material for: Synthesis of new enantiopure poly(hydroxy)aminooxepanes as building blocks for multivalent carbohydrate mimetics
Source: Beilstein J Org Chem. 2014 Jan 20;10:213–23. doi: 10.3762/bjoc.10.17 (PMC3943604; doi:10.3762/bjoc.10.17)

Supporting Information File 2

for

Synthesis of new enantiopure poly(hydroxy)aminooxepanes as building blocks for multivalent carbohydrate mimetics

Léa Bouché, Maja Kandziora and Hans-Ulrich Reissig*

Address: Freie Universität Berlin, Institut für Chemie und Biochemie, Takustrasse 3, D-14195 Berlin, Germany

Email: Hans-Ulrich Reissig - hans.reissig@chemie.fu-berlin.de

* Corresponding author

Characterization data ^{1}H NMR and ^{13}C NMR spectra of synthesized compounds

Table of contents:

- NMR spectra of the ethyl ester s2
- NMR spectra of (*Z*)-nitrones and 1,2 oxazines s3
- NMR spectra of bicyclic 1,2-oxazines s9
- NMR spectra of oxepanes s29
- NOE experiments s36

^{1}H NMR (500 MHz, CDCl$_3$)

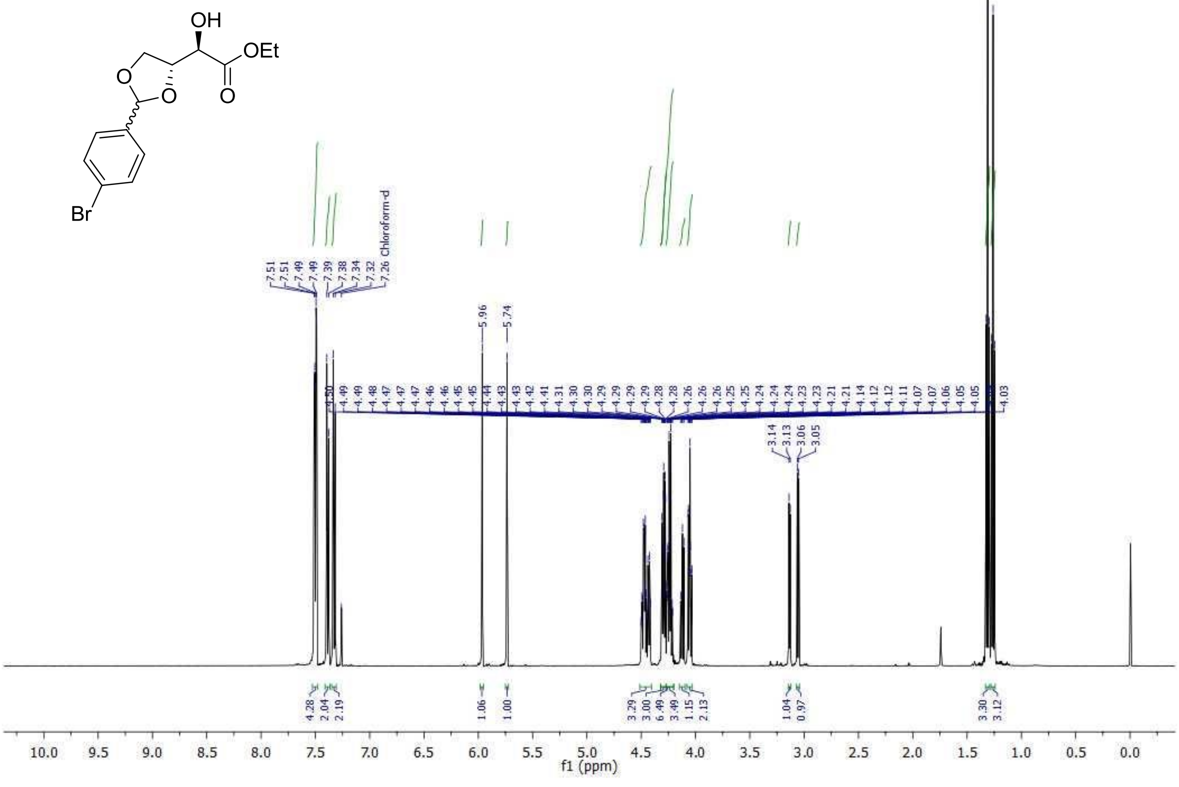

^{13}C NMR (125 MHz, CDCl$_3$)

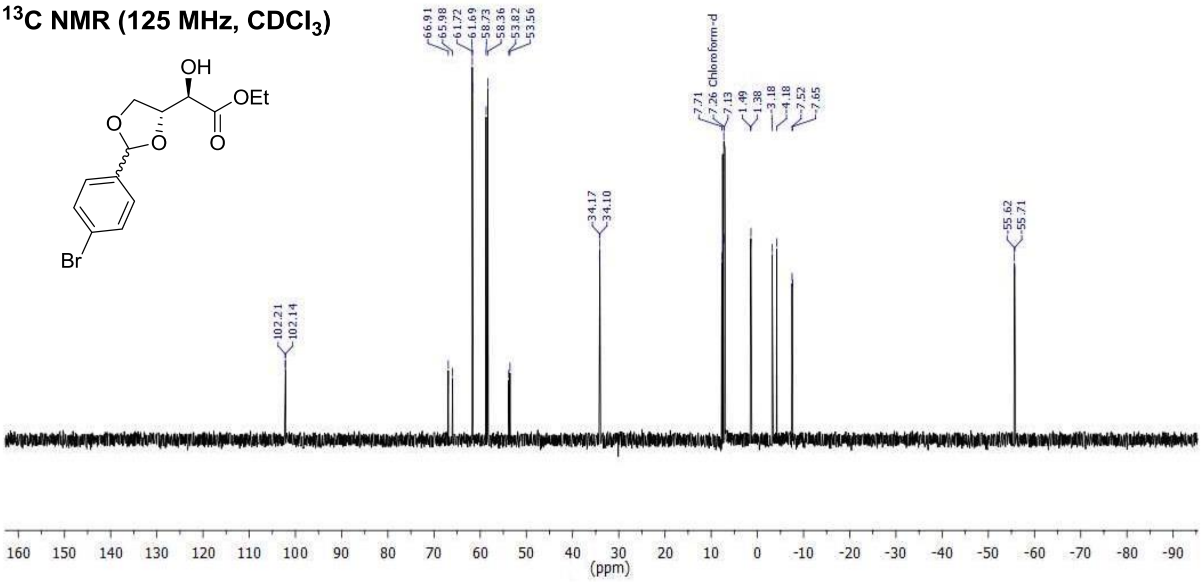

^{1}H NMR (500 MHz, $CDCl_3$)

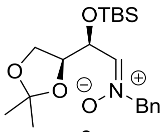

6

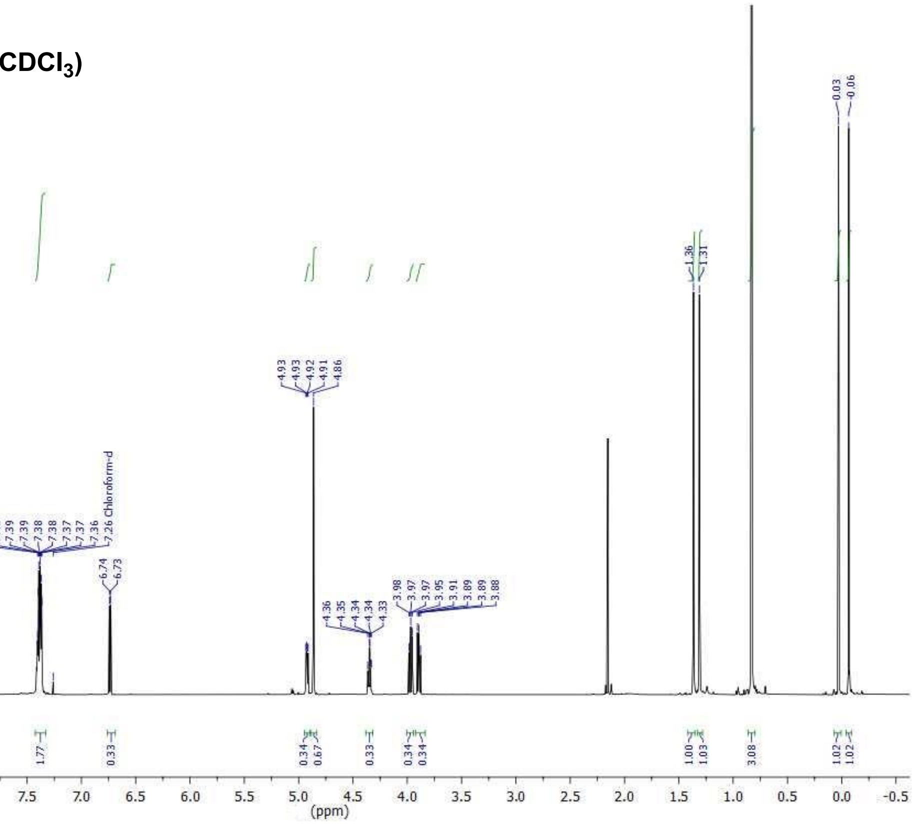

^{13}C NMR (125 MHz, $CDCl_3$)

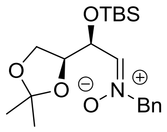

6

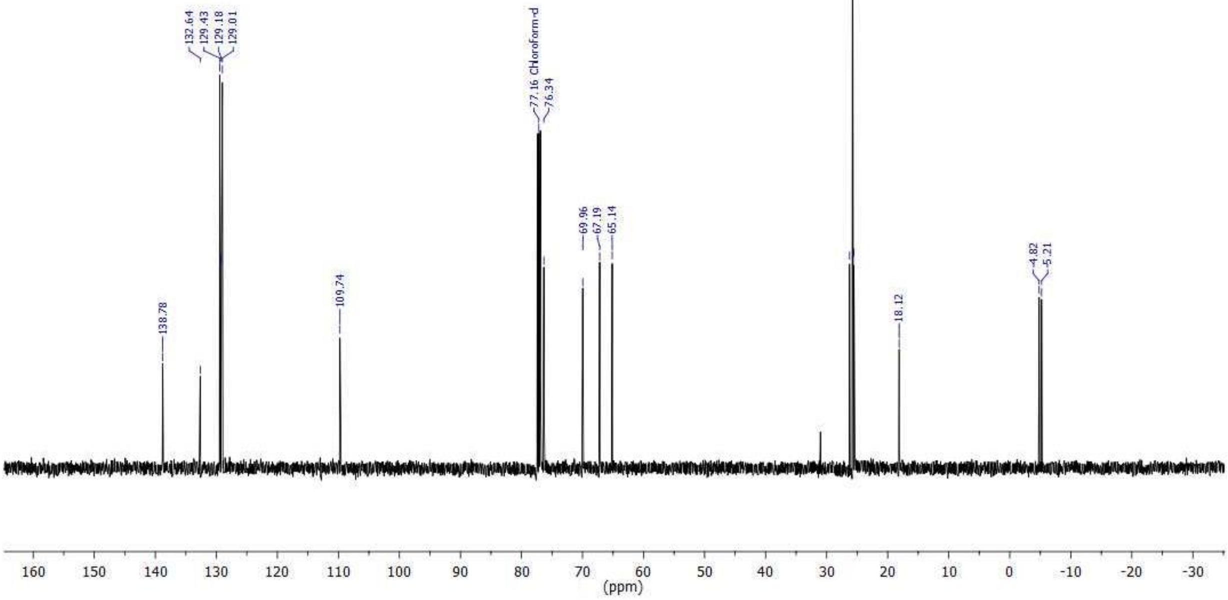

^{1}H NMR (500 MHz, CDCl$_3$)

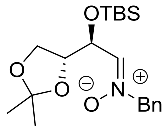

8

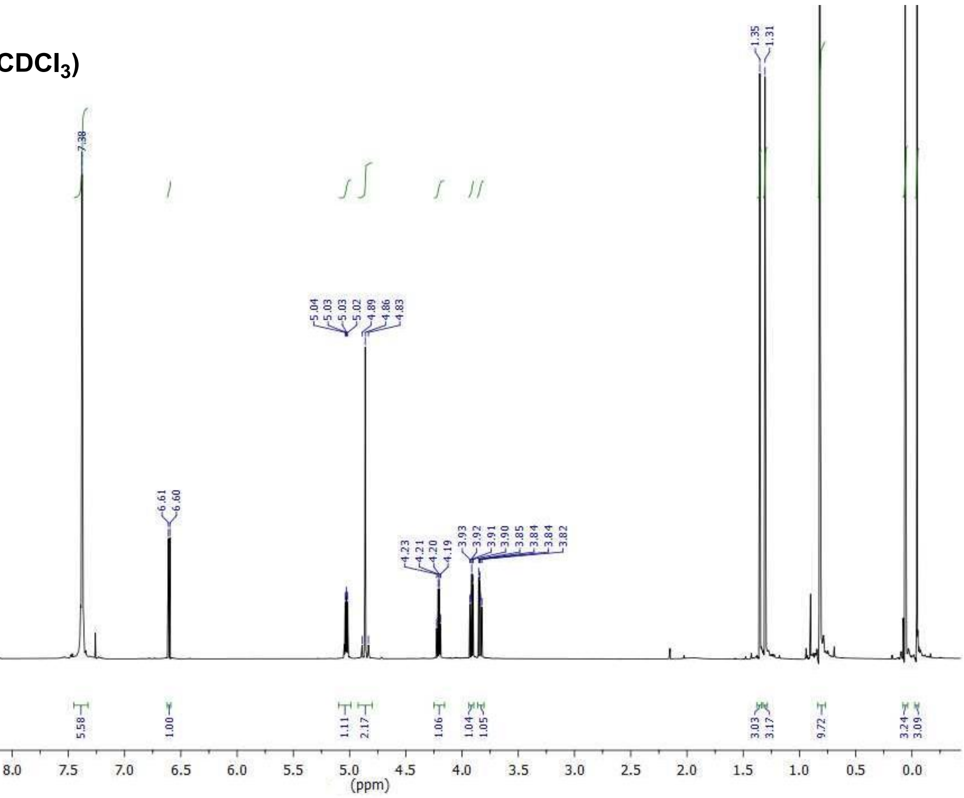

^{13}C NMR (125 MHz, CDCl$_3$)

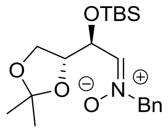

8

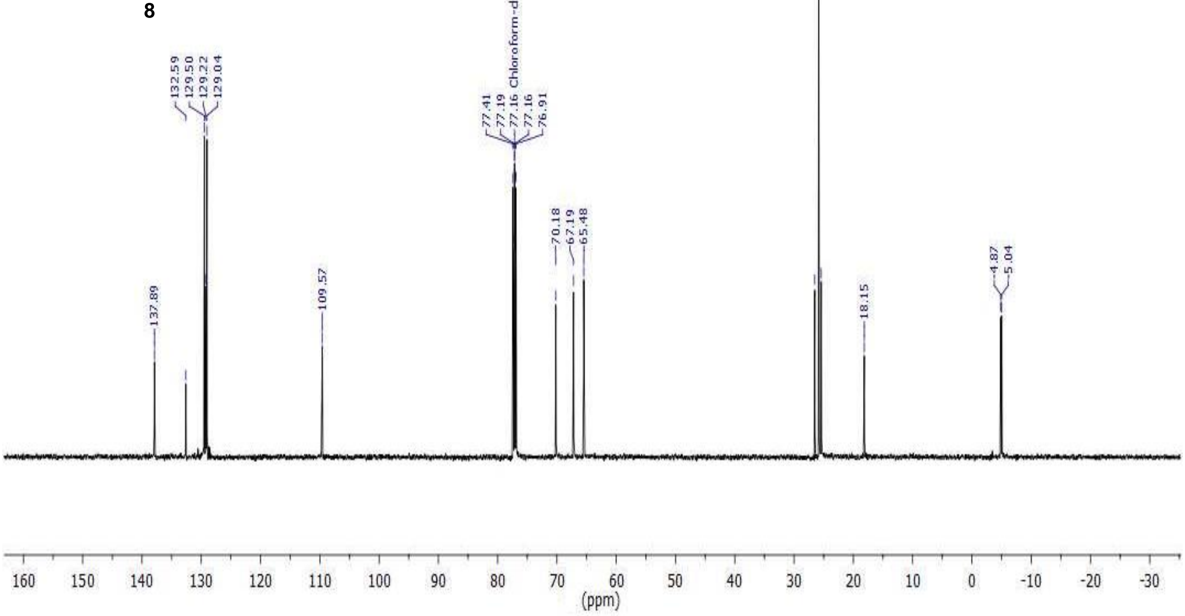

^{1}H NMR (500 MHz, CDCl$_3$)

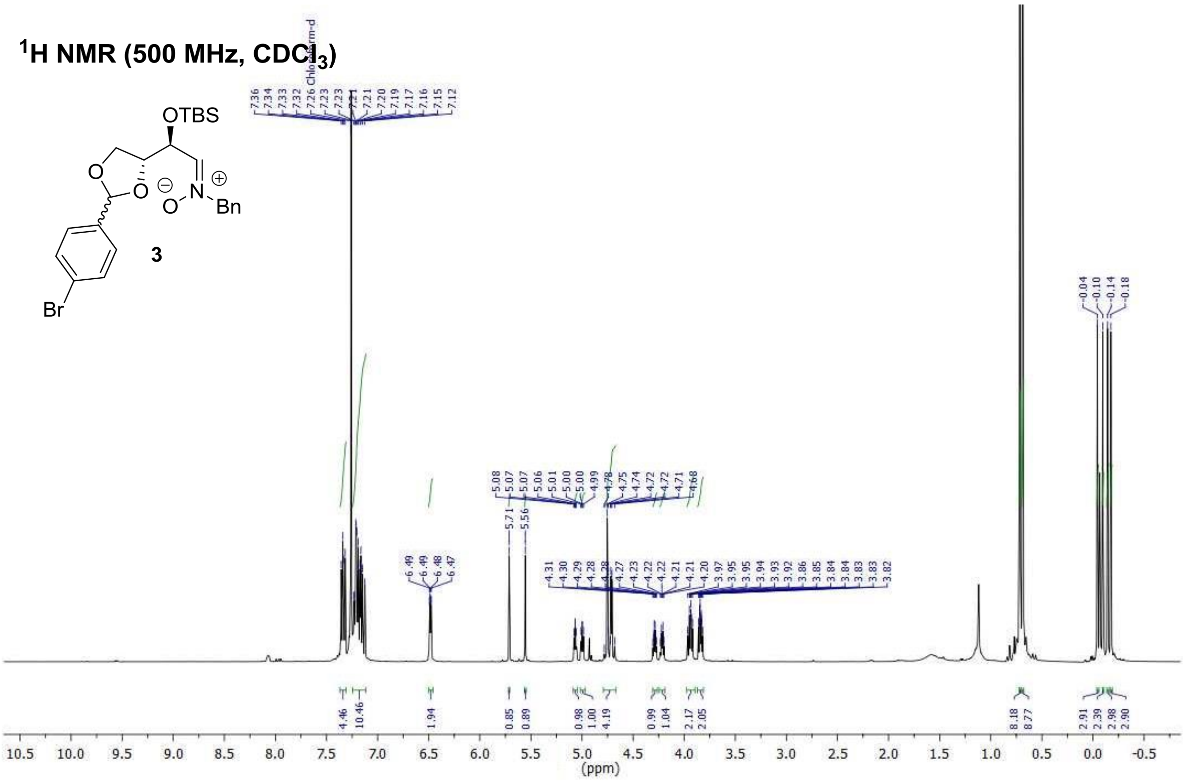

^{13}C NMR (125 MHz, CDCl$_3$)

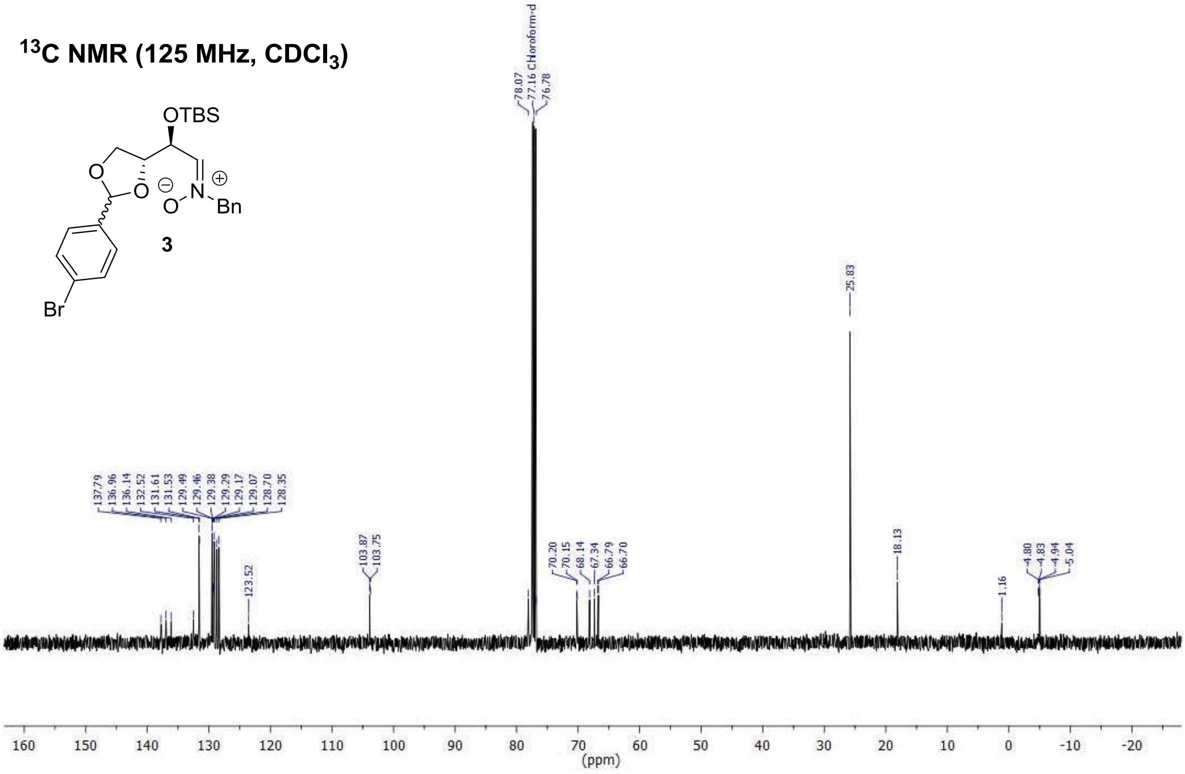

^{1}H NMR (700 MHz, $CDCl_3$)

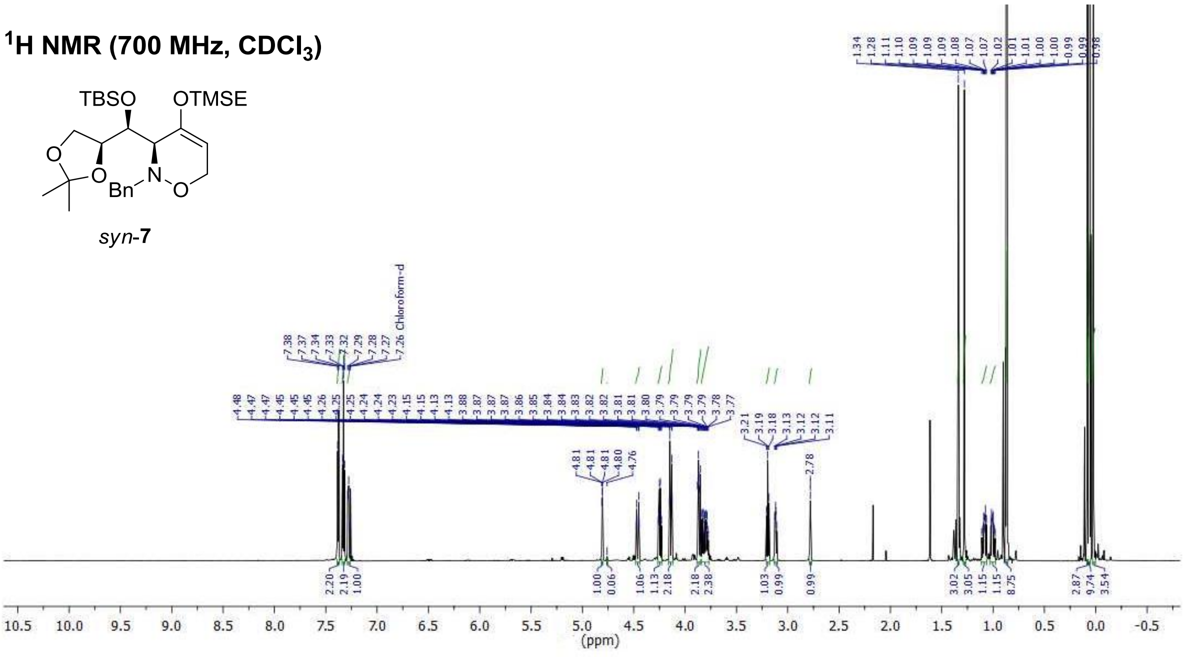

syn-**7**

^{1}H NMR (175 MHz, $CDCl_3$)

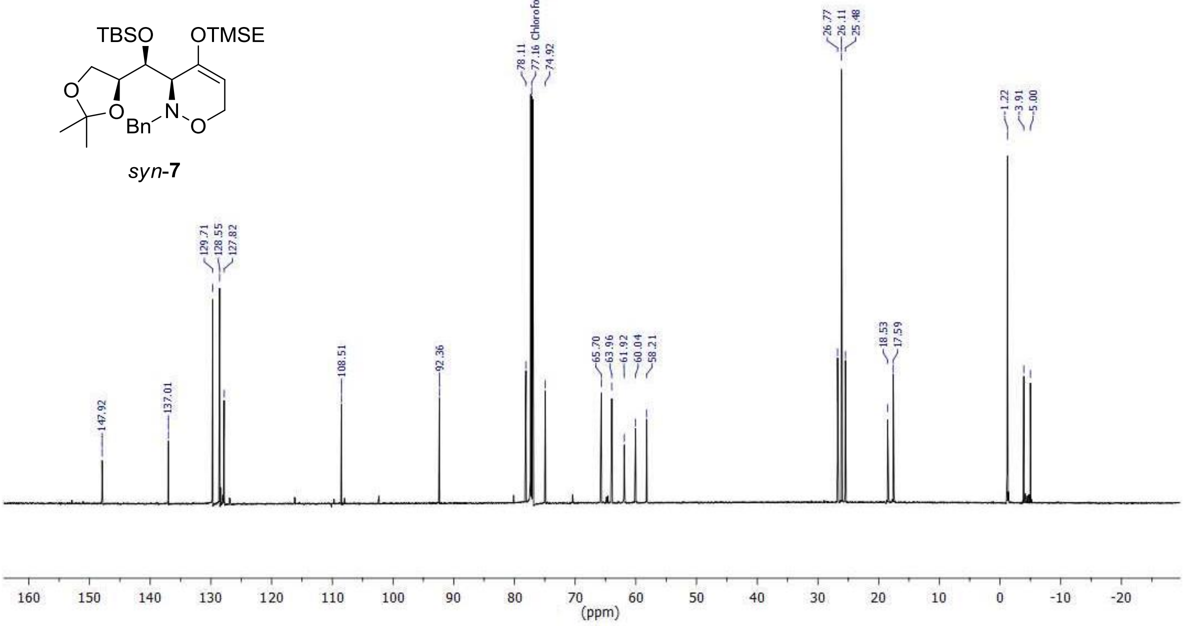

syn-**7**

^{1}H NMR (700 MHz, CDCl$_3$)

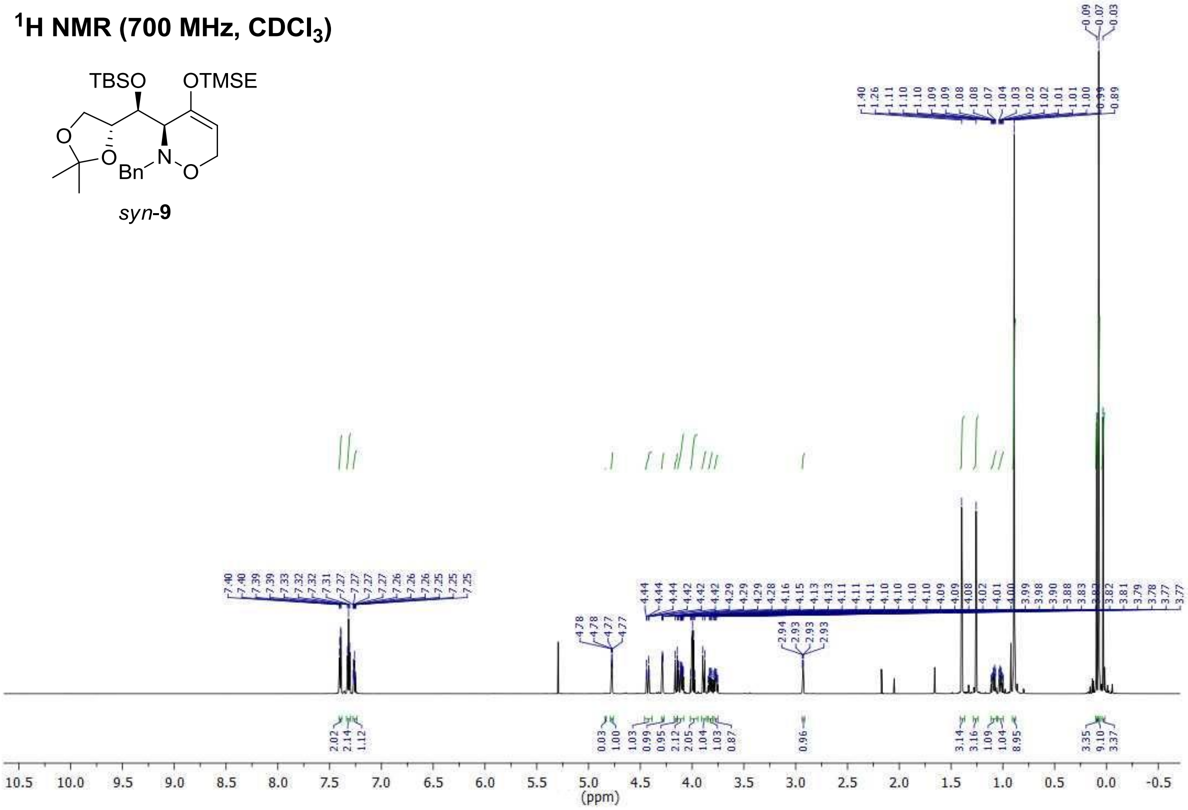

^{13}C NMR (175 MHz, CDCl$_3$)

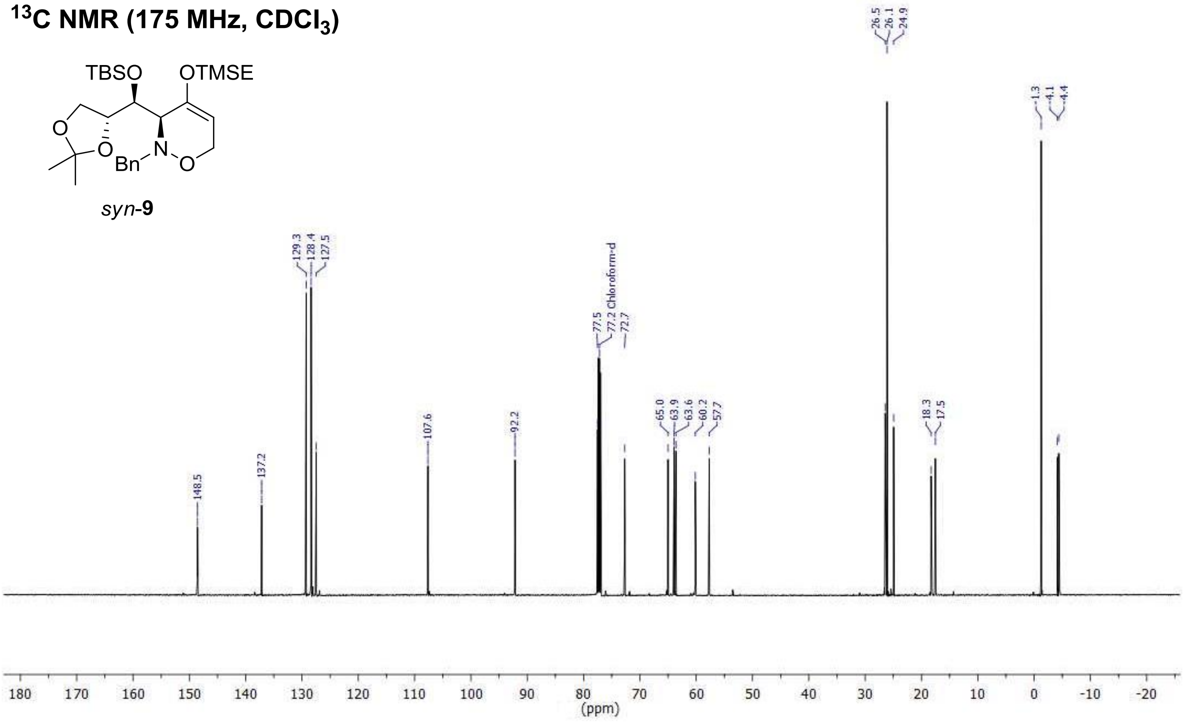

^{1}H NMR (700 MHz, $CDCl_3$)

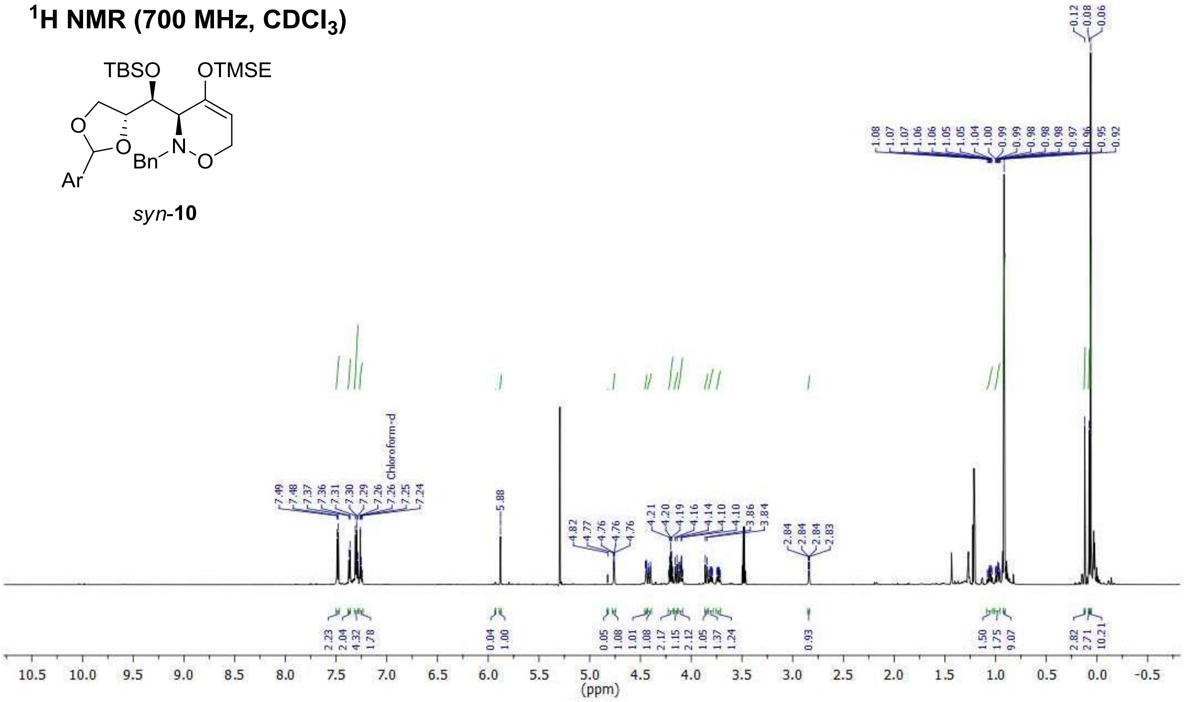

syn-**10**

^{13}C NMR (175 MHz, $CDCl_3$)

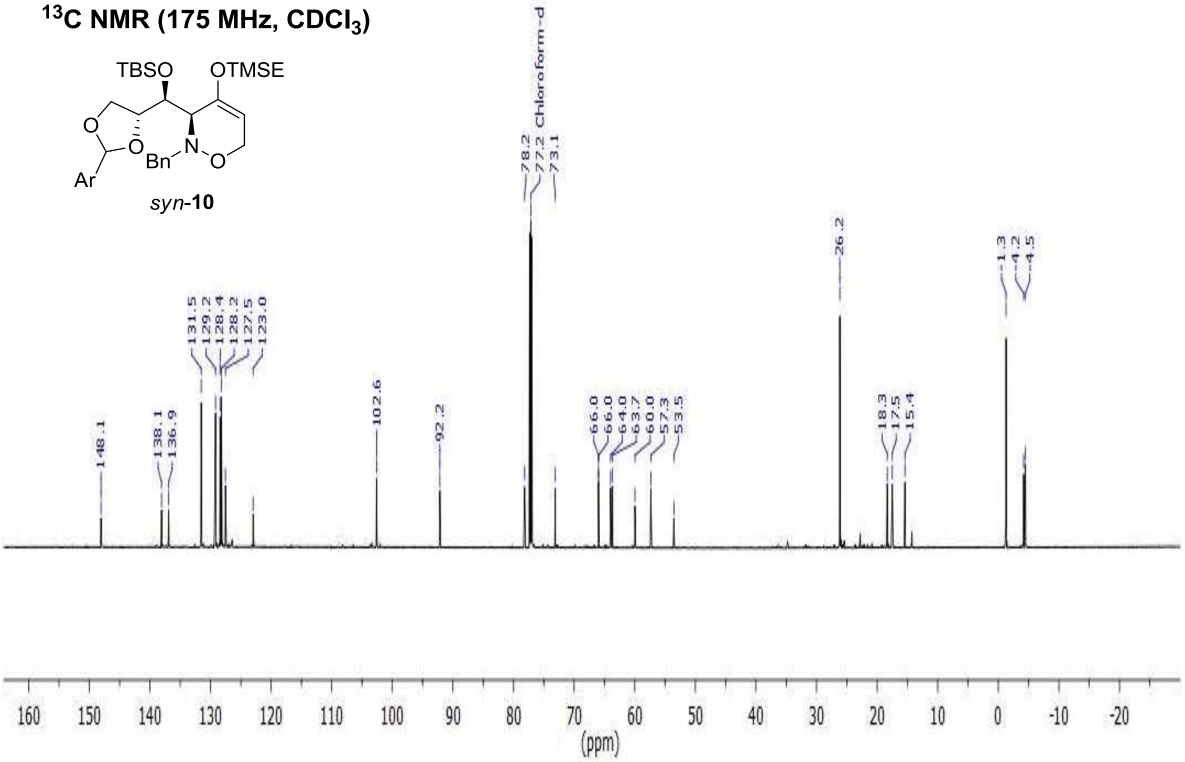

syn-**10**

^{1}H NMR (500 MHz, $CDCl_3$)

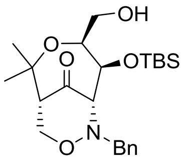

11

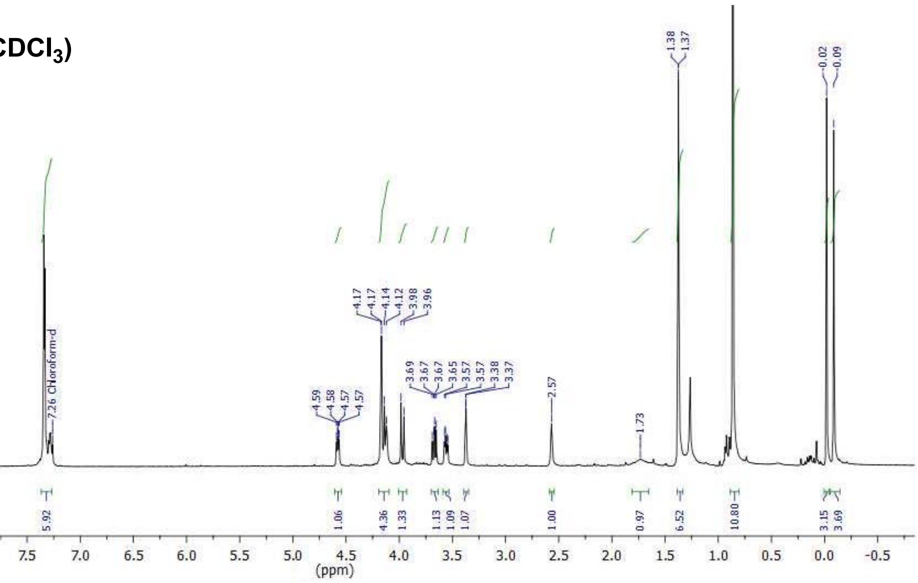

^{13}C NMR (125 MHz, $CDCl_3$)

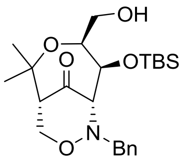

11

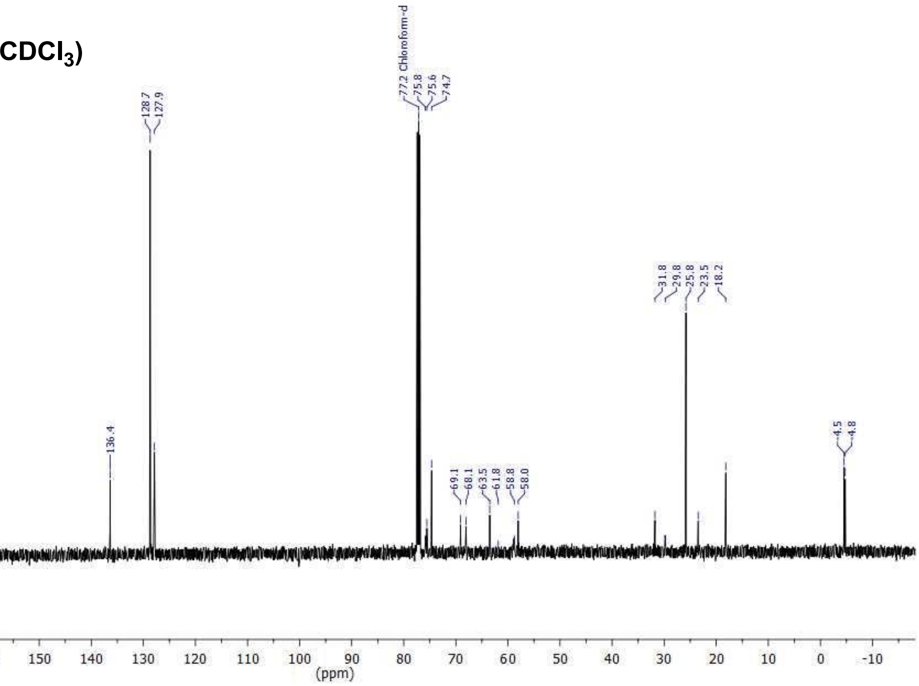

^{1}H NMR (500 MHz, $CDCl_3$)

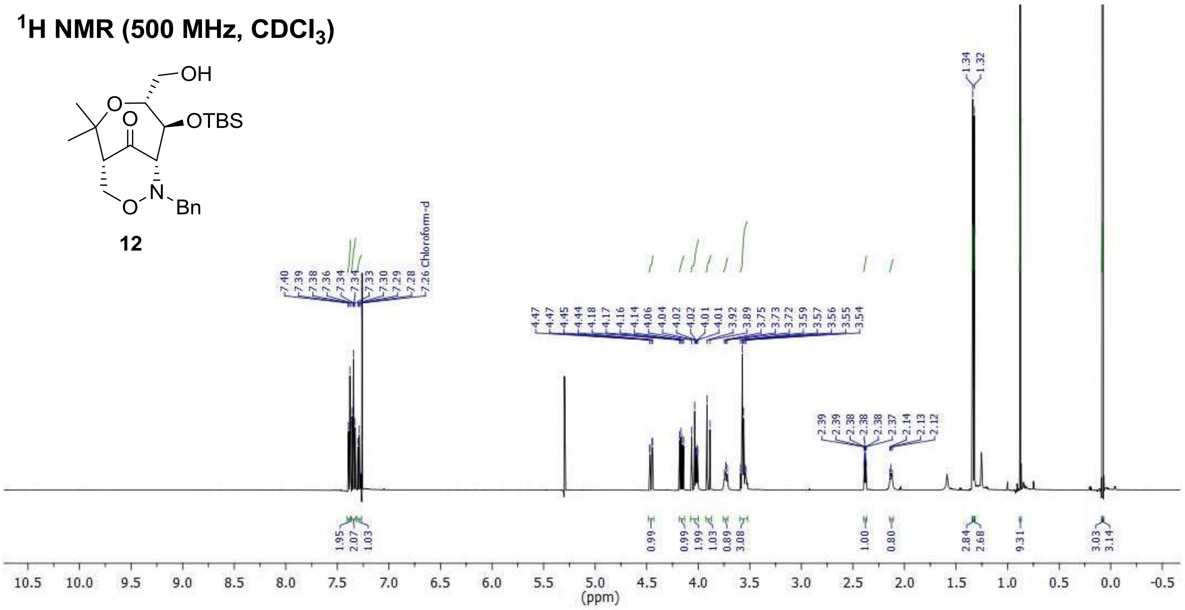

^{13}C NMR (125 MHz, $CDCl_3$)

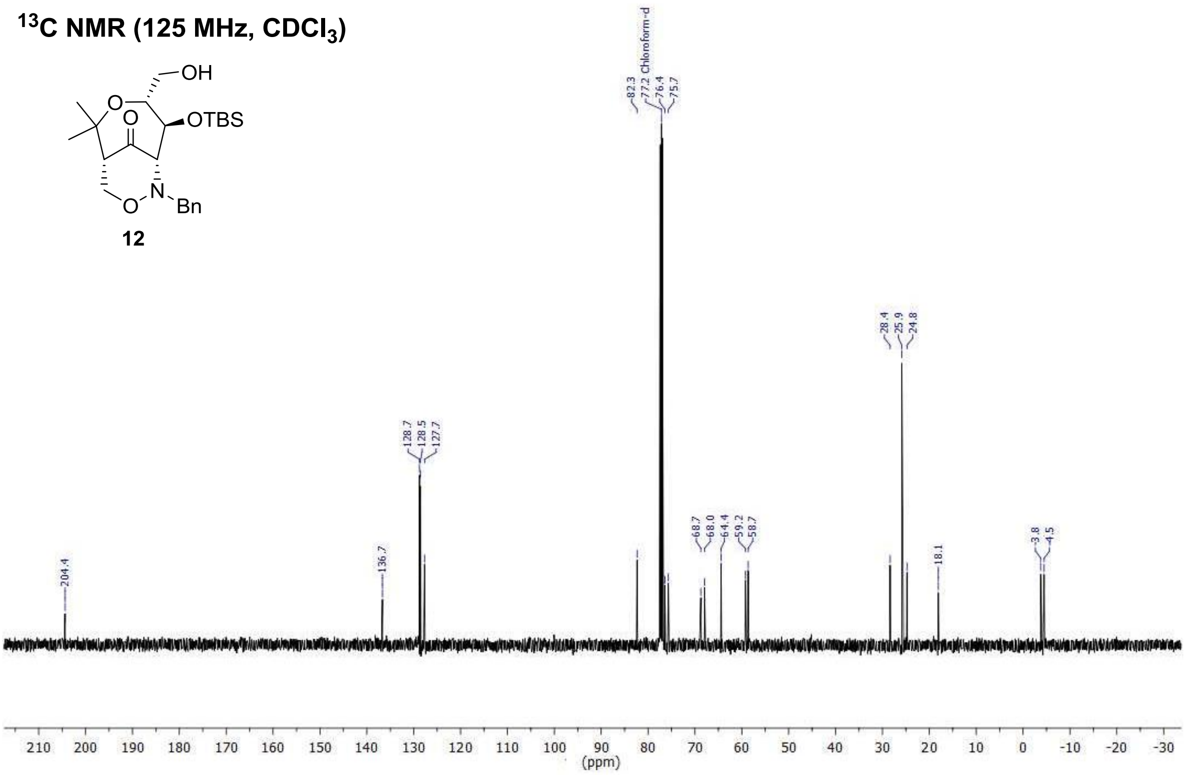

^{1}H NMR (500 MHz, $CDCl_3$)

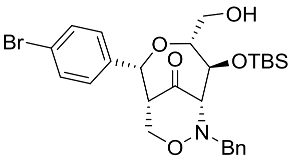

13

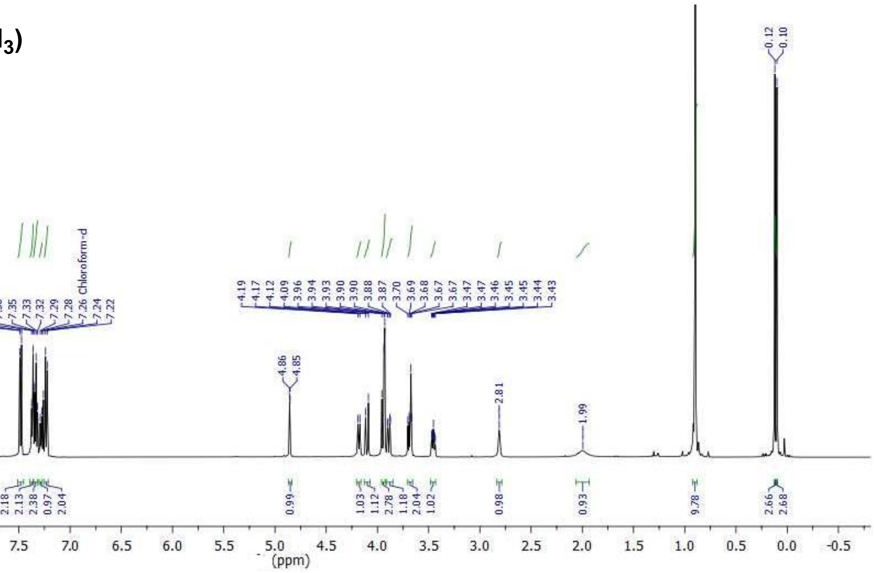

^{13}C NMR (125 MHz, $CDCl_3$)

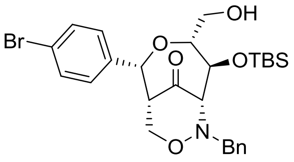

13

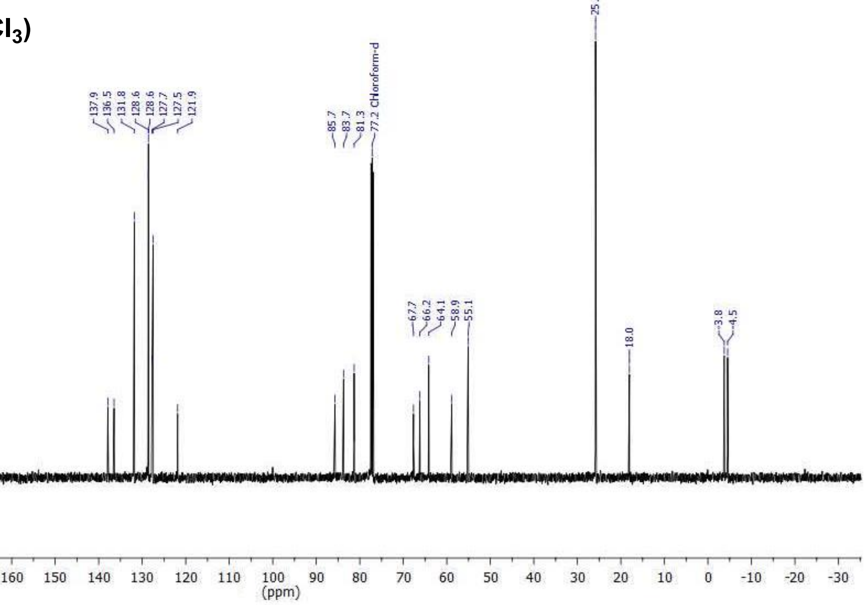

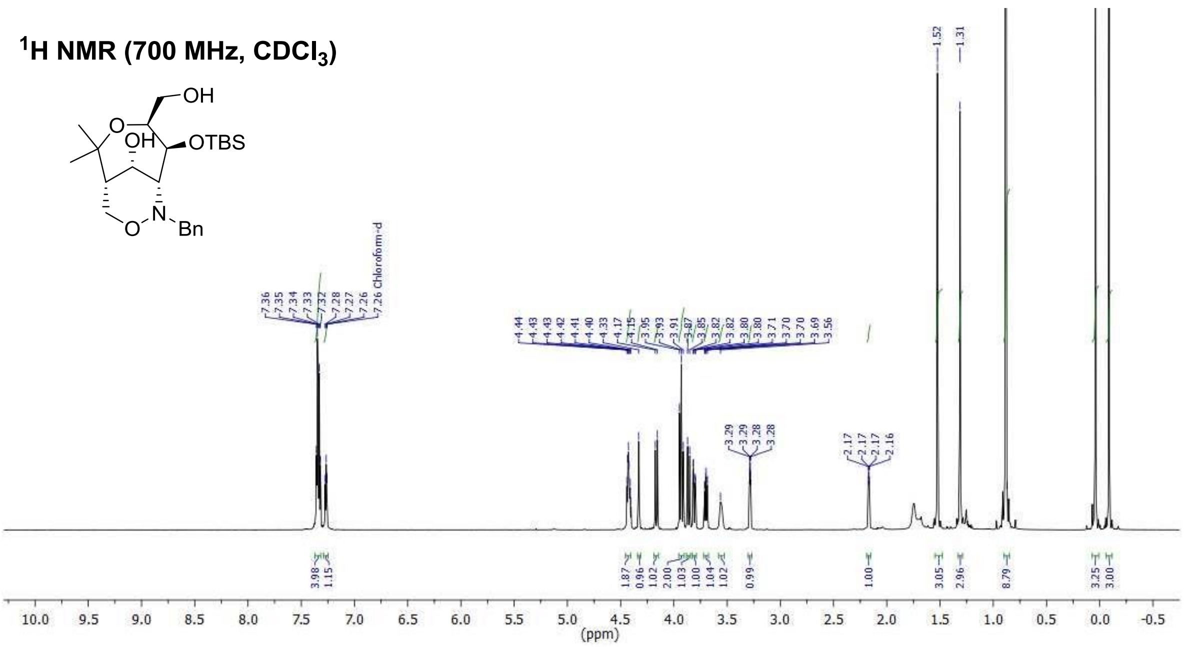

1H NMR (700 MHz, CDCl3)
OH
OTBS
OH
N
Bn
O
O
Chloroform-d
(ppm)

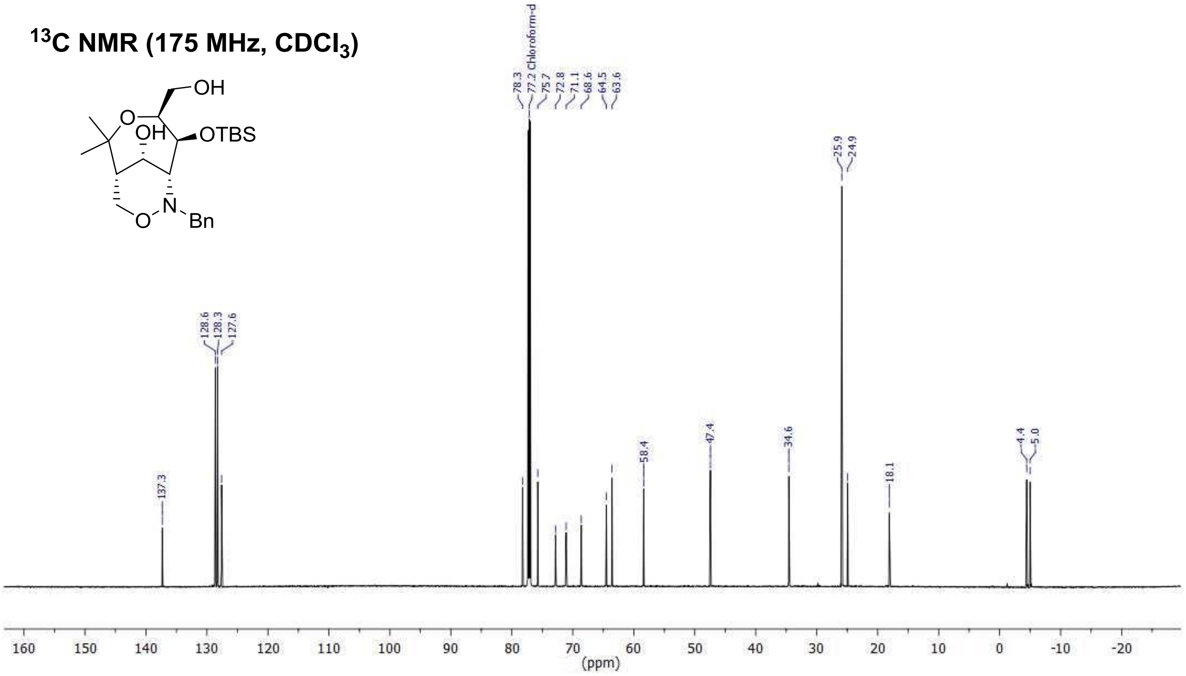

13C NMR (175 MHz, CDCl3)
OH
OTBS
OH
N
Bn
O
O
Chloroform-d
(ppm)

^{1}H NMR (500 MHz, $CDCl_3$)

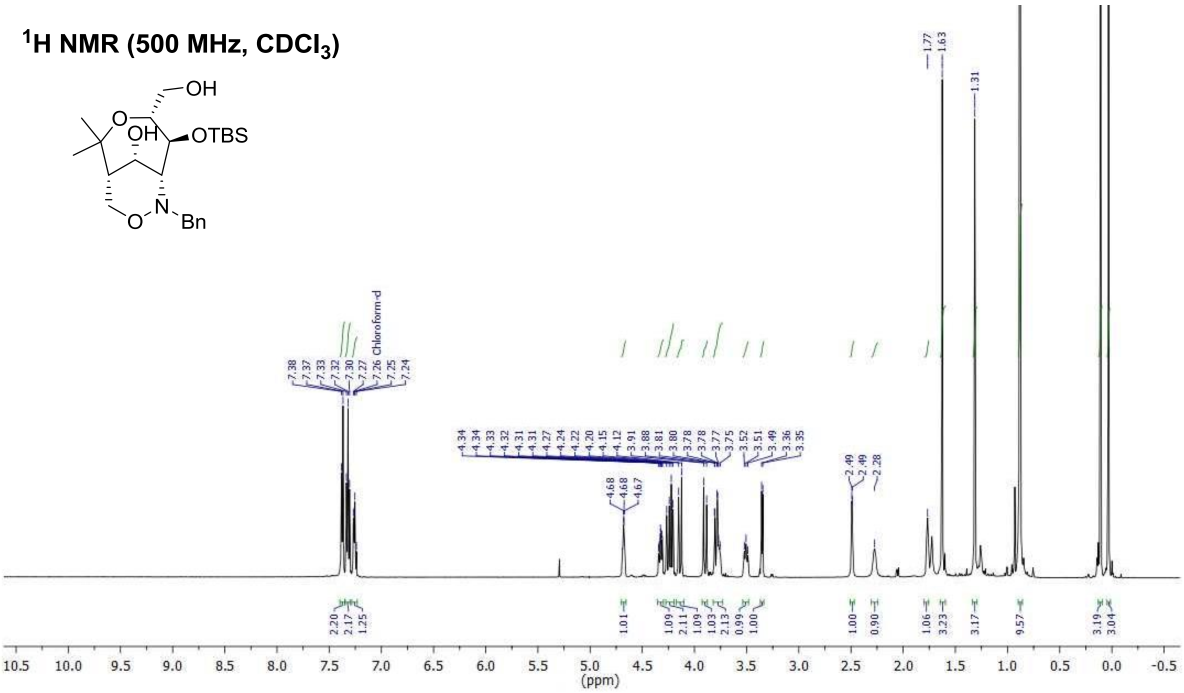

^{13}C NMR (125 MHz, $CDCl_3$)

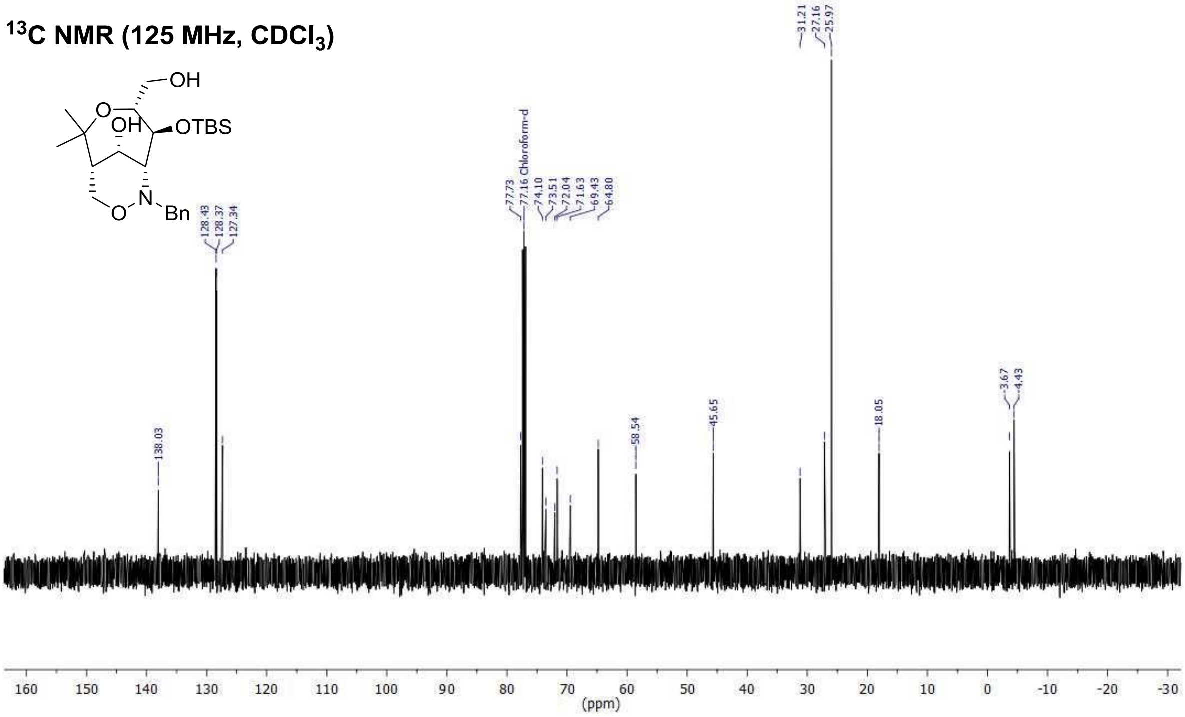

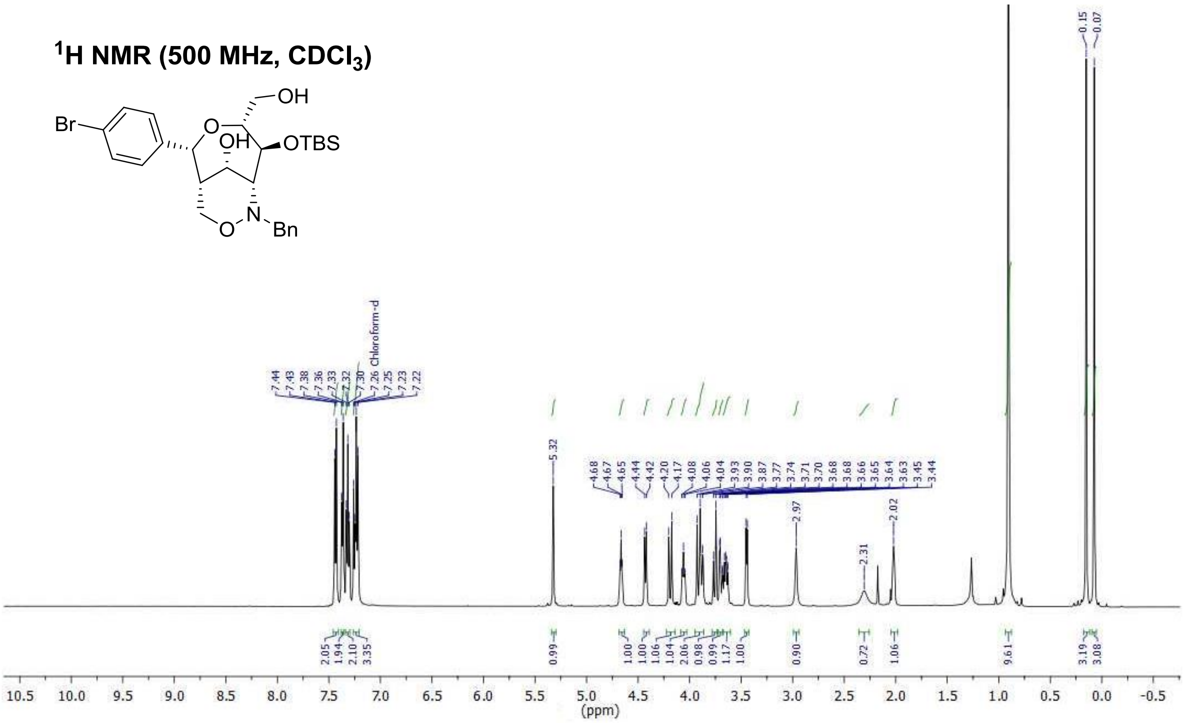

1H NMR (500 MHz, CDCl3)
Br
OH
OTBS
N
Bn
O
(ppm)

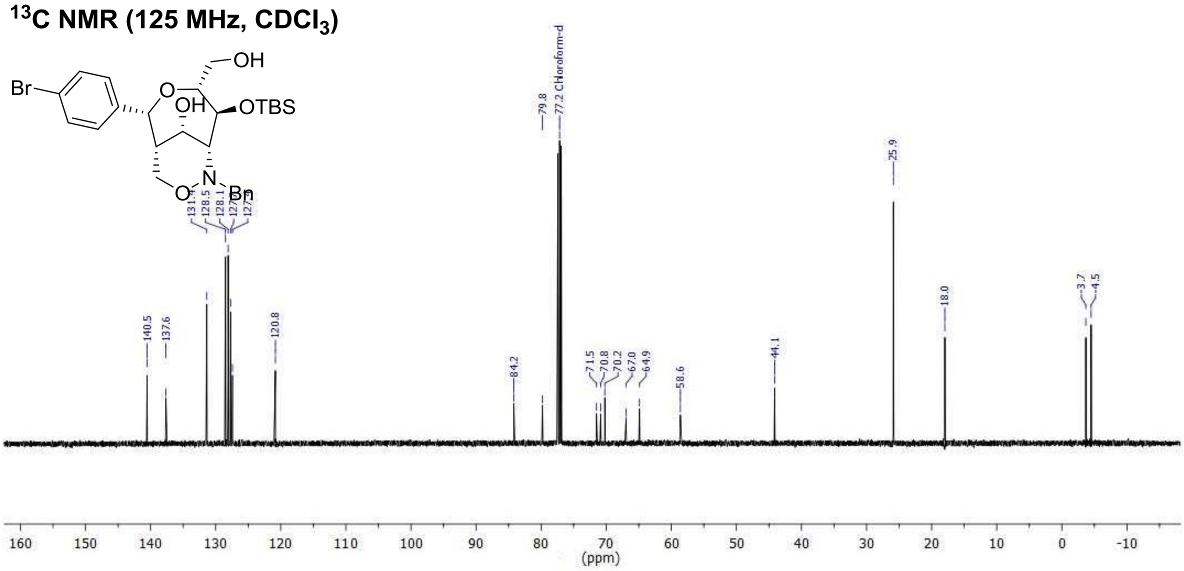

13C NMR (125 MHz, CDCl3)
Br
OH
OTBS
N
Bn
O
(ppm)

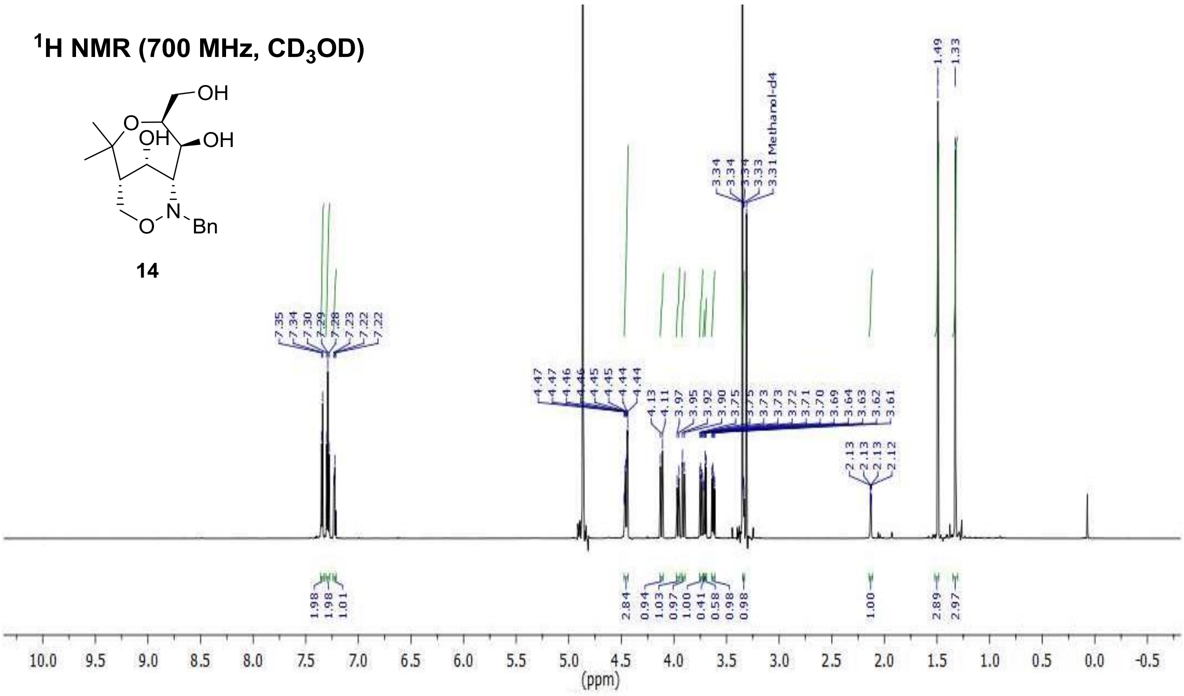

1H NMR (700 MHz, CD3OD)
OH
OH
OH
O
O
N
Bn
14
7.35 7.34 7.30 7.29 7.28 7.23 7.22 7.22
4.47 4.47 4.46 4.45 4.45 4.44 4.44
4.13 4.11 3.97 3.95 3.92 3.90 3.75 3.75 3.73 3.73 3.72 3.71 3.70 3.69 3.64 3.63 3.62 3.61
3.34 3.34 3.34 3.33 3.31 Methanol-d4
2.13 2.13 2.13 2.12
1.49
1.33
1.98 1.98 1.01 2.84 0.94 1.03 0.97 1.00 0.41 0.58 0.98 0.98 1.00 2.89 2.97
(ppm)

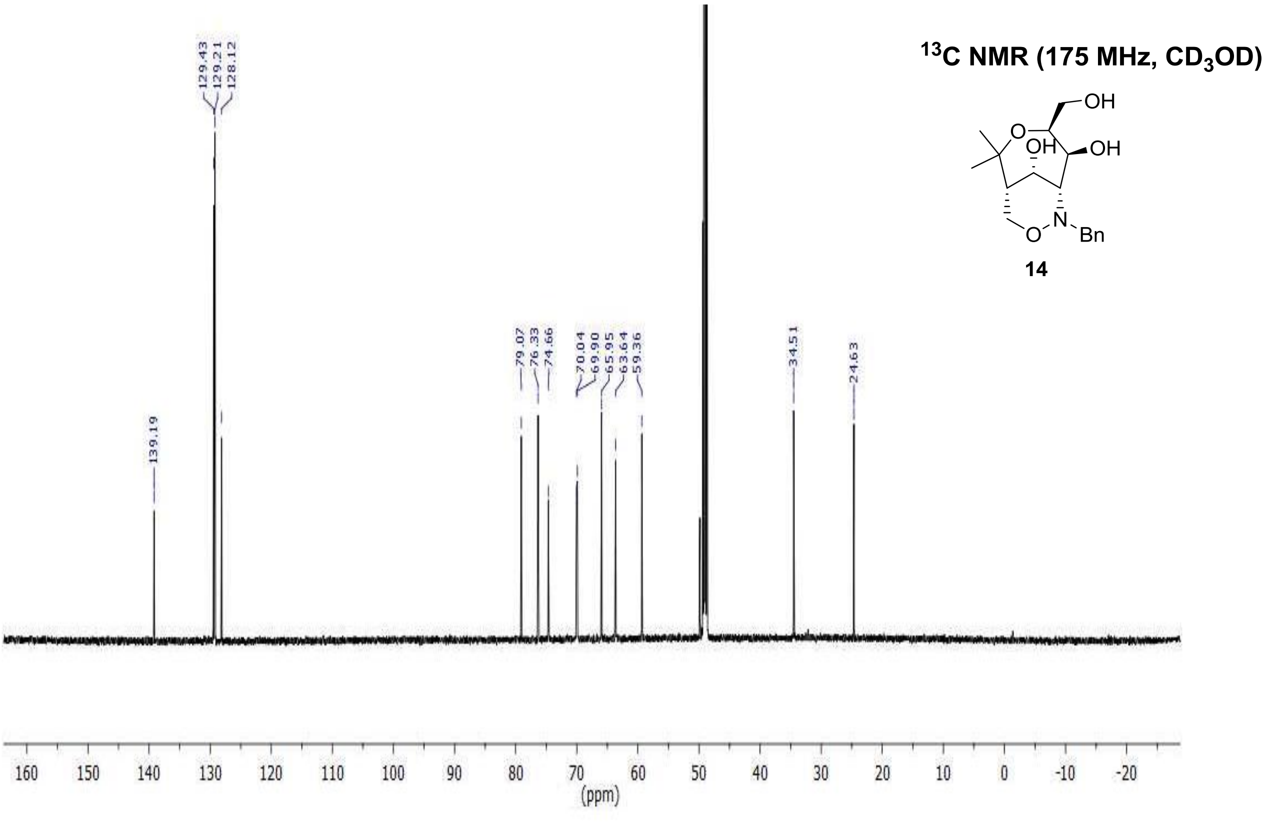

13C NMR (175 MHz, CD3OD)
OH
OH
OH
O
O
N
Bn
14
139.19
129.43 129.21 128.12
79.07 76.33 74.66 70.04 69.90 65.95 63.64 59.36
34.51
24.63
(ppm)

^{1}H NMR (700 MHz, CD_3OD)

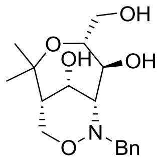

15

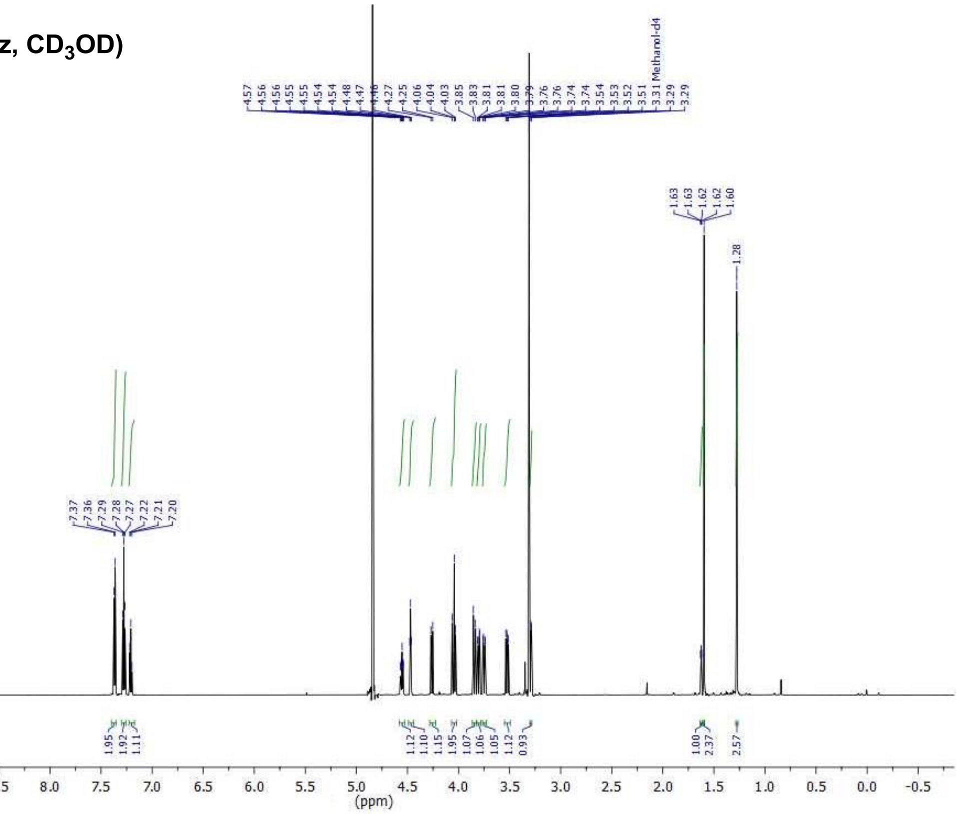

^{13}C NMR (175 MHz, CD_3OD)

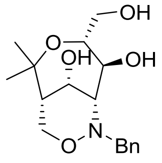

15

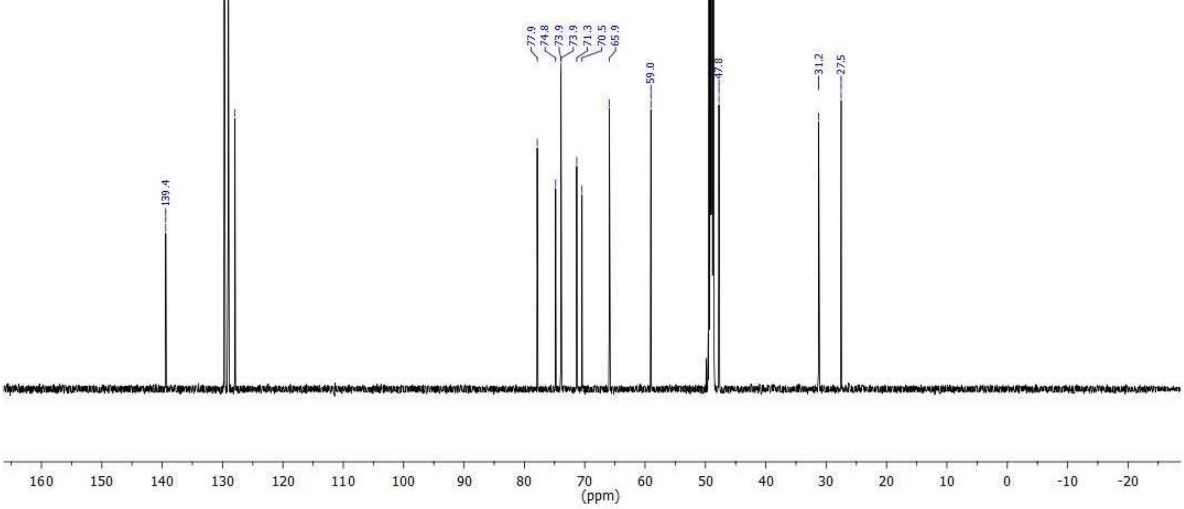

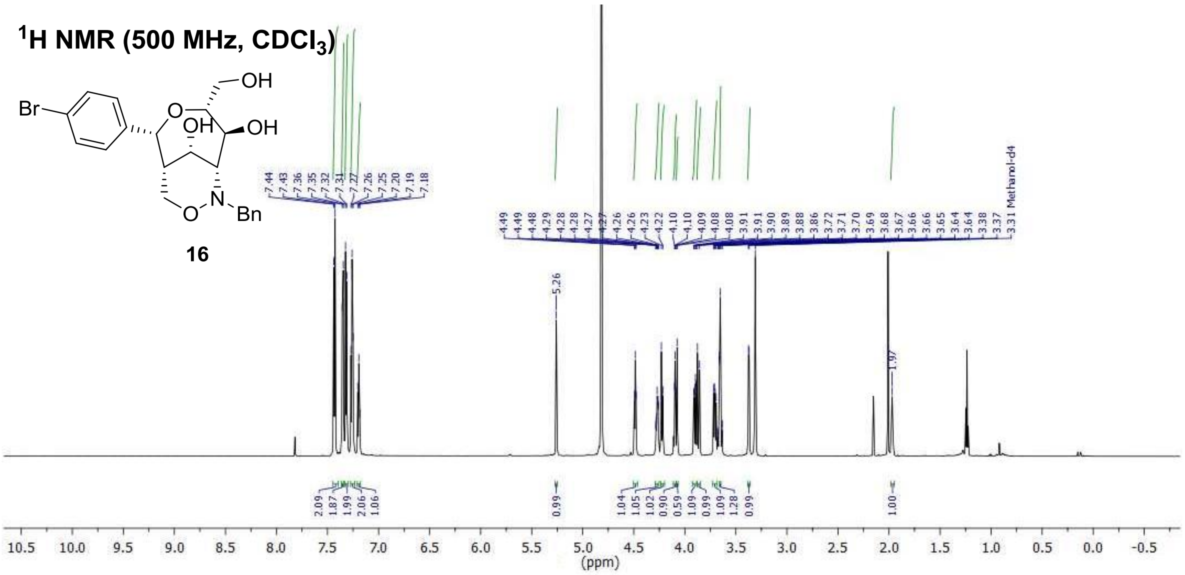

^{1}H NMR (500 MHz, CDCl$_3$)
16
(ppm)

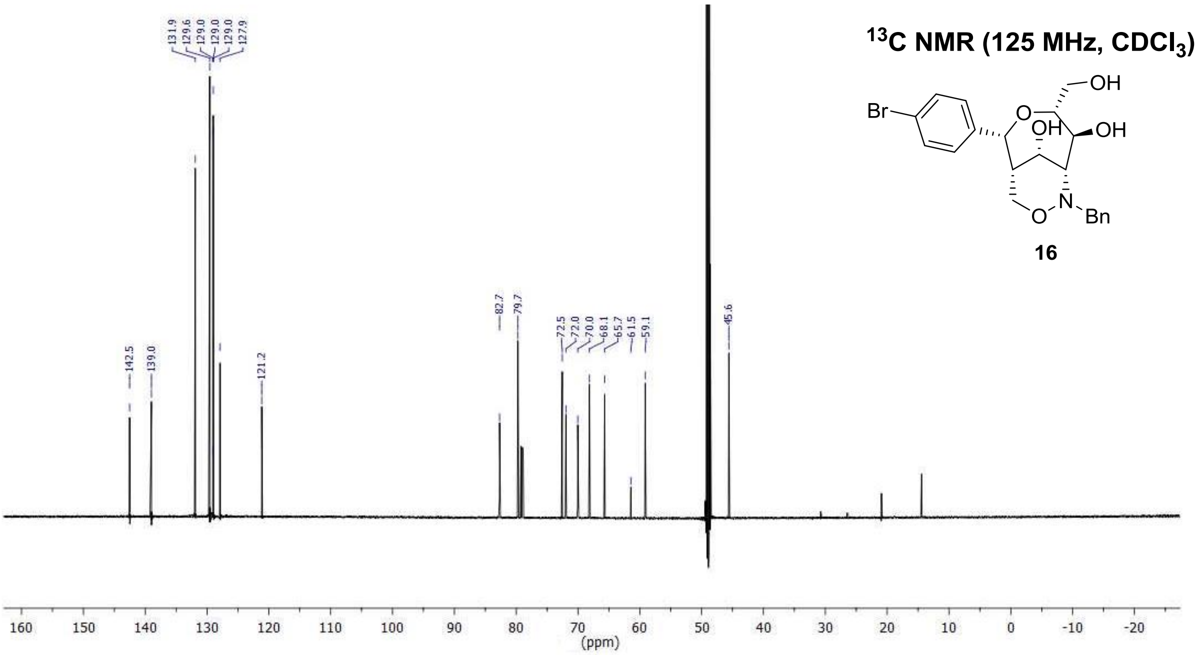

^{13}C NMR (125 MHz, CDCl$_3$)
16
(ppm)

^{1}H NMR (500 MHz, $CDCl_3$)

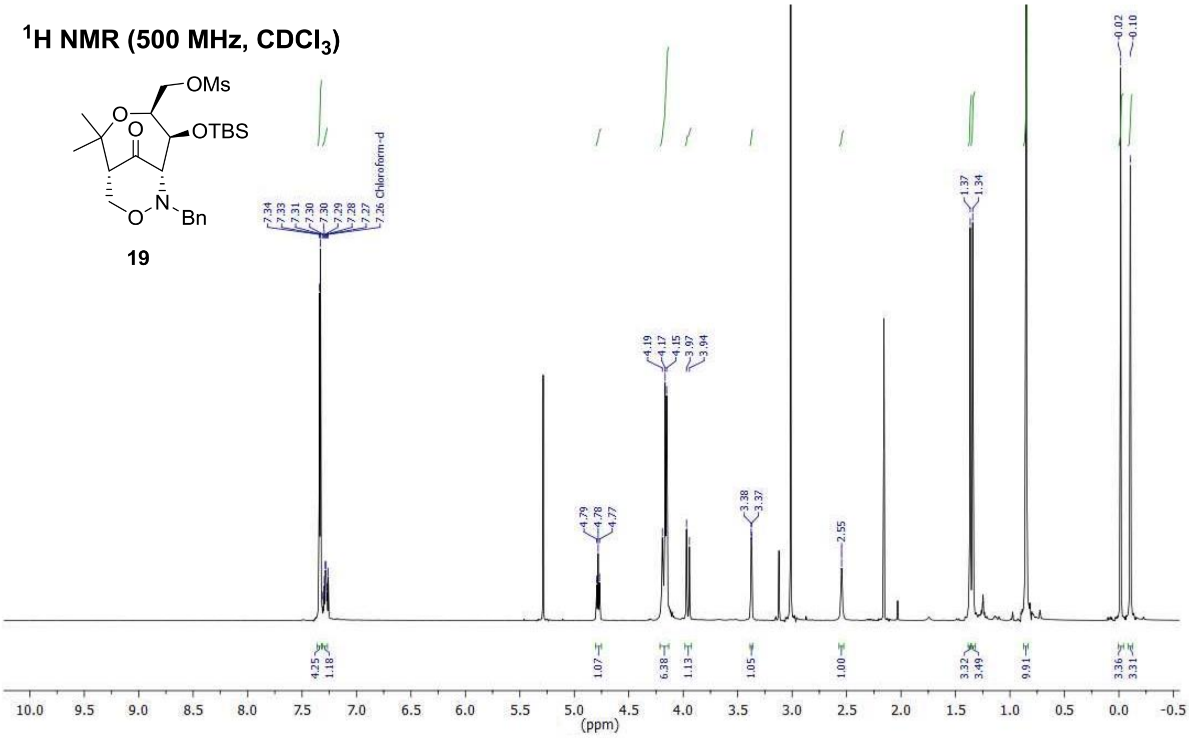

^{13}C NMR (125 MHz, $CDCl_3$)

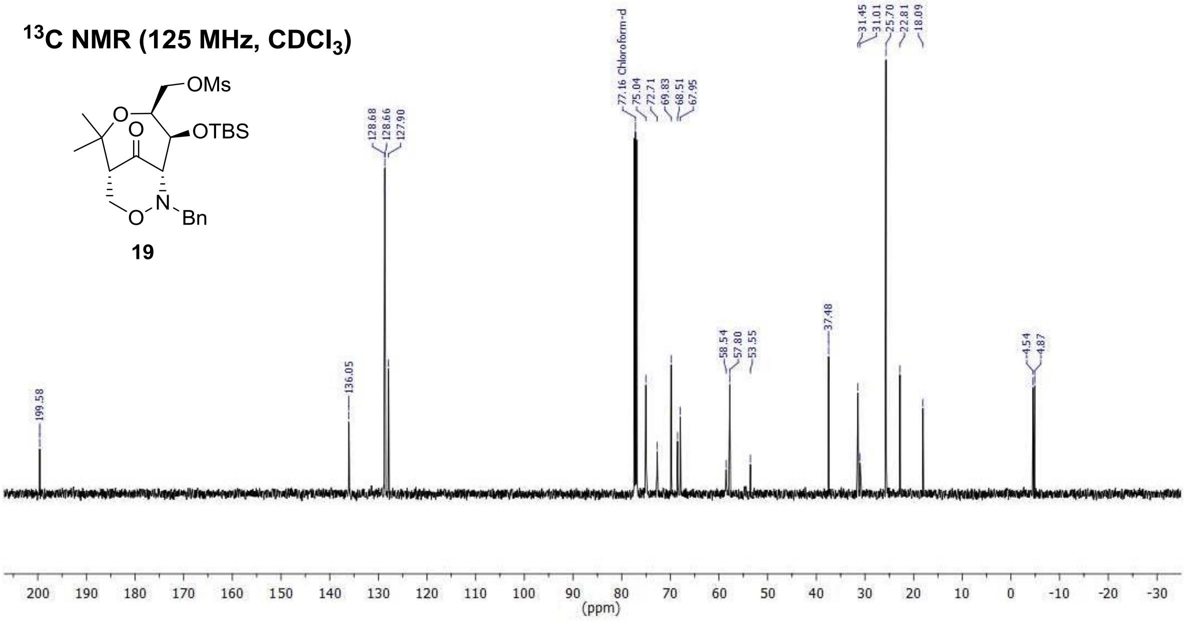

^{1}H NMR (500 MHz, $CDCl_3$)

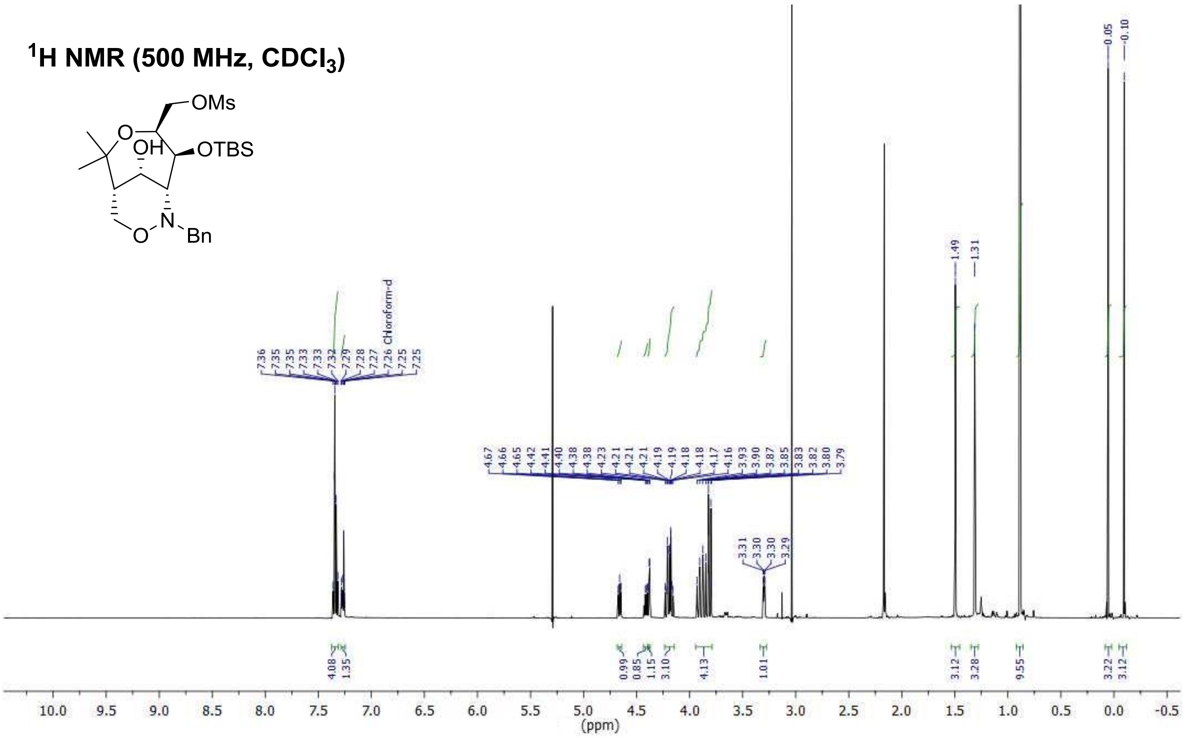

^{13}C NMR (125 MHz, $CDCl_3$)

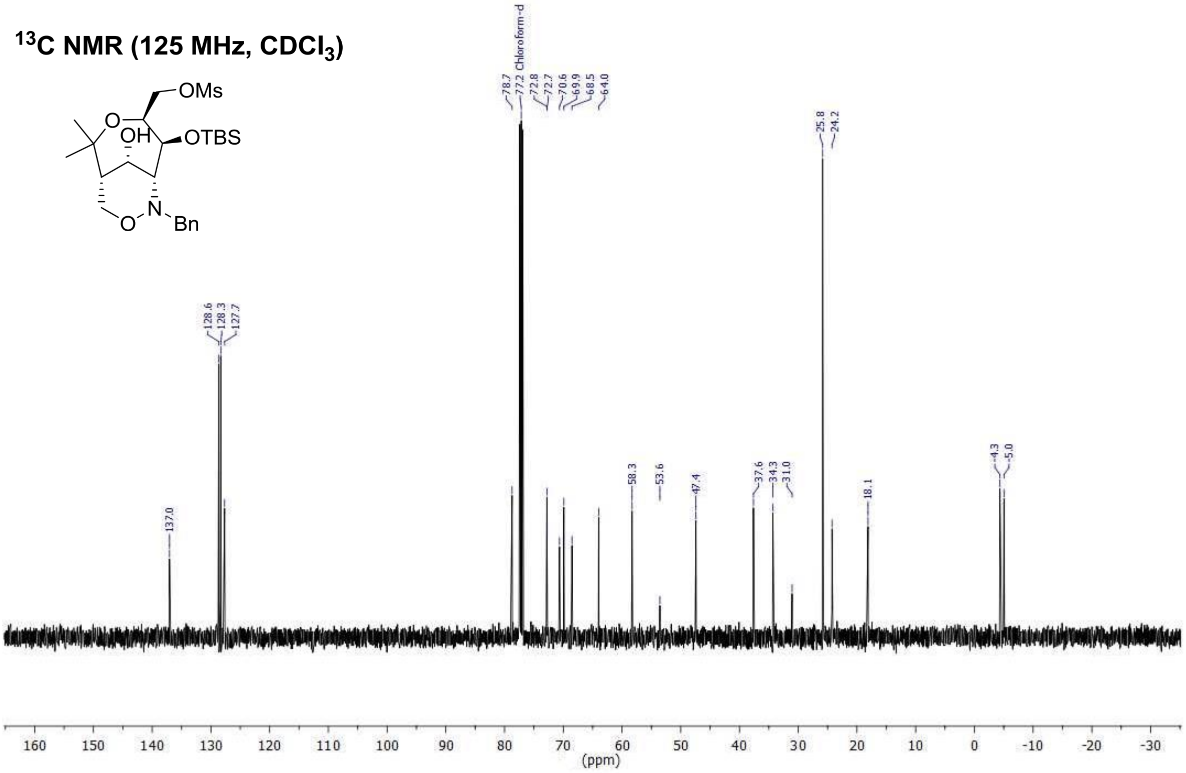

^{1}H NMR (500 MHz, $CDCl_3$)

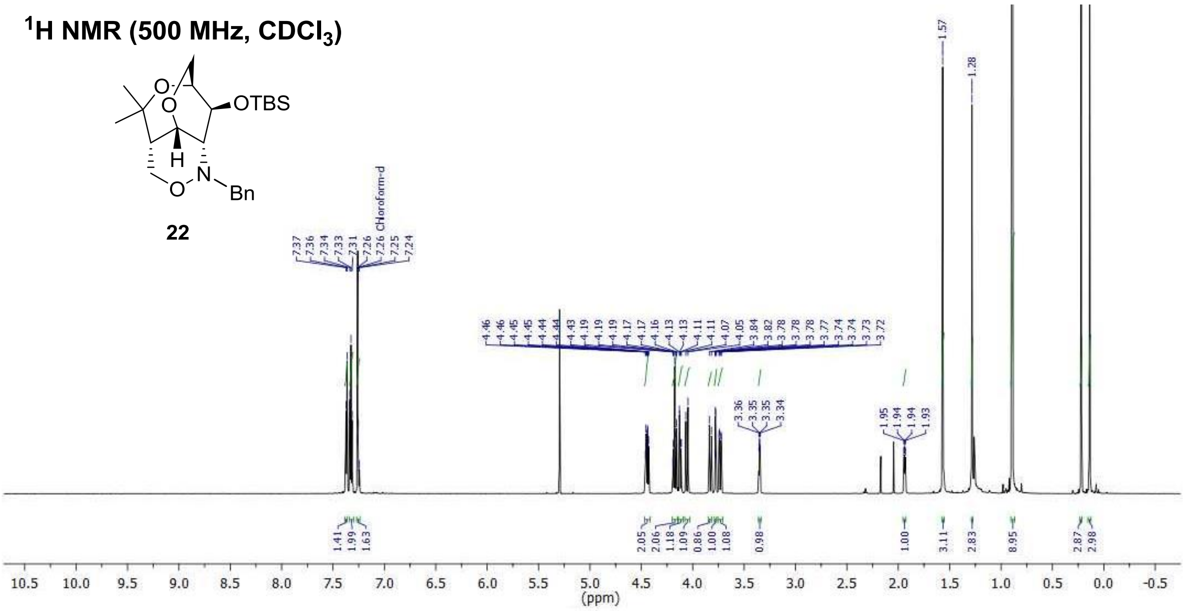

^{13}C NMR (125 MHz, $CDCl_3$)

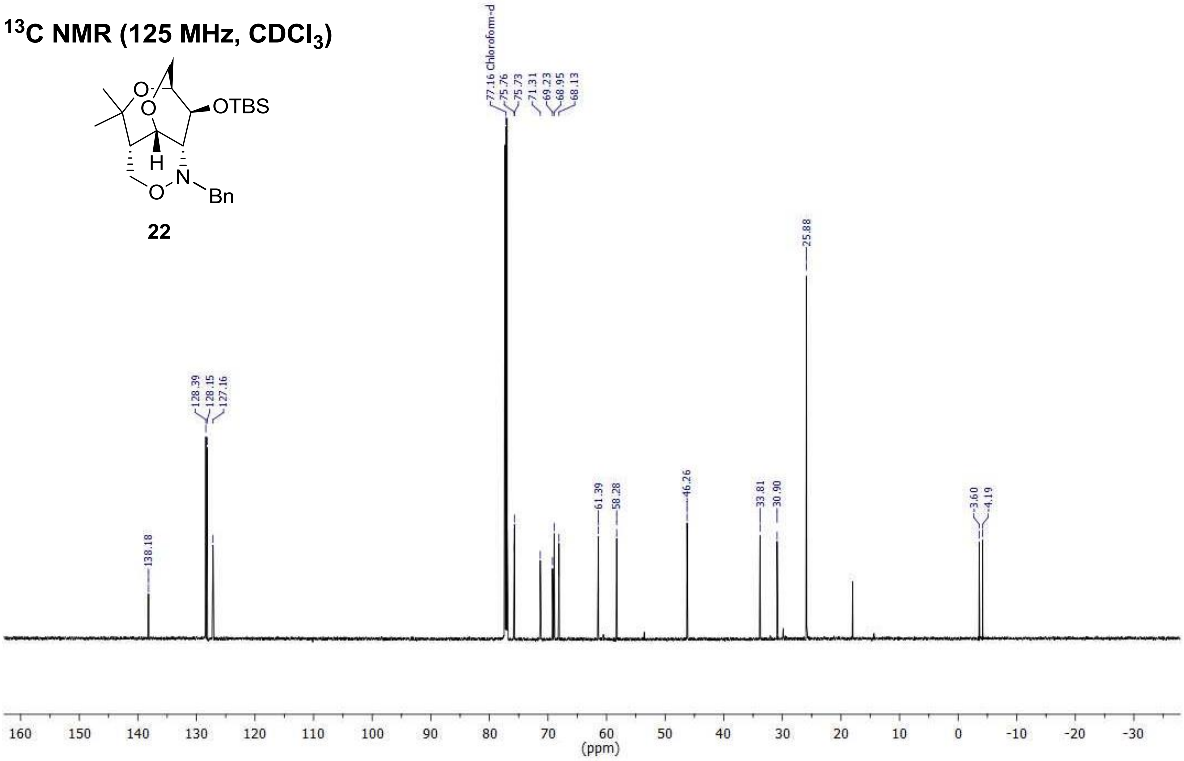

^{1}H NMR (500 MHz, $CDCl_3$)

17

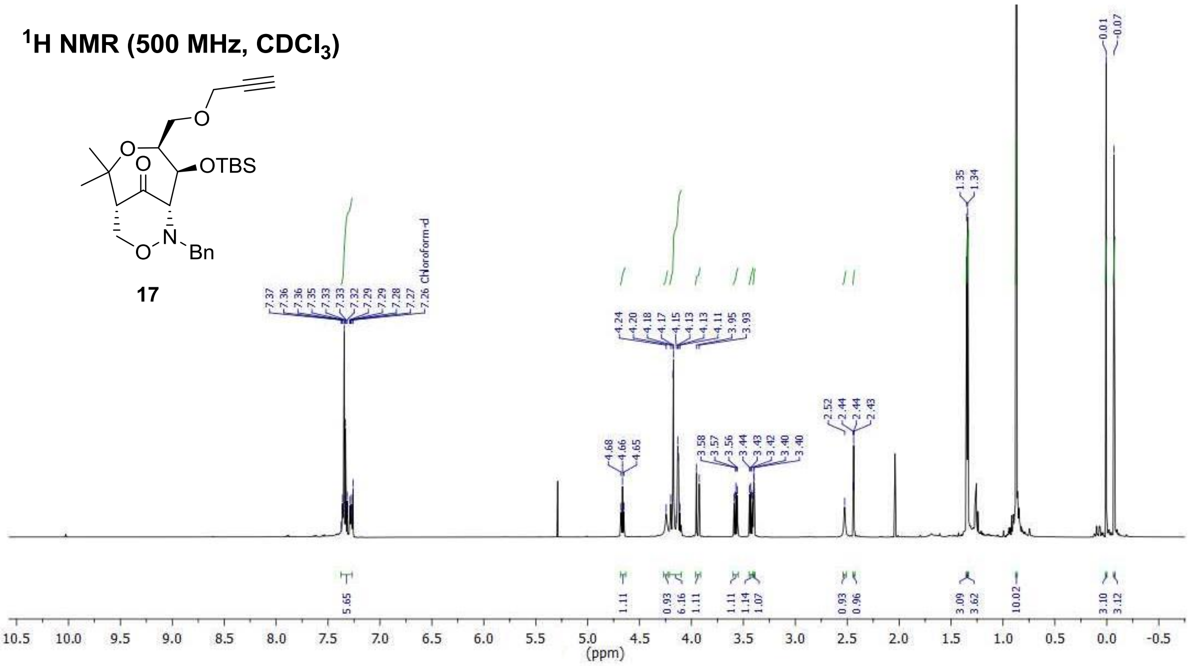

^{13}C NMR (125 MHz, $CDCl_3$)

17

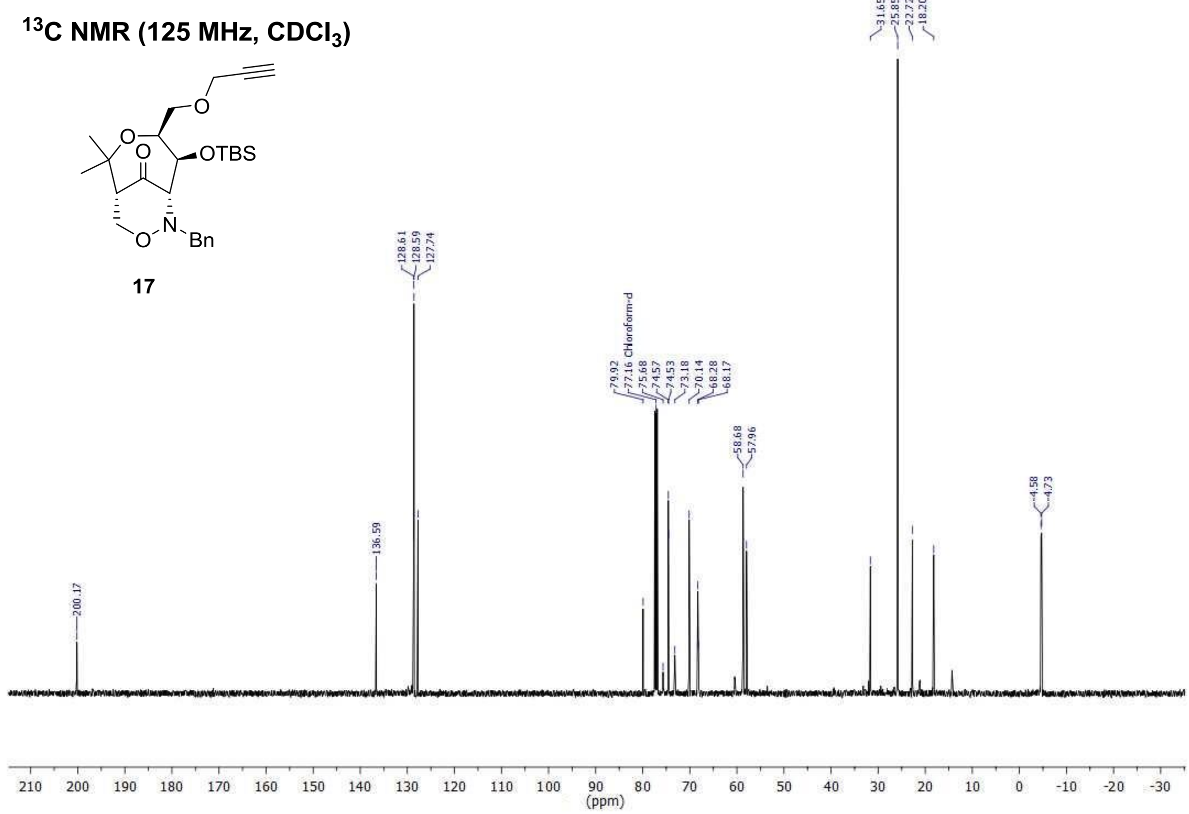

^1H NMR (500 MHz, $CDCl_3$)

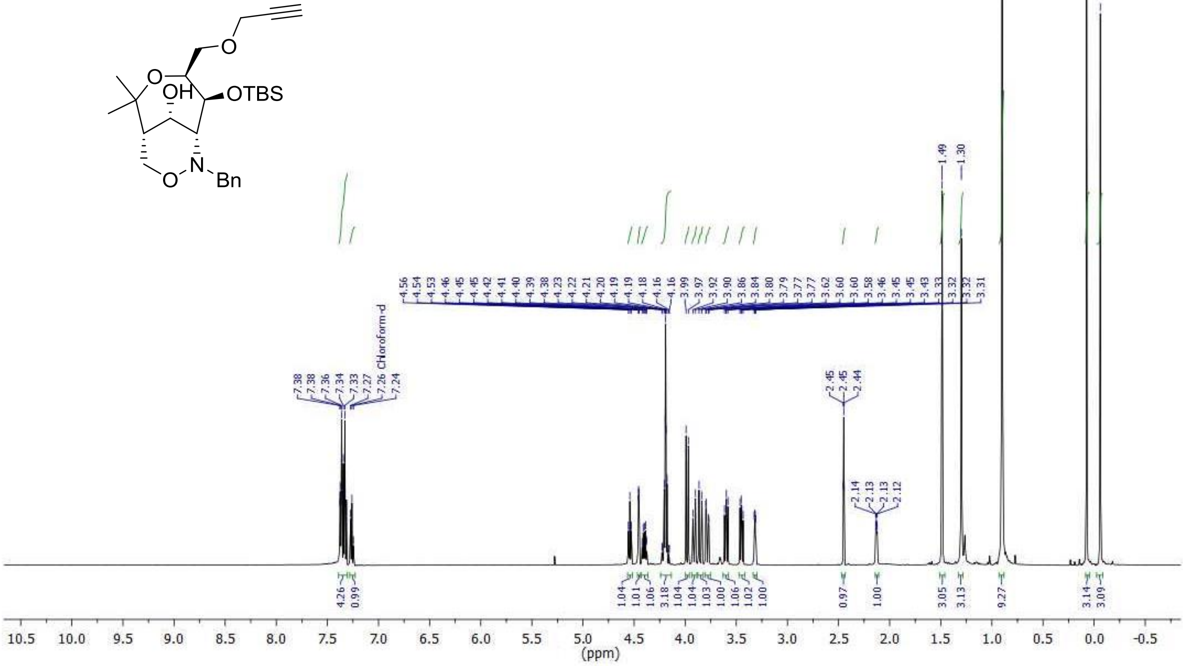

^{13}C NMR (125 MHz, $CDCl_3$)

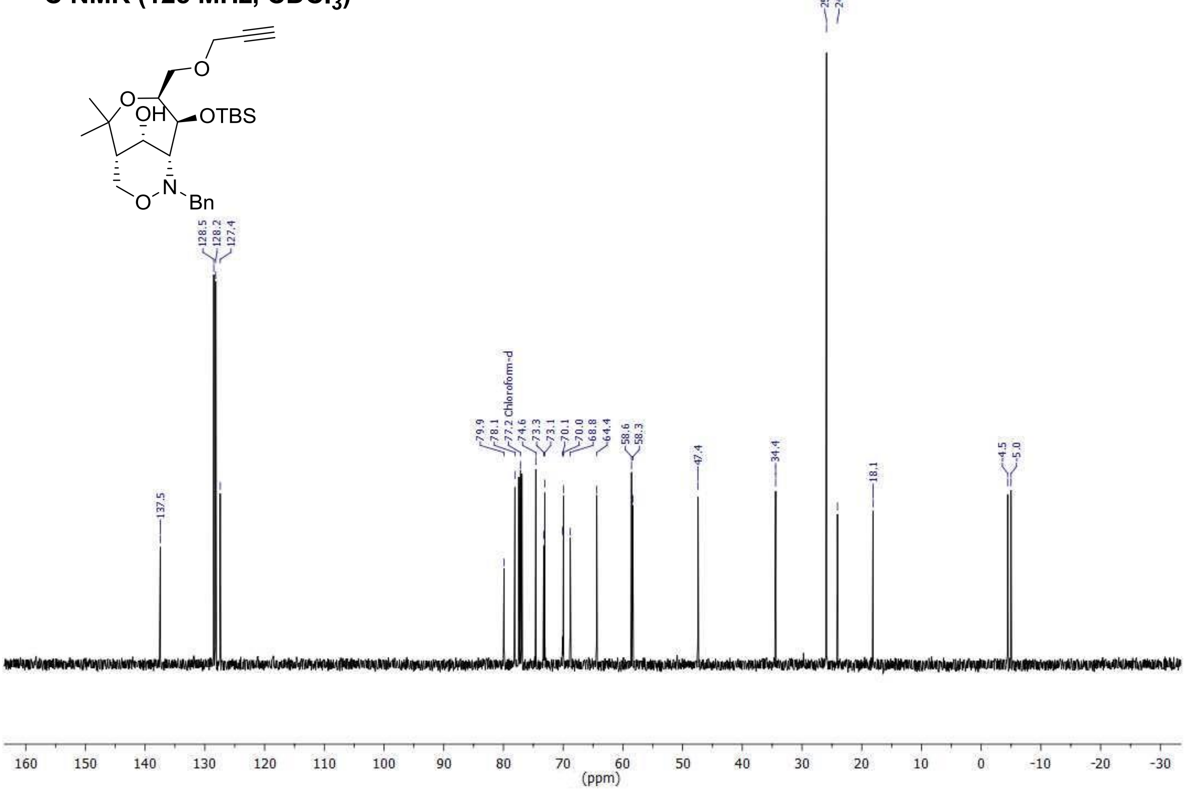

^{1}H NMR (500 MHz, $CDCl_3$)

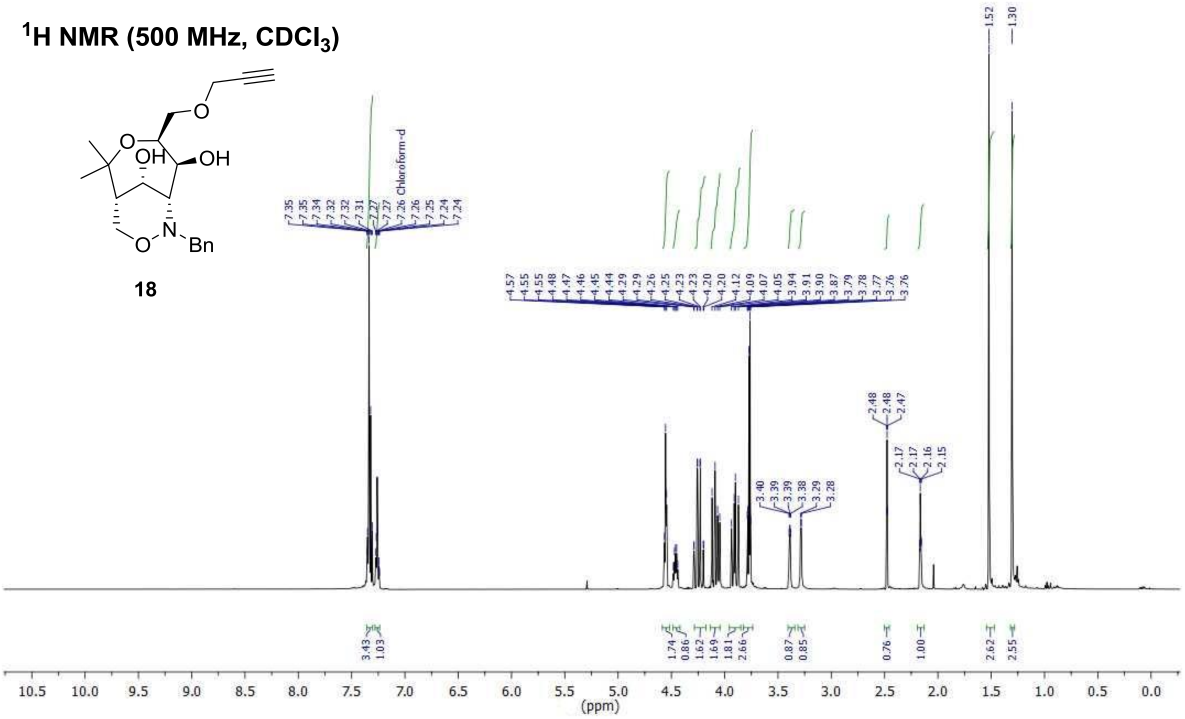

18

^{13}C NMR (125 MHz, $CDCl_3$)

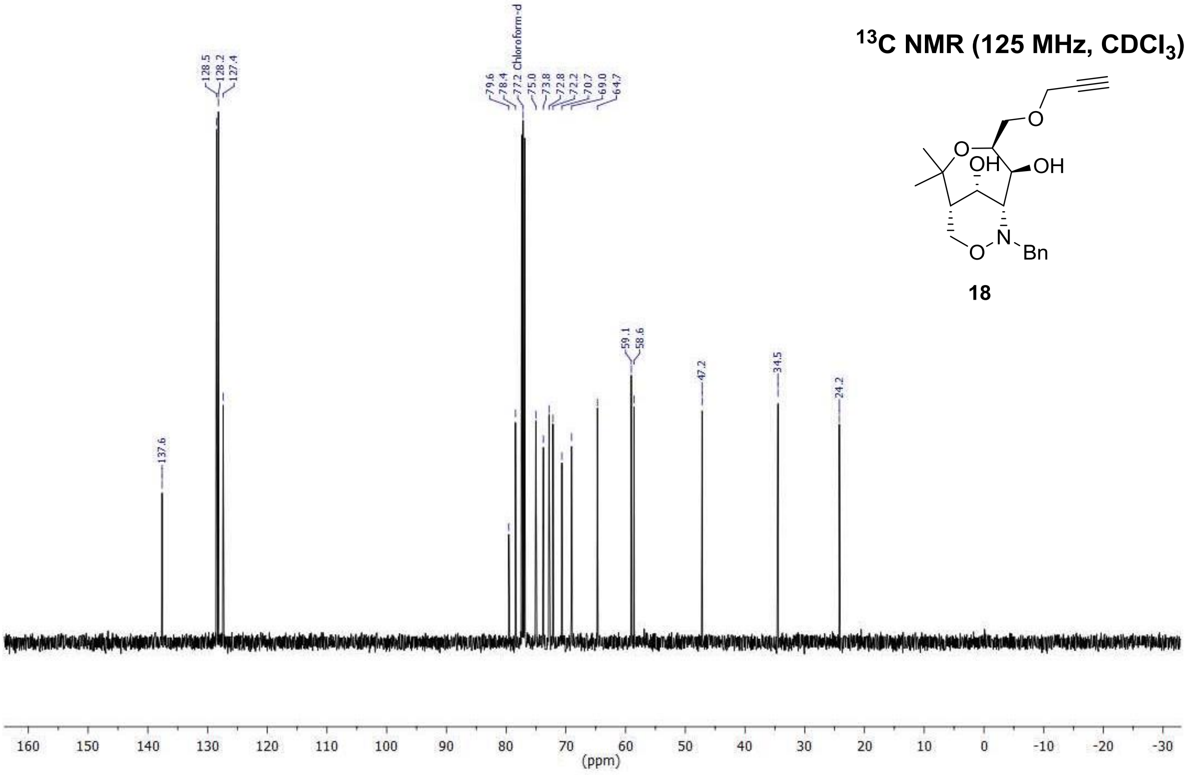

18

^{1}H NMR (500 MHz, $CHCl_3$)

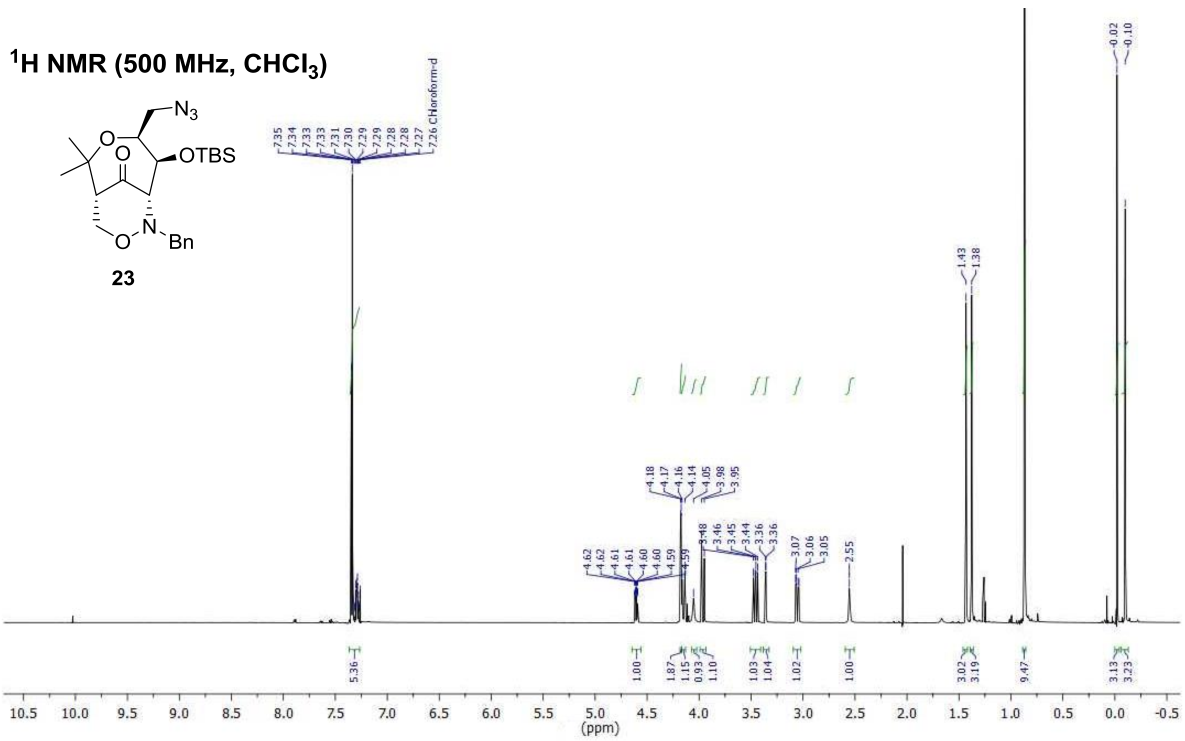

^{13}C NMR (125 MHz, $CDCl_3$)

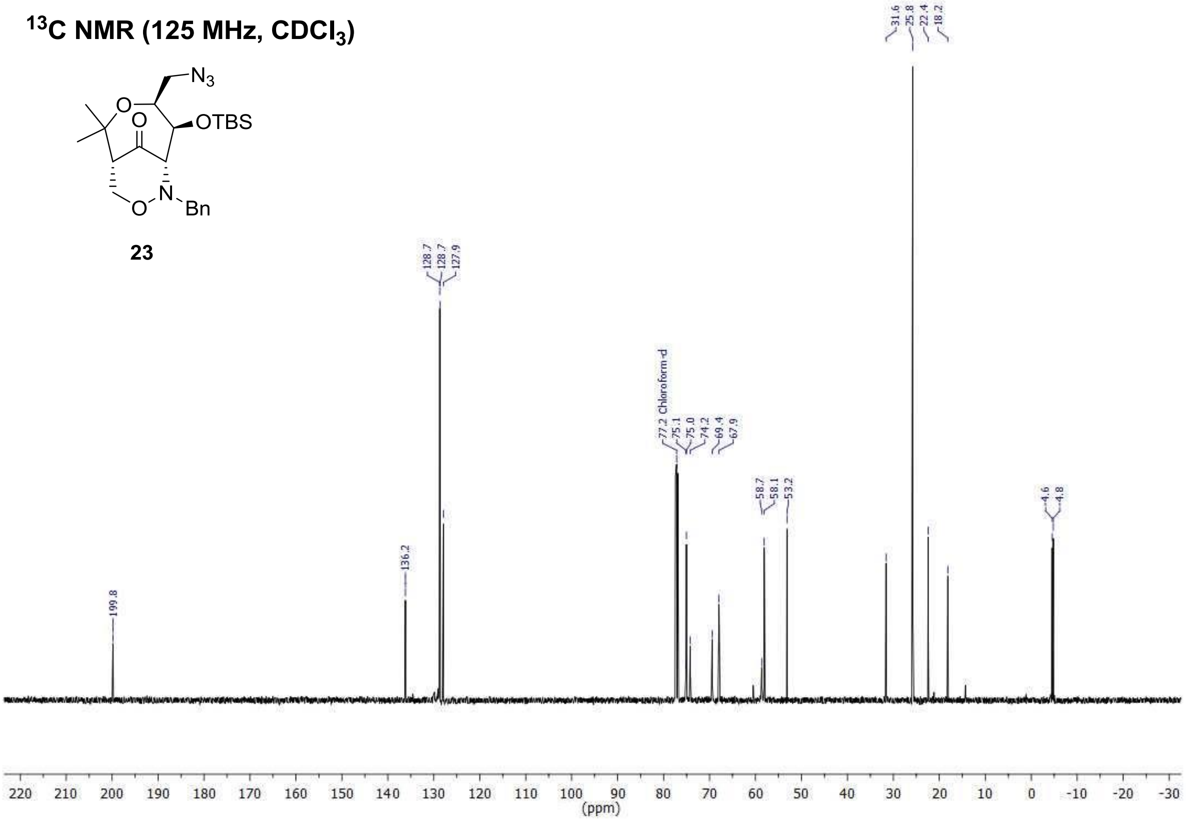

^{1}H NMR (700 MHz, CDCl$_3$)

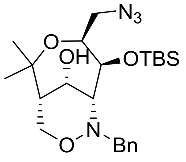

21

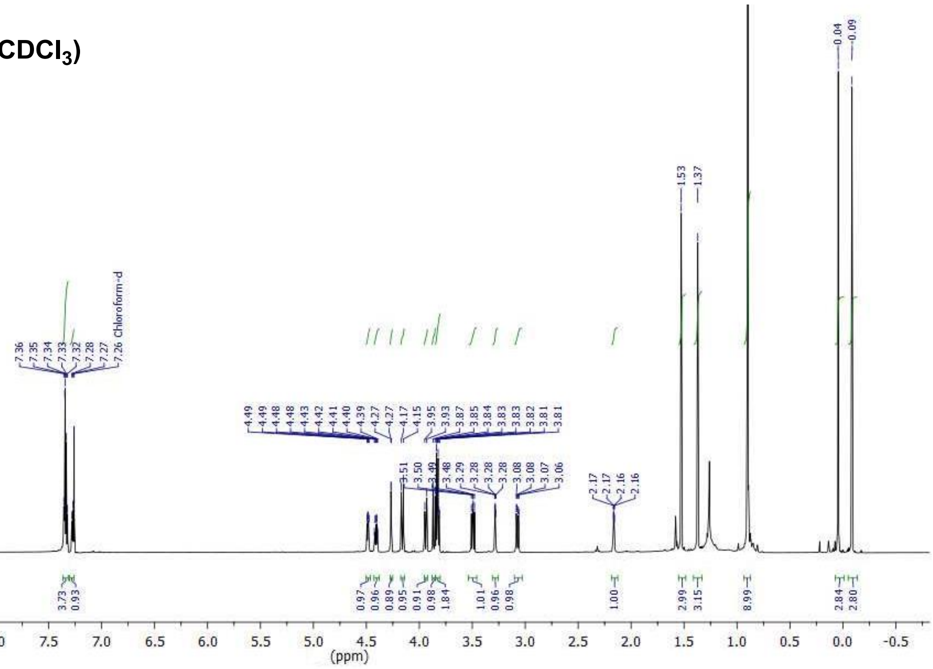

^{13}C NMR (175 MHz, CDCl$_3$)

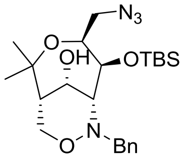

21

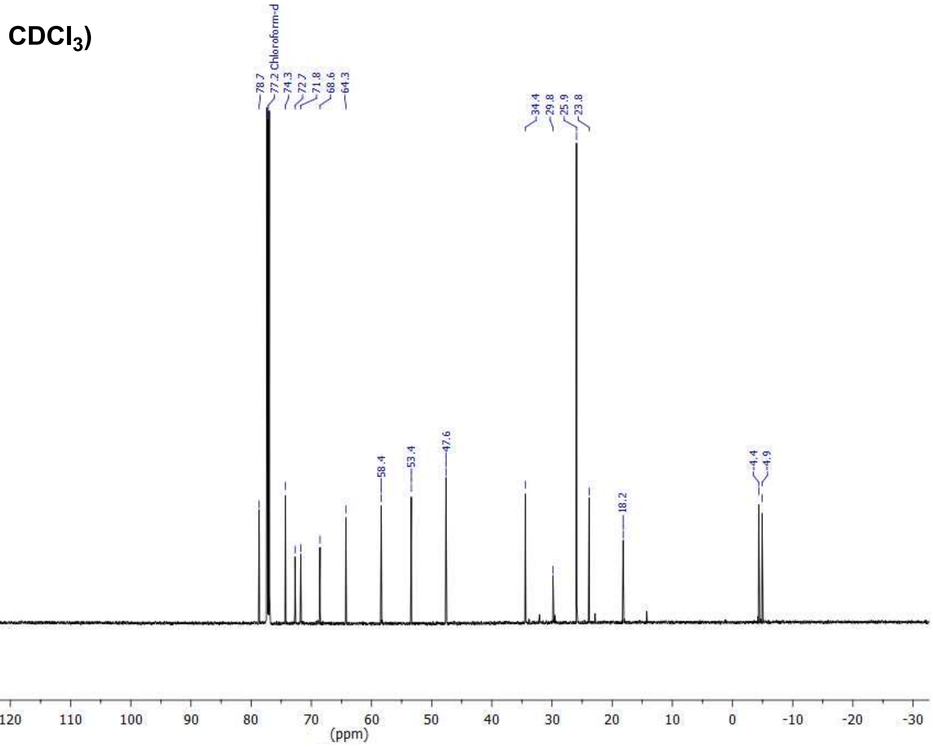

^{1}H NMR (500 MHz, CDCl$_3$)

24

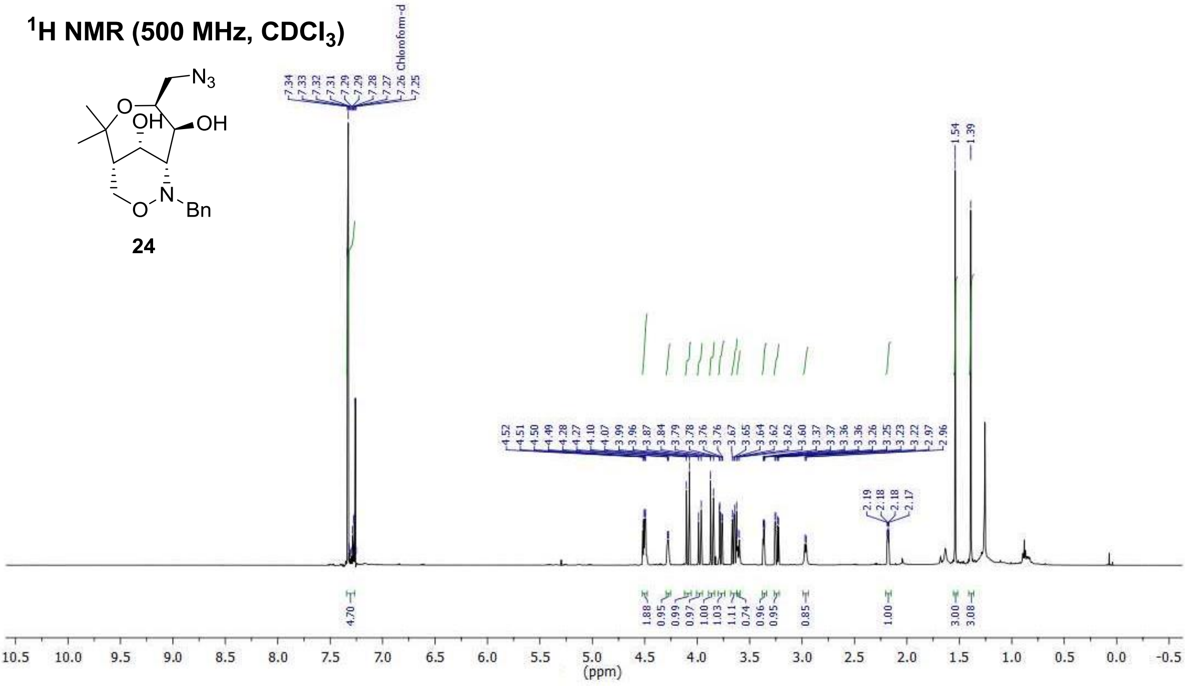

^{13}C NMR (125 MHz, CDCl$_3$)

24

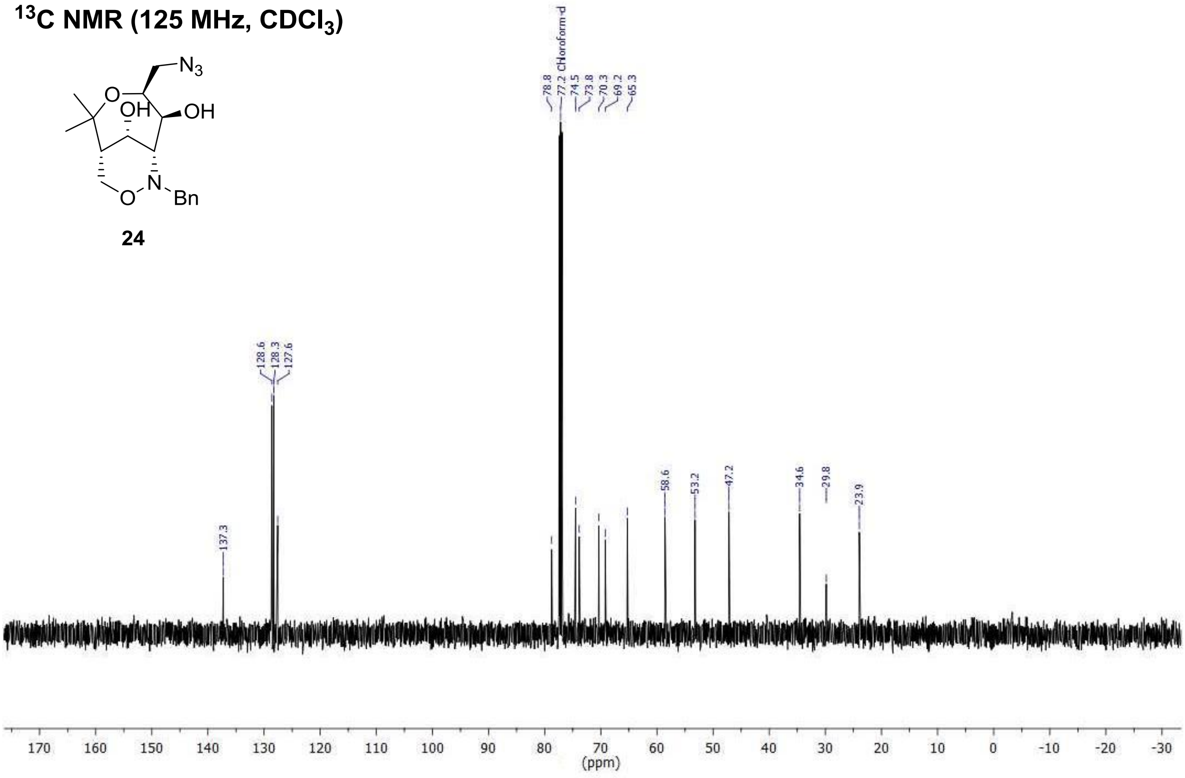

^{1}H NMR (500 MHz, $CDCl_3$)

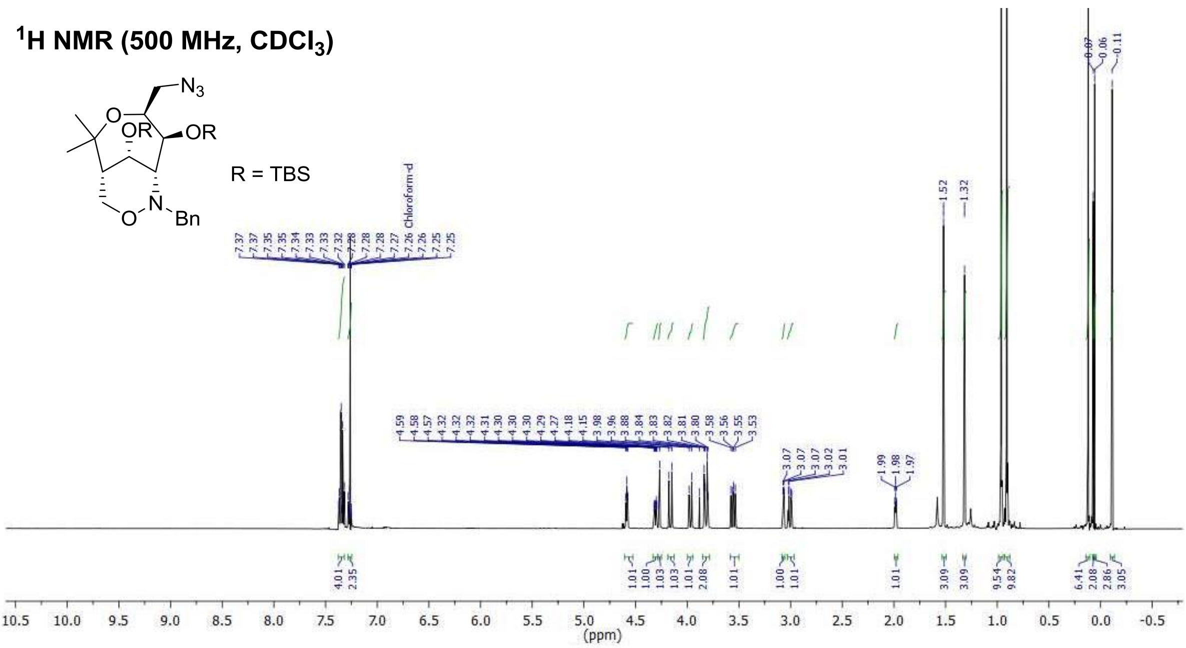

^{13}C NMR (125 MHz, $CDCl_3$)

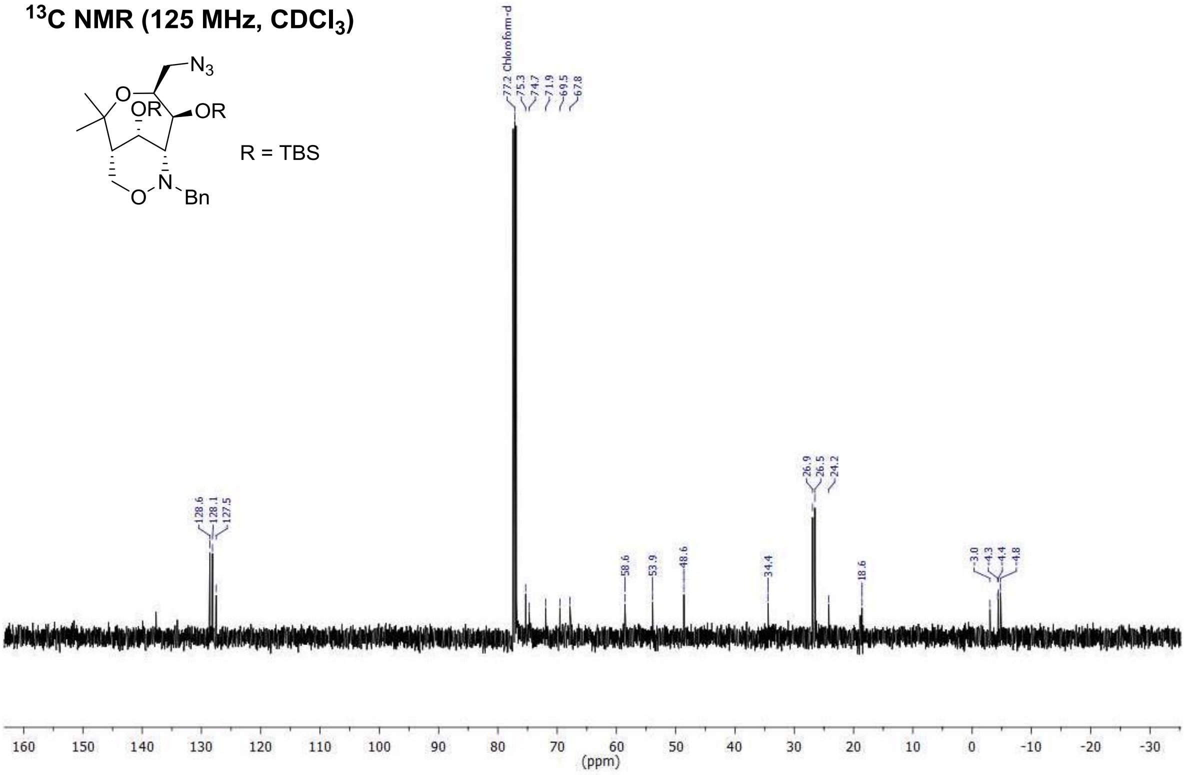

^{1}H NMR (500 MHz, $CDCl_3$)

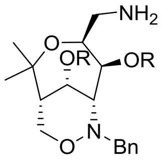

R = TBS

25

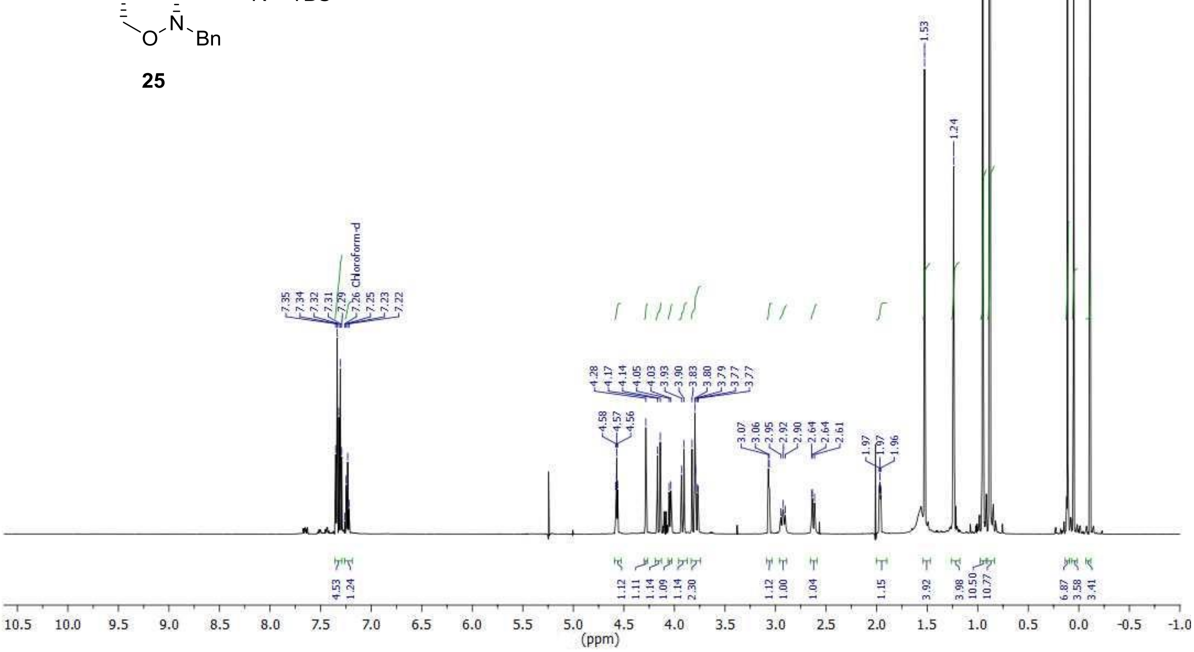

^{13}C NMR (125 MHz, $CDCl_3$)

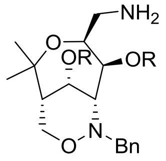

R = TBS

25

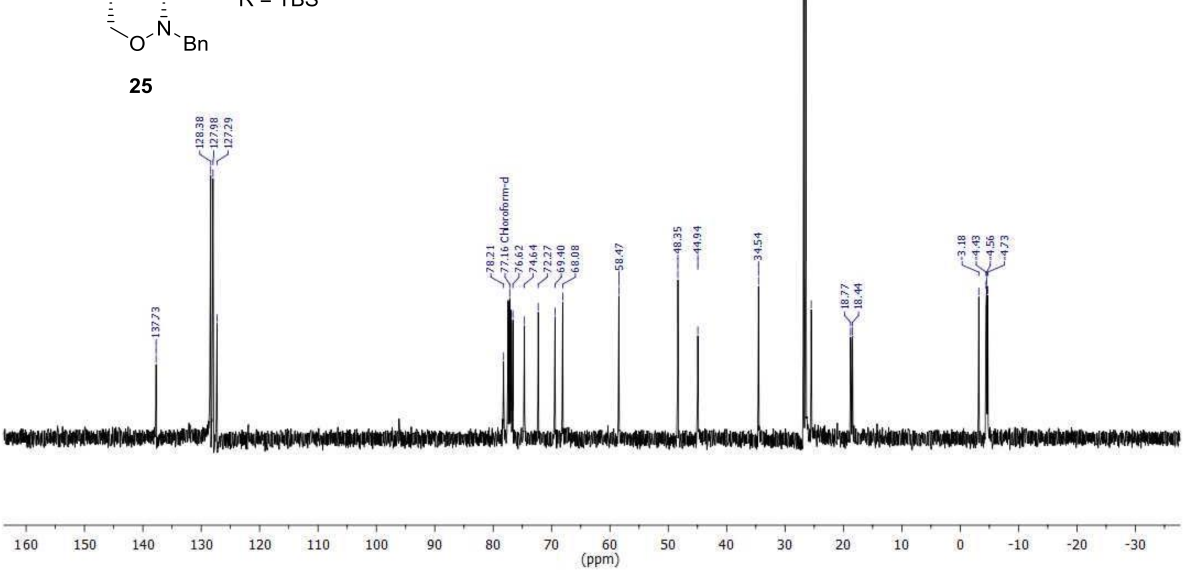

^{1}H NMR (700 MHz, CD_3OD)

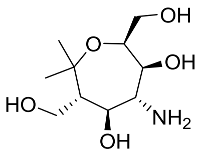

26

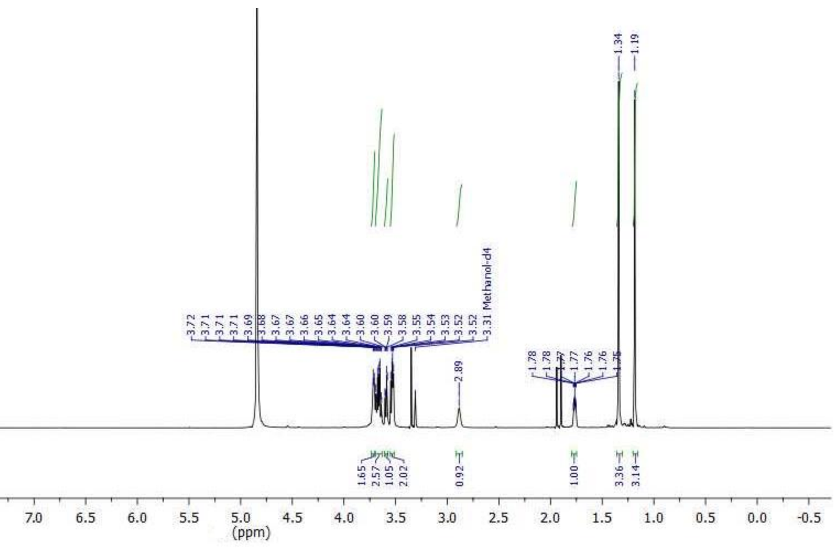

^{13}C NMR (175 MHz, CD_3OD)

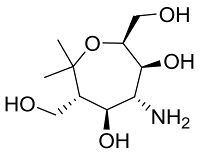

26

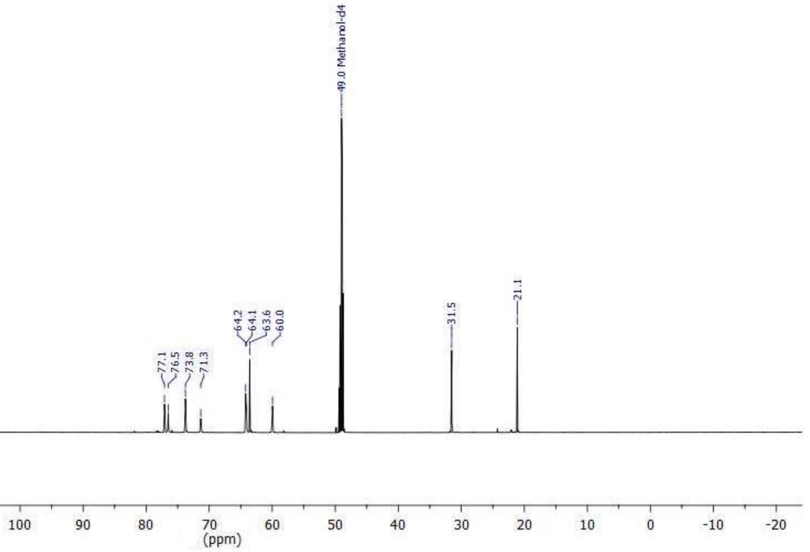

^{1}H NMR (700 MHz, CD$_3$OD)

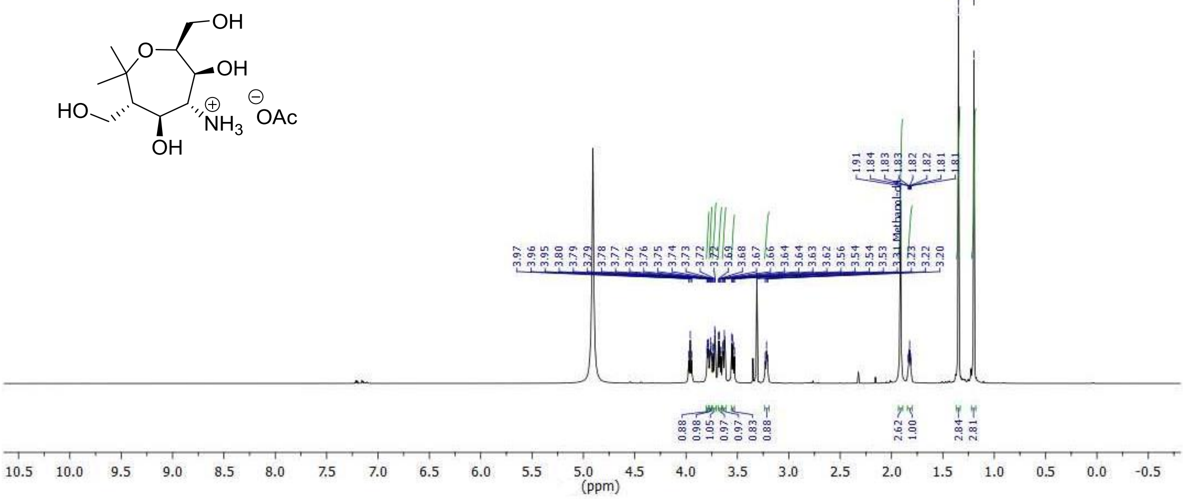

^{13}C NMR (175 MHz, CD$_3$OD)

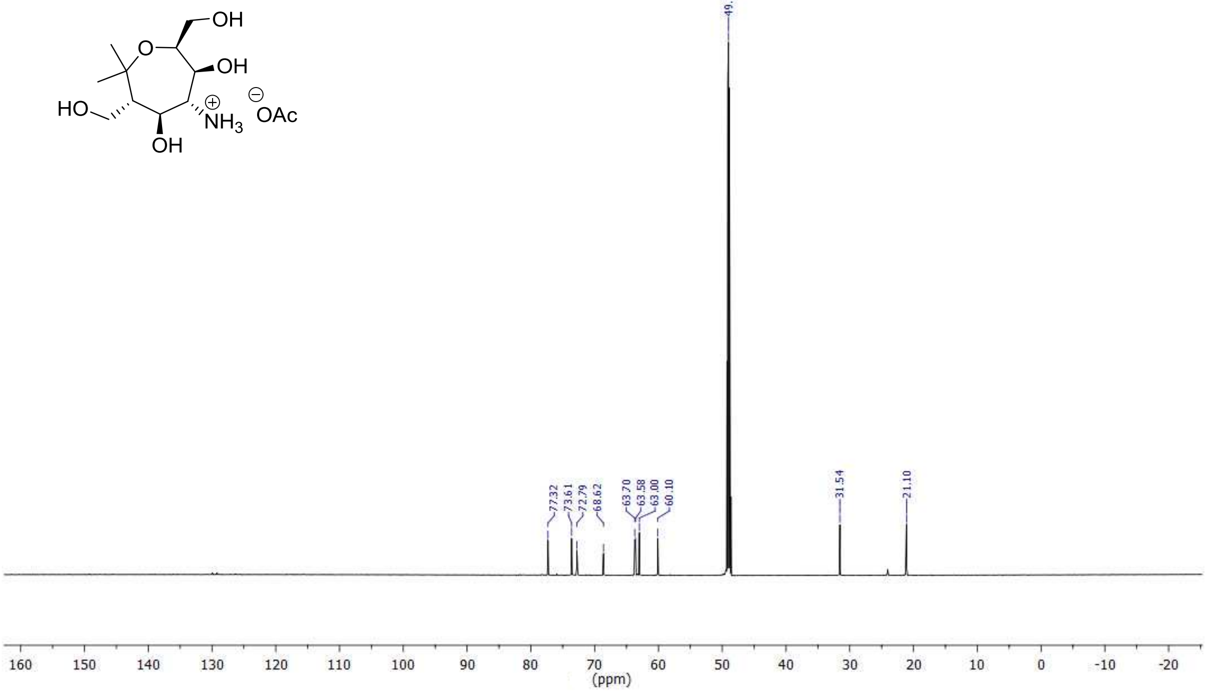

^{1}H NMR (700 MHz, CD_3OD)

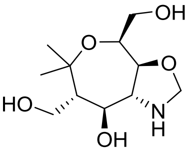

29

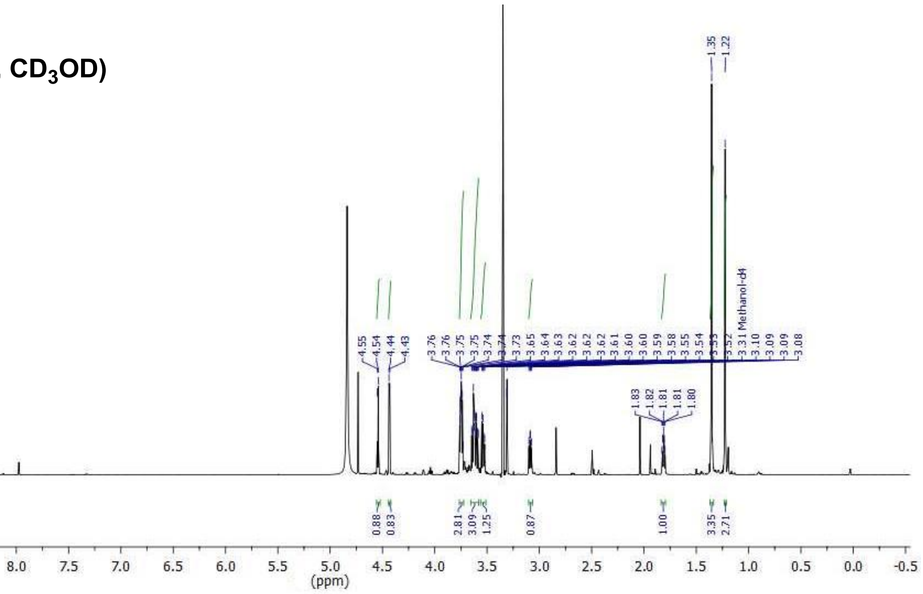

^{13}C NMR (175 MHz, CD_3OD)

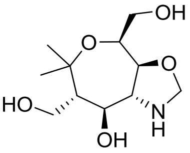

29

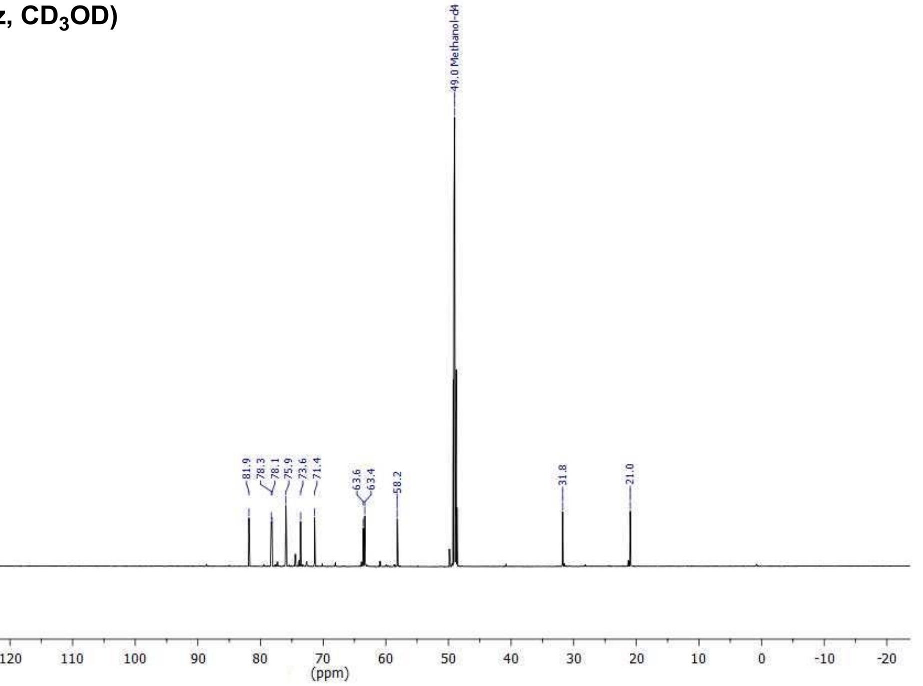

^{1}H NMR (700 MHz, CD_3OD)

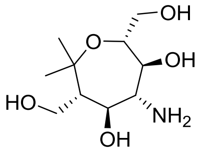

27

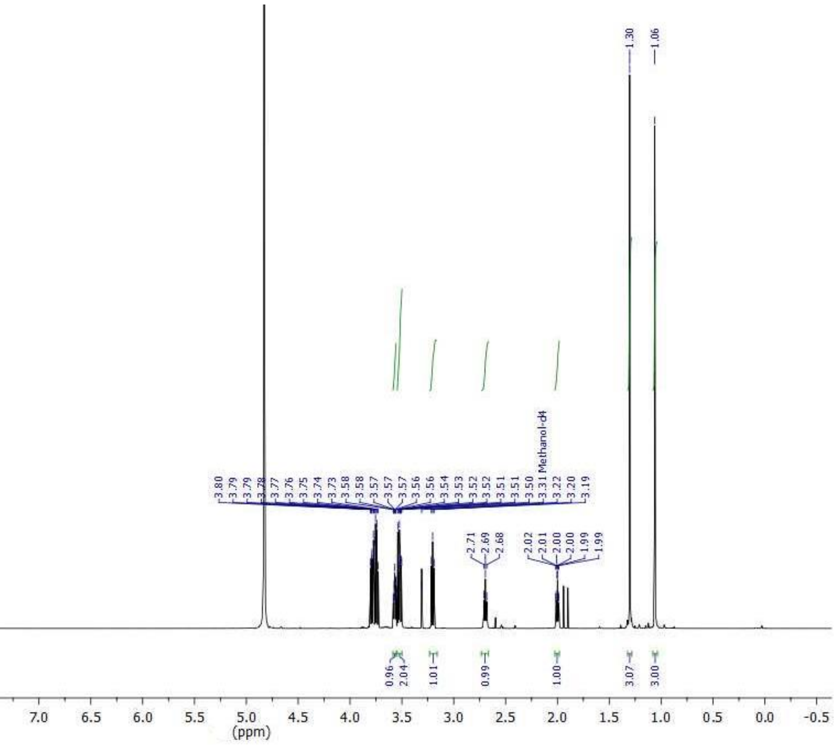

^{13}C NMR (175 MHz, CD_3OD)

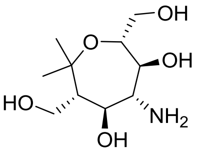

27

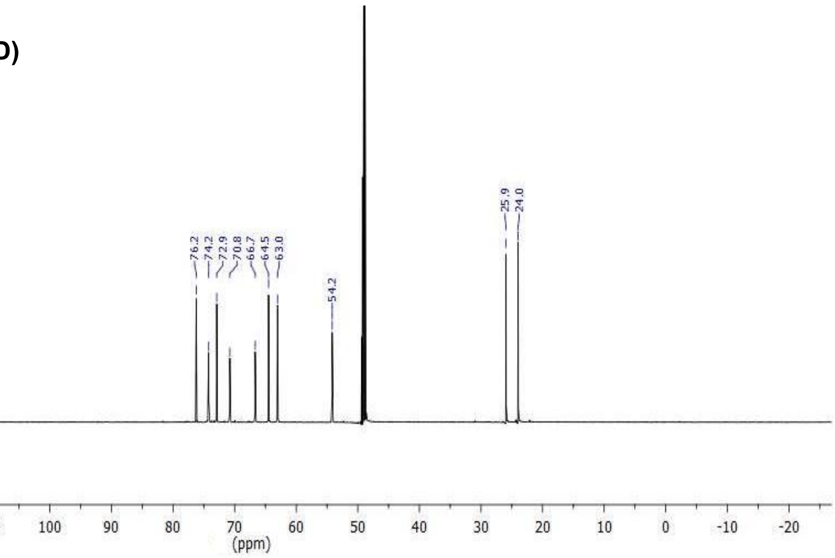

^{1}H NMR (500 MHz, CD_3OD)

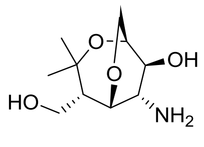

28

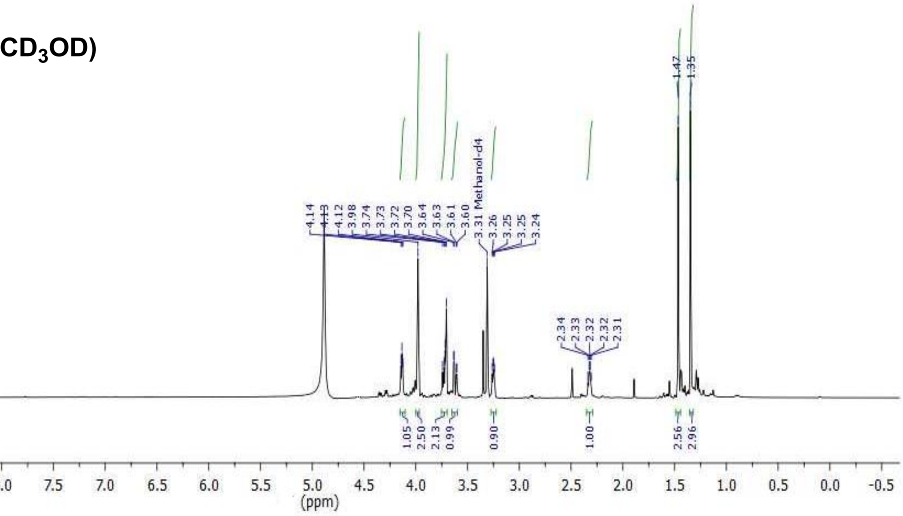

^{13}C NMR (125 MHz, CD_3OD)

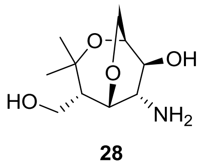

28

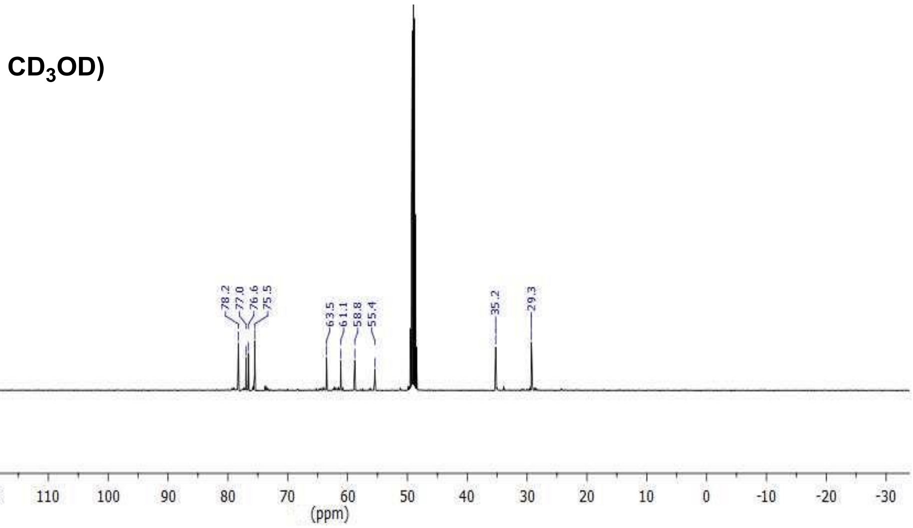

^{1}H NMR (500 MHz, py-d$_5$)

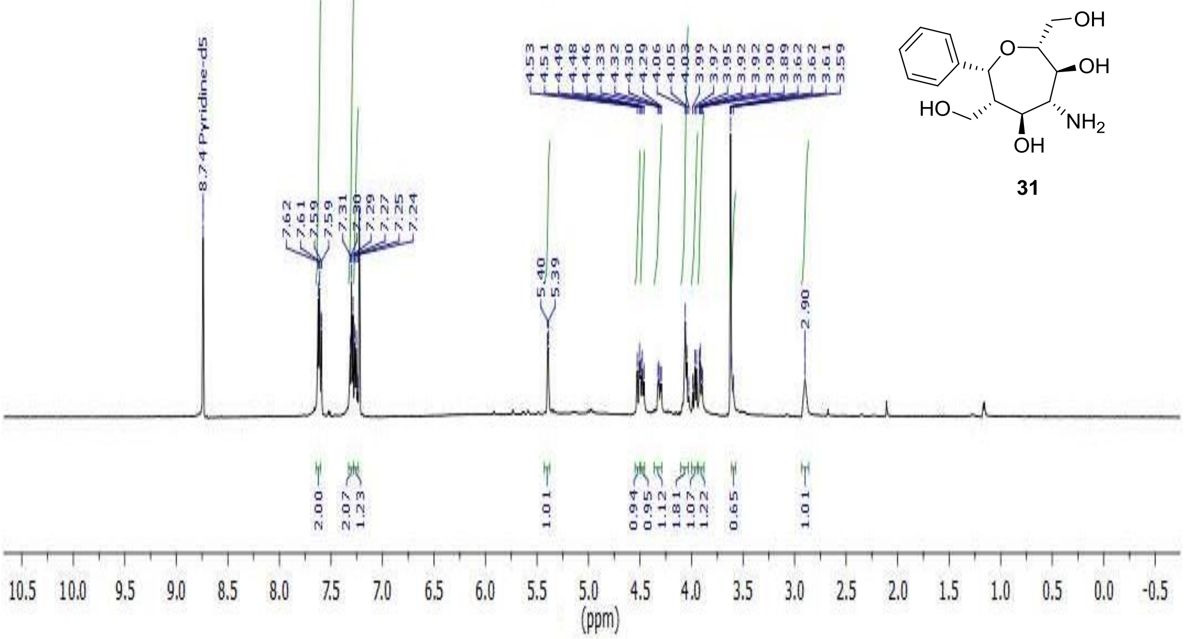

^{13}C NMR (125 MHz, py-d$_5$)

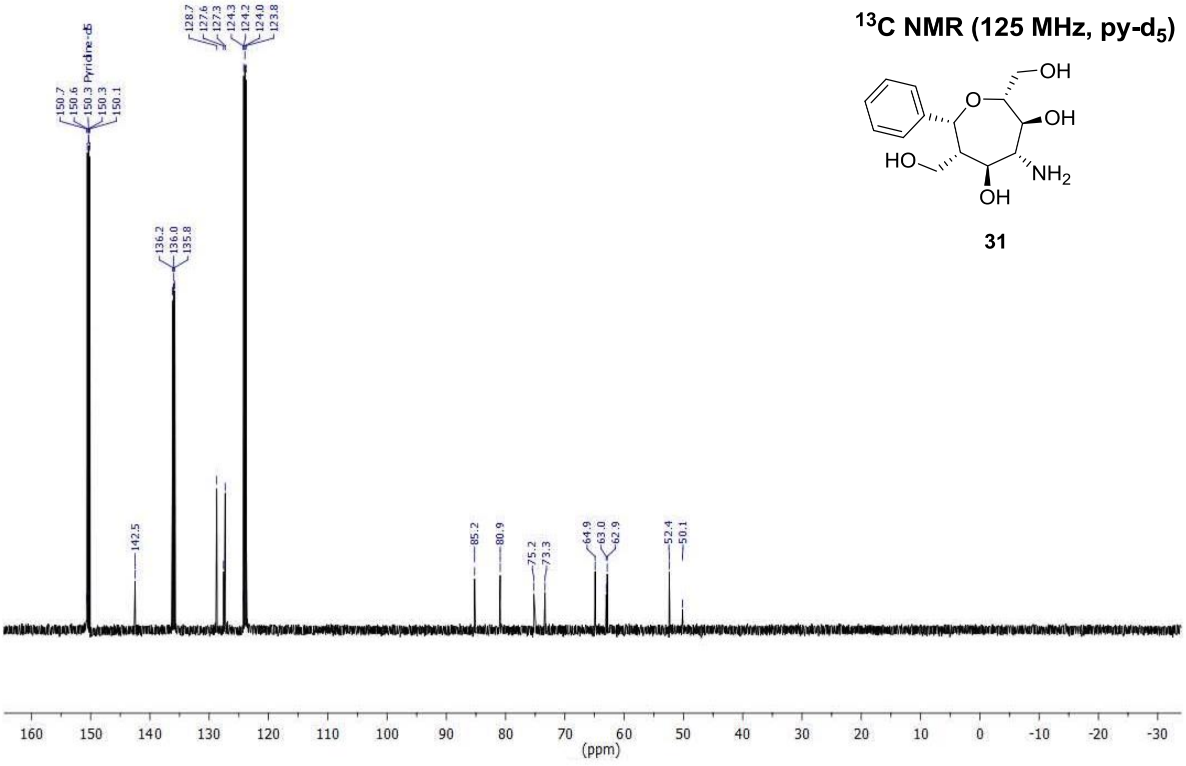

^{1}H NMR (700 MHz, CD_3OD)

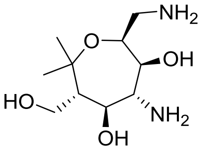

33

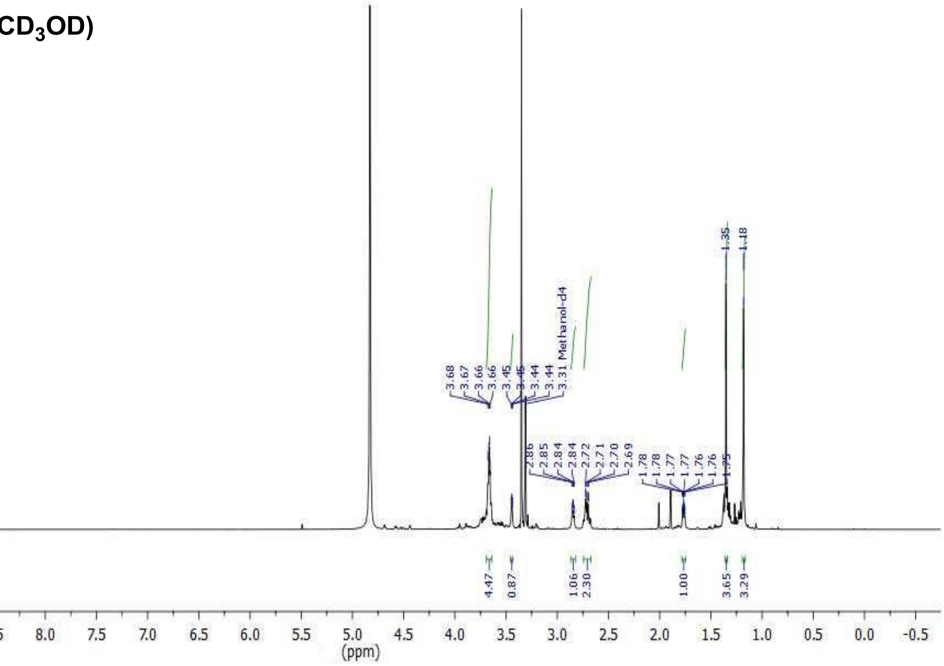

^{13}C NMR (175 MHz, CD_3OD)

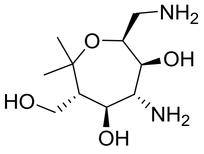

33

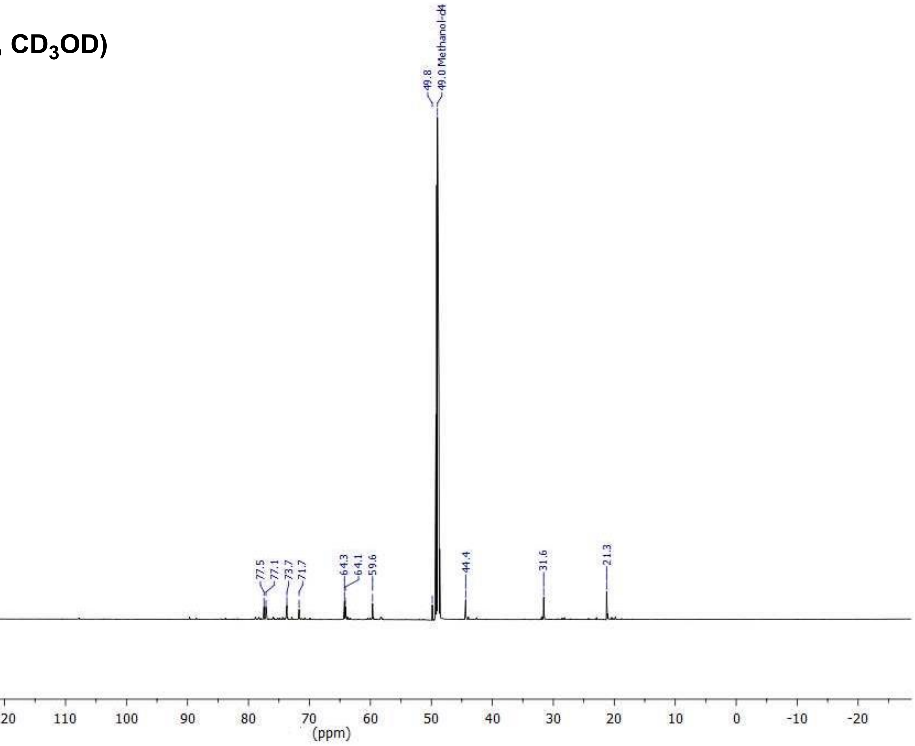

^{1}H NMR (500 MHz, $CDCl_3$)

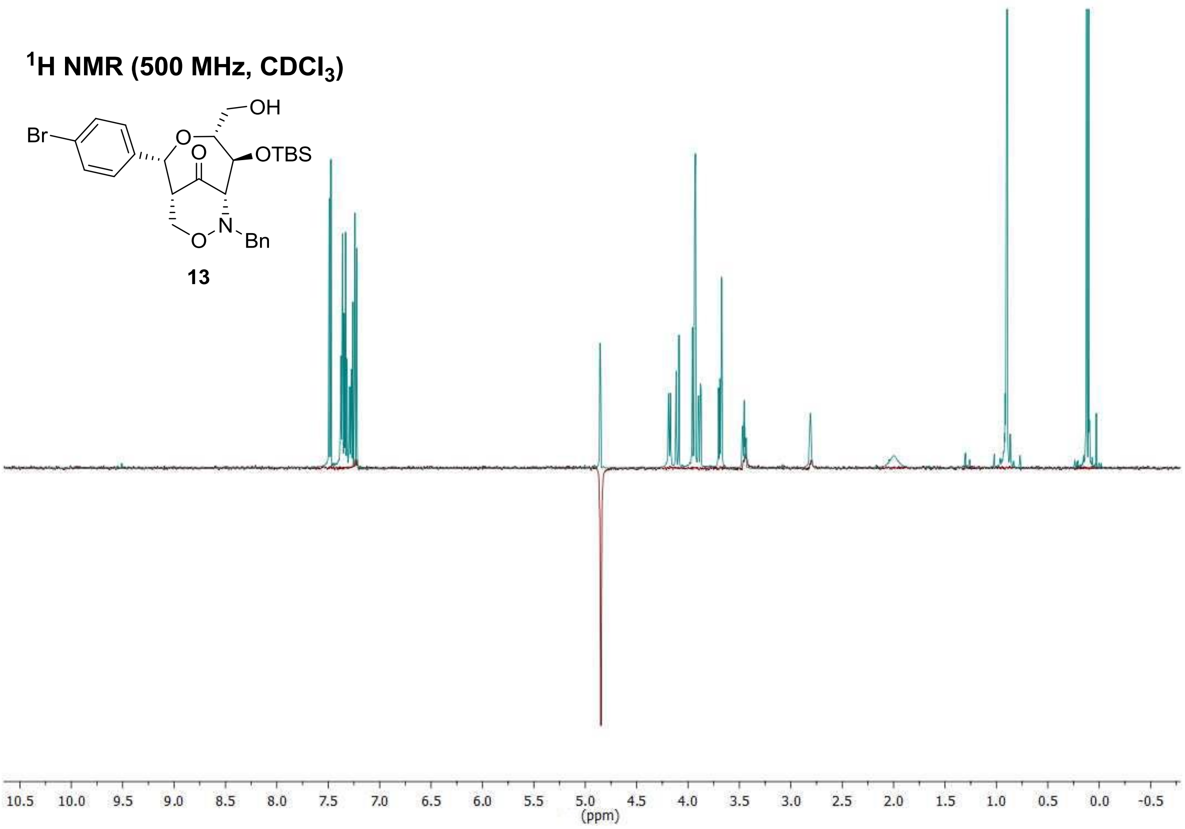

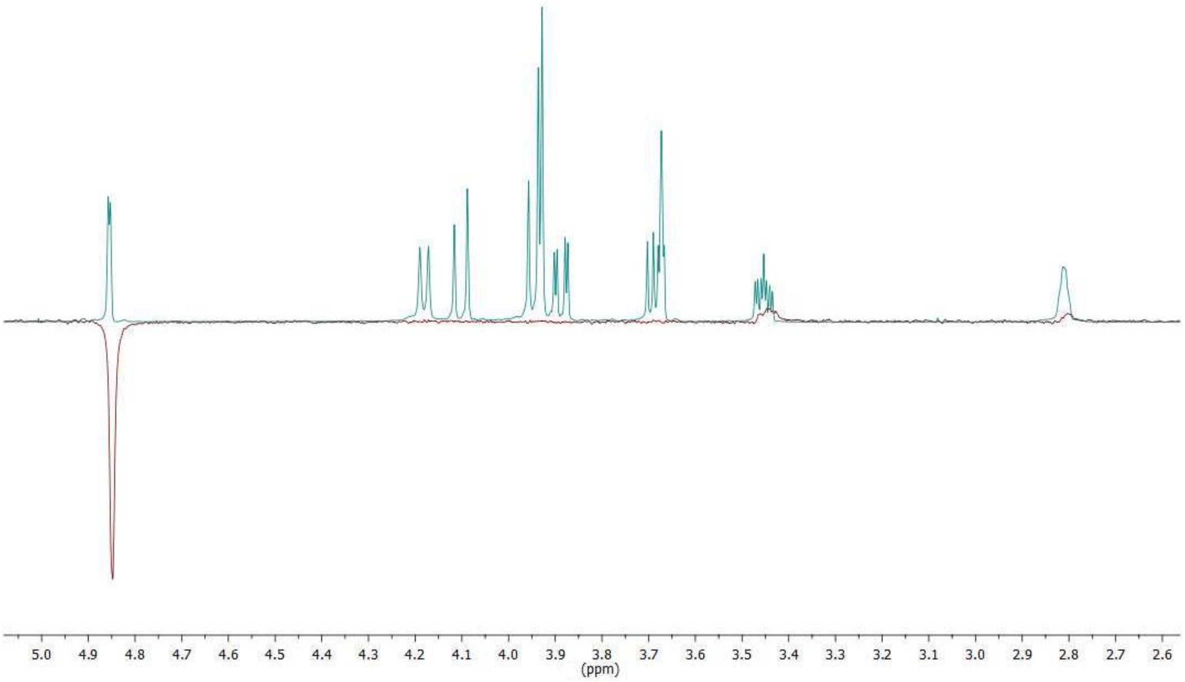

^{1}H NMR (500 MHz, $CDCl_3$)

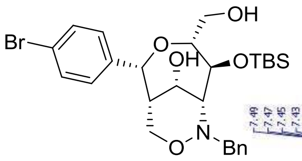

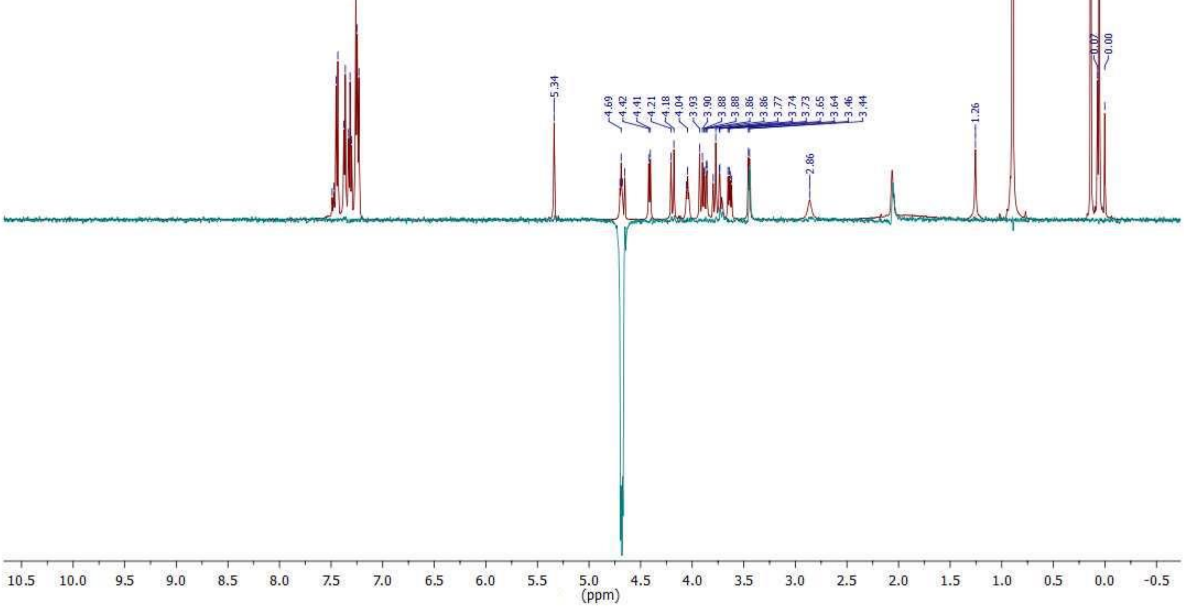

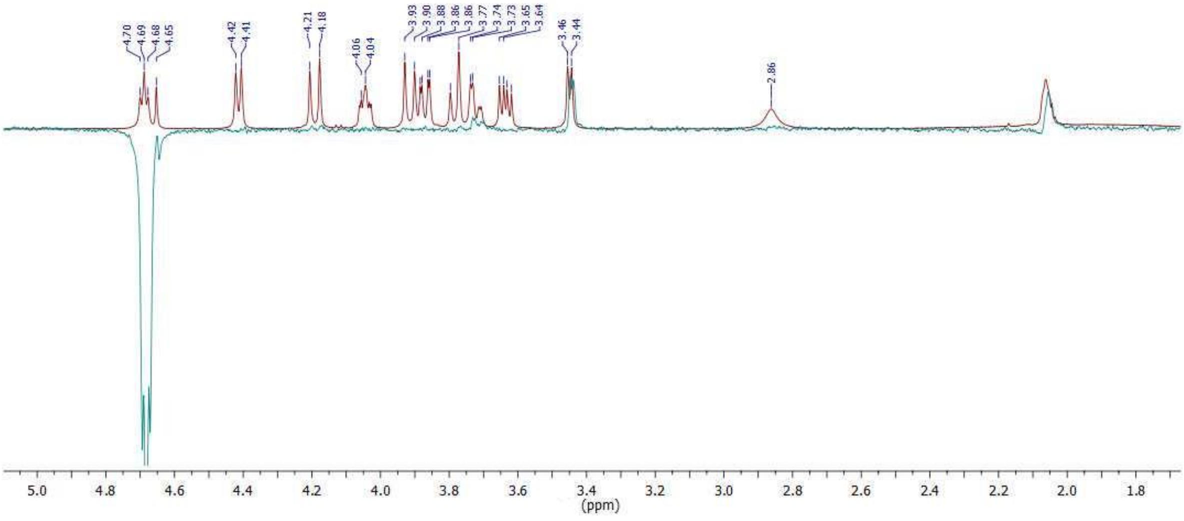

Supplement: File 2 — Characterization data 1H NMR and 13C NMR spectra of synthesized compounds. [file Beilstein_J_Org_Chem-10-213-s002.pdf]
